# ERR2 and ERR3 promote the development of gamma motor neuron functional properties required for proprioceptive movement control

**DOI:** 10.1371/journal.pbio.3001923

**Published:** 2022-12-21

**Authors:** Mudassar N. Khan, Pitchaiah Cherukuri, Francesco Negro, Ashish Rajput, Piotr Fabrowski, Vikas Bansal, Camille Lancelin, Tsung-I Lee, Yehan Bian, William P. Mayer, Turgay Akay, Daniel Müller, Stefan Bonn, Dario Farina, Till Marquardt

**Affiliations:** 1 Interfaculty Chair for Neurobiological Research, RWTH Aachen University: Medical Faculty (UKA), Clinic for Neurology & Faculty for Mathematics, Computer and Natural Sciences, Institute for Biology 2, Aachen, Germany; 2 Developmental Neurobiology Laboratory, European Neuroscience Institute (ENI-G), Göttingen, Germany; 3 Leibniz-Forschungsinstitut für Molekulare Pharmakologie (FMP), Berlin, Germany; 4 SRM University Andhra Pradesh, Mangalagiri-Mandal, Neeru Konda, Amaravati, Andhra Pradesh, India; 5 Department of Clinical and Experimental Sciences, Università degli Studi di Brescia, Brescia, Italy; 6 University Medical Center Hamburg Eppendorf, Center for Molecular Neurobiology Hamburg (ZMNH), Institute of Medical Systems Biology, Hamburg, Germany; 7 Maximon AG, Zug, Switzerland; 8 Biomedical Data Science and Machine Learning Group, German Center for Neurodegenerative Diseases, Tübingen, Germany; 9 Atlantic Mobility Action Project, Brain Repair Centre, Department of Medical Neuroscience, Dalhousie University, Halifax, Nova Scotia, Canada; 10 Department of Bioengineering, Imperial College London, Royal School of Mines, London, United Kingdom; California Institute of Technology, UNITED STATES

## Abstract

The ability of terrestrial vertebrates to effectively move on land is integrally linked to the diversification of motor neurons into types that generate muscle force (alpha motor neurons) and types that modulate muscle proprioception, a task that in mammals is chiefly mediated by gamma motor neurons. The diversification of motor neurons into alpha and gamma types and their respective contributions to movement control have been firmly established in the past 7 decades, while recent studies identified gene expression signatures linked to both motor neuron types. However, the mechanisms that promote the specification of gamma motor neurons and/or their unique properties remained unaddressed. Here, we found that upon selective loss of the orphan nuclear receptors ERR2 and ERR3 (also known as ERRβ, ERRγ or NR3B2, NR3B3, respectively) in motor neurons in mice, morphologically distinguishable gamma motor neurons are generated but do not acquire characteristic functional properties necessary for regulating muscle proprioception, thus disrupting gait and precision movements. Complementary gain-of-function experiments in chick suggest that ERR2 and ERR3 could operate via transcriptional activation of neural activity modulators to promote a gamma motor neuron biophysical signature of low firing thresholds and high firing rates. Our work identifies a mechanism specifying gamma motor neuron functional properties essential for the regulation of proprioceptive movement control.

## Abstract 2

This study shows that the estrogen-related orphan receptors ERR2 and ERR3 promote specific functional properties in gamma motor neurons, a subset of motor neurons that mediate muscle proprioception and are necessary for executing precise movements.

## Introduction

In tetrapod vertebrates, stretch-sensitive mechanosensory organs called muscle spindles embedded in skeletal muscle inform the nervous system about muscle length, movement and position, in addition to participating in reflex control over muscle actions [1–3]. In amphibians and nonavian reptiles, spindle sensitivity during movements is maintained by axon collaterals of a type of motor neurons, called beta motor neurons [3–6], while alpha motor neurons exclusively connect to the extrafusal muscle fibers and are responsible for eliciting muscle contractions [7]. In mammals and birds, a third subtype of motor neurons, called gamma motor neurons, do not connect to the force-generating extrafusal skeletal muscle fibers but exclusively innervate the intrafusal fibers of muscle spindles [8–12]. The uncoupling of gamma motor neurons from muscle force generation and from receiving monosynaptic afferent input from muscle spindles proper [13–15] is thought to allow the nervous system to control muscle spindle-dependent proprioception independent of and in anticipation of movements [10,16]. Gamma motor neurons may thereby prime muscle spindles to signal expected changes in muscle length, allowing the nervous system to compare spindle signals generated by intended and actual movements, and to correct movements when both signals differ from each other [16–18]. Gamma motor neurons are further responsible for generating muscle tone by recruiting the force-generating alpha motor neurons through a monosynaptic spindle afferent feedback loop, thus assisting movement initiation and postural control [1,10].

Apart from their exclusive innervation of muscle spindles, the ability of gamma motor neurons to control muscle proprioception entails their acquisition of intrinsic biophysical properties distinguishing them from the muscle force-generating alpha motor neurons [19]. For instance, their low firing thresholds and ability to rapidly gear up high firing rates appear to be exquisitely suited for achieving near-instant intrafusal fiber tension for maintaining or modulating muscle spindle dynamic range [19]. Another feature allowing gamma motor neurons to effectively control muscle proprioception is their lack of monosynaptic (Ia) afferent feedback, which uncouples them from potential “short circuits” by their own actions on muscle spindle activity [13–15]. During embryonic development, combinatorial gene expression programs promote the generation of spinal motor neurons, the organization of their somas into motor columns and pools and axonal projections to peripheral targets [20], but much less is known about how motor neurons subsequently specialize to play different roles in movement generation or movement accuracy [20]. Such specialization of neurons can involve transcriptional programs that implement specific synaptic connectivity patterns, morphologies as well as fundamental biophysical properties [21,22], and which can operate in an overlapping or modular manner to establish neuron subtype identities [23]. Apart from their overall distinction from the gamma motor neurons, alpha motor neurons themselves exhibit a range of functional properties important for the adjustment of muscle force [24]. We and others have previously reported mechanisms underlying the diversification of alpha motor neurons proper, which involved both cell-autonomous actions by the noncanonical Notch ligand Dlk1 [25], as well as non-cell-autonomous signals by region-specific astrocytes [26].

Much has been learned in the past approximately 70 years about the diversification of motor neurons into alpha and gamma types and their respective contributions to movement control [8,16,19]. More recent studies have further begun to identify molecular markers facilitating histological and transcriptomic identification of gamma motor neurons, which include transcription factors, ion channel subunits, and neurotransmitter receptors [27–35] that could play roles in their specification and/or their properties. However, which of these or other yet to be identified markers would actually contribute (or how they would contribute) to the specification of gamma motor neurons or their properties remained unaddressed. Here, we studied the specification of gamma versus alpha motor neurons in mice and identify the orphan nuclear receptors ERR2 and ERR3 as determinants of gamma motor neuron-specific functional properties required for muscle spindle-dependent proprioceptive movement control.

## Results

### Electrophysiological characterization of murine gamma motor neurons

**Fig 1:**
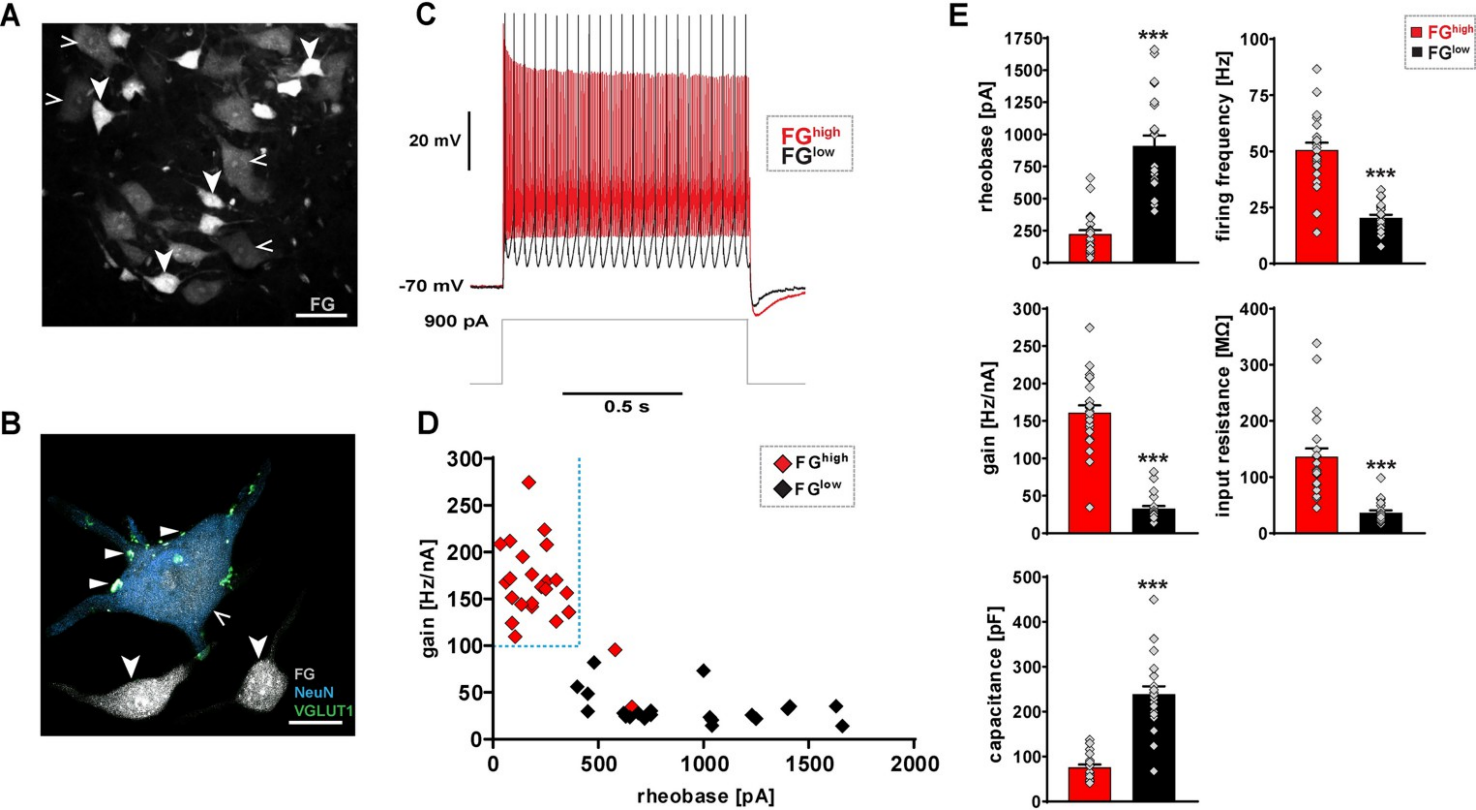
**Direct electrophysiological interrogation of alpha and gamma motor neurons (A-D)**. **(A)** Transversal section of P21 (wild-type) mouse lumbar spinal cord ventral horn: motor neurons labeled by retrograde tracer Fluoro-Gold (FG). Putative alpha motor neurons retain low levels of FG in soma (FG^low^) (open arrowheads), while putative gamma motor neurons retain high levels of FG in soma (FG^high^) (arrowheads) (scale bar: 50 μm). **(B)** *Imaris* 3D reconstruction: VGLUT1^+^ synaptic varicosities (triangles) associated with FG^low^ and NeuN^high^ alpha motor neuron (open arrowheads), but not with adjacent FG^high^ and NeuN^low or negligible^ gamma motor neurons (arrowheads) (scale bar: 20 μm). **(C)** Whole-cell patch-clamp recordings: example traces of FG^high^ (black) and FG^low^ (gray) motor neurons upon 900 pA, 1 second square current pulse. **(D)** Scatter plot: FG^high^ (*n =* 24, *N =* 16) and FG^low^ (*n* = 22, *N* = 12) motor neurons exhibit divergent gamma and alpha subtype-defining electrophysiological signatures, respectively, including gamma subtype-specific combination with low rheobase and high gain by FG^high^ motor neurons (see S1 Table for details). **(E)** FG^high^ motor neurons have significantly lower rheobase (pA) (221.87 ± 31.34), higher firing frequency (Hz) (50.65 ± 3.23), higher gain (Hz/nA) (161.01 ± 9.77), higher input resistance (136.62 ± 14.63), and lower capacitance (76.07 ± 6.01) when compared to FG^low^ motor neuron rheobase (909.09 ± 82.11), firing frequency (20.41 ± 1.70), gain (32.64 ± 4.02), input resistance (36.55 ± 4.16), and capacitance (239.1 ± 17.17), respectively (see S1 Table for details). Data are presented as mean ± SEM. *n* = # of neurons, *N* = # of mice. Statistically significant differences between FG^high^ and FG^low^ neurons are indicated as ***p < 0.001, Student *t* test). Data for Fig 1D and 1E can be found in S1 Data.

Since the last recordings from mature gamma motor neurons dated from 1978 in the cat [19], we first sought to establish a baseline for gamma motor neuron functional properties in the mouse. We were aided in this by a fortuitous finding that allowed us to selectively record gamma motor neurons in the mouse spinal cord based on the relative efficacy of retrograde Fluoro-Gold (FG) uptake (Figs 1A, 1B, and S1A–S1F), which involves systemic FG administration, endocytosis by motor presynapses at the neuromuscular junction not enveloped by the blood–brain barrier, and retrograde transport to the motor neuron somas [35]. We observed that gamma motor neurons can be distinguished from alpha motor neurons based on their FG uptake, as small-soma motor neurons with high levels of FG retention (FG^high^) lacked direct sensory neuron innervation and expressed low or negligible levels of NeuN, when compared to FG^low^ motor neurons (Figs 1B and S1A–S1F). Upon performing whole-cell patch-clamp recordings at 20 to 22 days postnatally, we observed that the FG^high^ motor neurons exhibited a biophysical signature matching that previously reported for cat gamma motor neurons [19] (Figs 1C–1E and S1G–S1I). The FG^high^ motor neurons showed a distinctive combination of lower rheobases with higher firing frequencies and gains, as well as higher instantaneous and steady-state firing rates when compared to the larger FG^low^ motor neurons (Figs 1D, 1E, S1G, and S1H and S1 Table). Moreover, FG^high^ motor neurons showed significantly lower membrane input resistance, higher membrane capacitance, and lower AHP-decay times when compared to FG^low^ motor neurons (Figs 1E and S1I and S1 Table). In contrast, FG^low^ motor neurons were characterized by overall higher rheobases and lower firing rates and gains, as well as lower instantaneous and steady-state firing rates, similar to the alpha motor neurons of the cat [24] (Fig 1D and 1E and S1 Table). In addition to the overall quantitative differences in biophysical properties between FG^high^ and FG^low^ motor neurons, FG^low^ motor neurons showed a range of combinations of biophysical properties, from relatively low rheobases plus low firing rates to high rheobases plus higher firing rates characteristic for slow, intermediate, and fast alpha motor neuron subtypes [24,25]. Gamma motor neurons in mouse therefore possess a distinctive biophysical signature matching that previously recorded for cat gamma motor neurons [19].

### Correlated expression of orphan nuclear receptors ERR2 and ERR3 by gamma motor neurons

**Fig 2:**
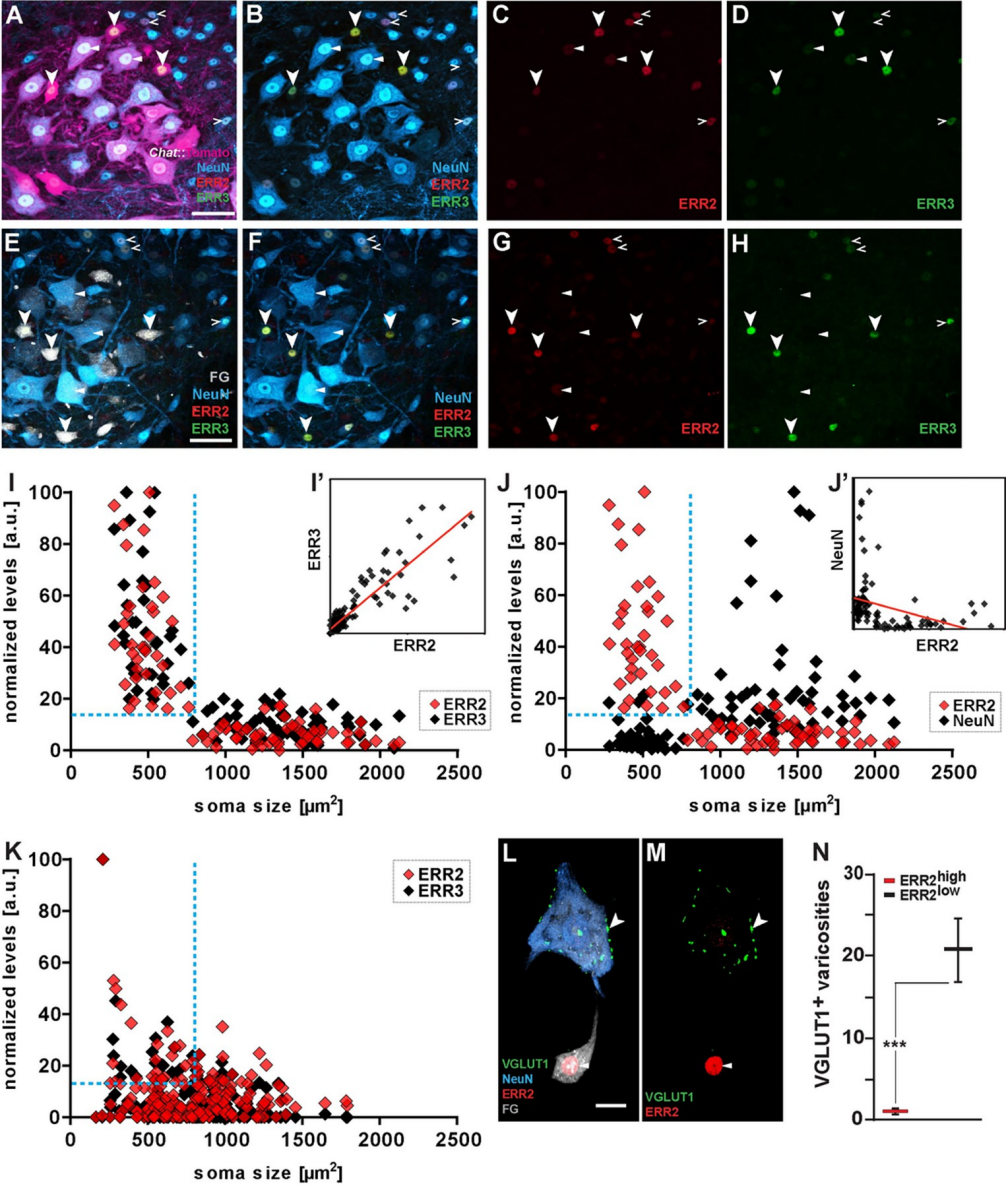
High levels of correlated ERR2/3 expression by gamma motor neurons. **(A-D)** Transversal section of P21 *Chat*^*Cre*^; *Rosa26*^*floxtdTomato*^ mouse lumbar spinal cord ventral horn: motor neurons genetically labeled by tdTomato (scale bar: 50 μm). Arrowheads: high ERR2 and ERR3 levels in small NeuN^low^, tdTomato^+^ motor neuron nuclei. Open arrowheads: relatively moderate-to-high levels ERR2/3 levels in NeuN^high^ tdTomato^−^ interneurons. Triangles: consistently lower but detectable levels in some large motor neurons with moderate-to-high NeuN levels. **(E-H)**. Transversal section of P21 (wild-type) mouse lumbar spinal cord ventral horn: motor neurons labeled by retrograde tracer Fluoro-Gold (FG) (scale bar: 50 μm). Arrowheads: high ERR2 and ERR3 levels in small NeuN^low^ that retain high levels of FG^high^. Open arrowheads: relatively moderate-to-high levels ERR2/3 levels in NeuN^high^ FG^−^ interneurons. Triangles: consistently lower but detectable levels in some large motor neurons with moderate-to-high NeuN levels. **(I)** Quantitative analysis: high ERR2 (red) and ERR3 (green) levels in motor neurons with small somas. **(I’)** L Linear regression: strong positive correlation of motor neuron Err2 and Err3 levels. (**F**) High Err2 (red) and NeuN (black) levels in nonoverlapping motor neuron populations (*n =* 93, *N =* 3, Pearson’s correlation coefficient r = 0.86). **(J)** High ERR2 (red) and low NeuN (blue) levels in motor neurons with small somas. **(J’)** Linear regression: lack of positive correlation between Err2 and NeuN levels in motor neurons (*n* = 93, *N* = 3, Pearson’s correlation coefficient r = −0.32). **(K)** Loss of high ERR2 (red) and ERR3 (green) levels by Egr3-deficient small motor neurons (*n* = 184, *N* = 3). (**L-N)** Imaris 3D reconstruction: VGLUT1^+^ synaptic varicosities associated with ERR2^low^ NeuN^high^ motor neuron, but not with adjacent ERR2^high^ NeuN^low^ motor neuron ERR2^low^ motor neuron (motor neurons retrogradely labeled by FG) (scale bar: 20 μm). (**N)** Quantification of VGLUT1^+^ synaptic varicosities associated with ERR2^high^ or ERR2^low^ motor neurons. *N* = # of mice and *n* = # of neurons. Statistically significant differences between motor neurons are indicated as: *p < 0.05, **p < 0.01, ***p < 0.001, n.s. = not significant, Student *t* test). Data for Fig 2I, 2J, 2K, and 2N can be found in S2 Data.

It had previously been established that the survival of gamma motor neurons relies on signals released by muscle spindles [27–29], which, in addition to the discovery of markers allowing in situ detection of gamma motor neurons [27–34], provided us with entry points for studying mechanisms underlying the diversification of motor neurons into alpha and gamma subtypes. We focused on the estrogen-related receptor (ERR) subfamily of orphan nuclear receptors, which primarily function as ligand-independent transcription factors [36,37], because of their contribution to cell type-specific properties in other contexts [38] and because of the previously reported expression of ERR3 by gamma motor neurons [27]. Upon immunodetection using antibodies specifically recognizing either ERR2 or ERR3 (S2A–S2Q Fig), we further found that the closely related ERR3 paralogue ERR2, with which it shares virtually the same DNA binding sequences [38], was coexpressed with ERR3 by gamma motor neurons (Figs 2A–2J and S3A–S3T), a coexpression that had been independently observed by others using single-cell RNAseq [32]. Through deeper analysis by quantitative immunodetection, we indeed found high levels of correlated expression (Pearson’s correlation coefficient r = 0.86) of both ERR2 and ERR3 by motor neurons with relatively small somas characteristic for gamma motor neurons [15,27,28] and low or negligible levels of the alpha motor neuron marker NeuN [27,28] (NeuN^low^) (Figs 2I, 2I’, S3J, and 3J’). The small-soma ERR2/3^high^ NeuN^low^ motor neurons lacked VGLUT1^+^ varicosities on somatic or dendritic membranes (Fig 2L and 2M), indicating absence of monosynaptic spindle afferent input, a defining characteristic of gamma motor neurons [14,15,27]. Similar to ERR3 [27], ERR2 was initially broadly expressed by most motor neurons during embryonic development (S4A–S4C Fig), but high ERR2 levels became increasingly confined to gamma motor neurons during the first 2 postnatal weeks (S4D–S4R Fig). Consistent with the dependency of gamma motor neuron maintenance on spindle-derived signals [27,28], we observed an absence of ERR2/3^high^ NeuN^low^ motor neurons in adult *Egr3*-deficient mice with impaired muscle spindle development (Figs 2K, S4S–S4X, and S4S’–S4X’). At the same time, we noted that ERR2/3^low^, NeuN^high^ motor neurons were maintained in Egr3-deficient mice (S4S’–S4X’ Fig), while high ERR2/3 levels persisted in a subset of ventral spinal interneurons in both Egr3-deficient mice (S4S’–S4X’ Fig) and mice specifically lacking ERR2/3 in motor neurons (S2O–S2Q Fig). Furthermore, *Imaris* reconstruction studies in control mice showed that, indeed, FG^high^, ERR2/3^high^, NeuN^low^ gamma motor neurons were contacted by few VGLUT1^+^ terminals, while FG^low^, ERR2/3^low^, NeuN^high^ alpha motor neuron somas were covered with VGLUT1^+^ varicosities (Fig 2L–2N). Mouse gamma motor neurons thus exhibit high levels of correlated ERR2 and ERR3 expression. We surmise that previous work using qualitative *Esrrb* mRNA detection by in situ hybridization, which concluded that *Esrrb*/ERR2 was expressed by slow alpha motor neurons [39], was impeded by the inherent inability of these methods to distinguish between the pronounced quantitative differences of *Esrrb*/ERR2 expression levels between gamma and alpha motor neurons and to distinguish between the expression of *Esrrb*/ERR2 by motor neurons and by the interspersed ventral interneurons retained in *Egr3*-deficient mice.

### ERR2/3 are required for the acquisition of a gamma motor neuron biophysical signature

**Fig 3:**
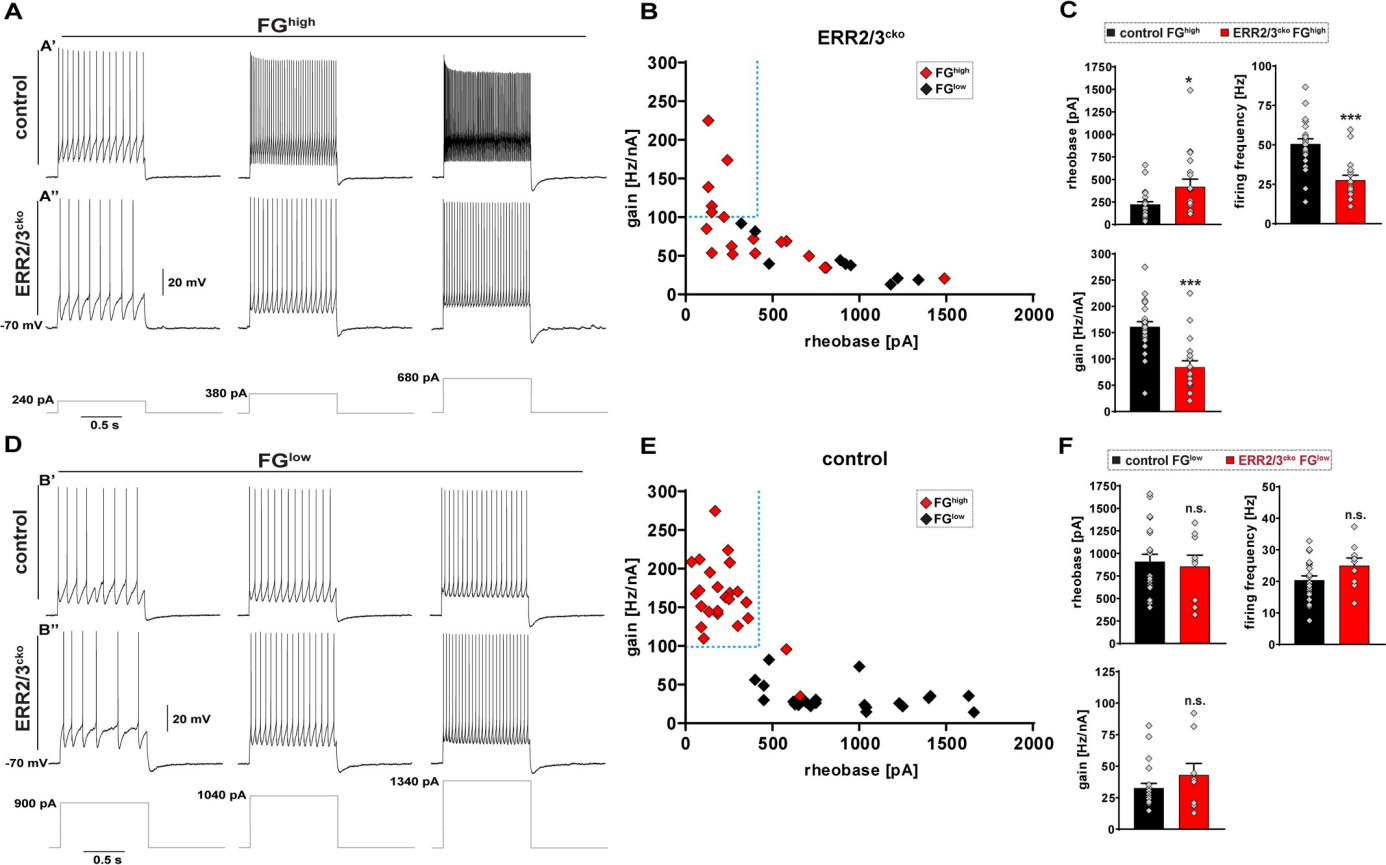
ERR2/3 are required for the acquisition of a gamma motor neuron biophysical signature. **(A)** Example traces of whole-cell patch-clamp recordings upon 240, 380, or 680 pA, 1 second square current pulses from small soma motor neurons exhibiting high levels of FG incorporation (FG^high^) from control mice or ERR2/3^cko^ mice. Control FG^high^ motor neurons **(A’)** exhibit higher firing rates compared to FG^high^ ERR2/3^cko^ motor neurons **(A”)** and gear up their firing rates more rapidly in response to current pulses. **(B)** Scatter plot: lack of segregation of ERR2/3^cko^ FG^high^ gamma motor neuron and ERR2/3^cko^ FG^low^ alpha motor neuron electrophysiological signatures. **(C)** ERR2/3^cko^ FG^high^ motor neurons show higher rheobase (419.72 ± 84.11), lower firing frequency (27.61 ± 3.15), and lower gain (84.01 ± 12.34) compared control FG^high^ motor neuron rheobase (221.87 ± 31.34), firing frequency (50.65 ± 3.23), gain (161.01 ± 9.77), respectively. **(D)** Example traces of whole-cell patch-clamp recordings upon 900, 1,040, or 1,340 pA, 1 second square current pulses from large soma motor neurons with lower levels of FG incorporation (FG^low^) from control mice or ERR2/3^cko^ mice. ERR2/3^cko^ FG^high^ motor neurons exhibit comparable firing rates and properties to control FG^low^ motor neurons. **(E)** Scatter plot: segregation of electrophysiological signatures between control FG^high^ gamma motor neurons versus control FG^low^ alpha motor neurons. (Note: Data are from Fig 1D). **(F)** No significant differences seen in ERR2/3^cko^ FG^low^ alpha motor neuron subtype rheobase (855.55 ± 124.85), firing frequency (25.03 ± 3.76), and gain (43.04 ± 9.81) when compared to control FG^low^ alpha motor neuron subtype rheobase (909.09 ± 82.11), firing frequency (20.41 ± 1.70), gain (32.64 ± 4.02), respectively (see S1 Table for details). Data are presented as mean ± SEM. *n* = # of neurons and *N* = # of mice. Statistically significant differences between FG^high^ and FG^low^ neurons are indicated as *p < 0.05, **p < 0.01, ***p < 0.001, n.s. = not significant, Student *t* test). Data for Fig 3B, 3C, 3E, and 3F can be found in S1 Data.

Due to their correlated expression as well as their molecular similarity, we next asked whether ERR2/3 would contribute to gamma versus alpha motor neuron functional diversification by selectively inactivating both *Esrrb* and *Esrrg* genes in motor neurons via Cre-mediated recombination in cholinergic neurons in *Esrrb^flox/flox^*;*Esrrg^flox/flox^*;*Chat^Cre^* (ERR2/3^cko^) mice (S2O–S2Q Fig). Because *Chat^Cre^*-mediated recombinase activity, which is initiated in early postmitotic motor neurons (S5M–S5T Fig), overlapped with endogenous ERR2/3 expression in motor neurons but not in other cholinergic neuron types throughout the nervous system (S5A–S5L and S6A–S6Z Figs), we concluded that ERR2/3^cko^ mice permitted addressing ERR2/3 function in motor neurons. Since *Esrrb^flox/flox^*;*Chat^Cre.^* and *Esrrg^flox/flox^*;*Chat^Cre^* single-homozygous mice showed only mild impairment of movements, compared to compound ERR2/3-deficient mice (S1–S6 Movies), we reserved further analysis to the ERR2/3^cko^ mice. Whole-cell patch-clamp recordings performed in 20- to 22-days-old ERR2/3^cko^ mice showed that most FG^high^ motor neurons failed to acquire a gamma motor neuron biophysical signature and instead shifted their properties towards a signature resembling those of FG^low^ alpha motor neurons (compare Fig 3B and 3E), with significantly elevated rheobases, as well as lowered gains and firing rates (Figs 3A–3F, S7A, and S6J and S1 Table). Since FG^high^ motor neurons in ERR2/3^cko^ mice showed average firing rates about the same of those for alpha motor neurons (compare Figs 3C, 3F, S7A, and S7F) but still retained somewhat higher gains and lower rheobases (Figs 3C, 3F, and S7B), their overall biophysical signature seemed to have shifted to somewhere between those of slow or fast alpha motor neuron subtypes, thus to some extent resembling yet immature early postnatal gamma motor neurons [30]. At the same time, we did not observe significant changes in the properties of FG^low^ alpha motor neurons in ERR2/3^cko^ mice, when compared to control mice (Figs 3D, 3F, and S7F–S7J and S1 Table), suggesting that the low ERR2/3 levels in alpha motor neurons were not sufficient to significantly influence the acquisition of their characteristic properties. ERR2/3 thus appear to be prerequisite for the acquisition of a gamma motor neuron biophysical signature.

### Development of morphologically distinguishable gamma motor neurons in the absence of ERR2/3

**Fig 4:**
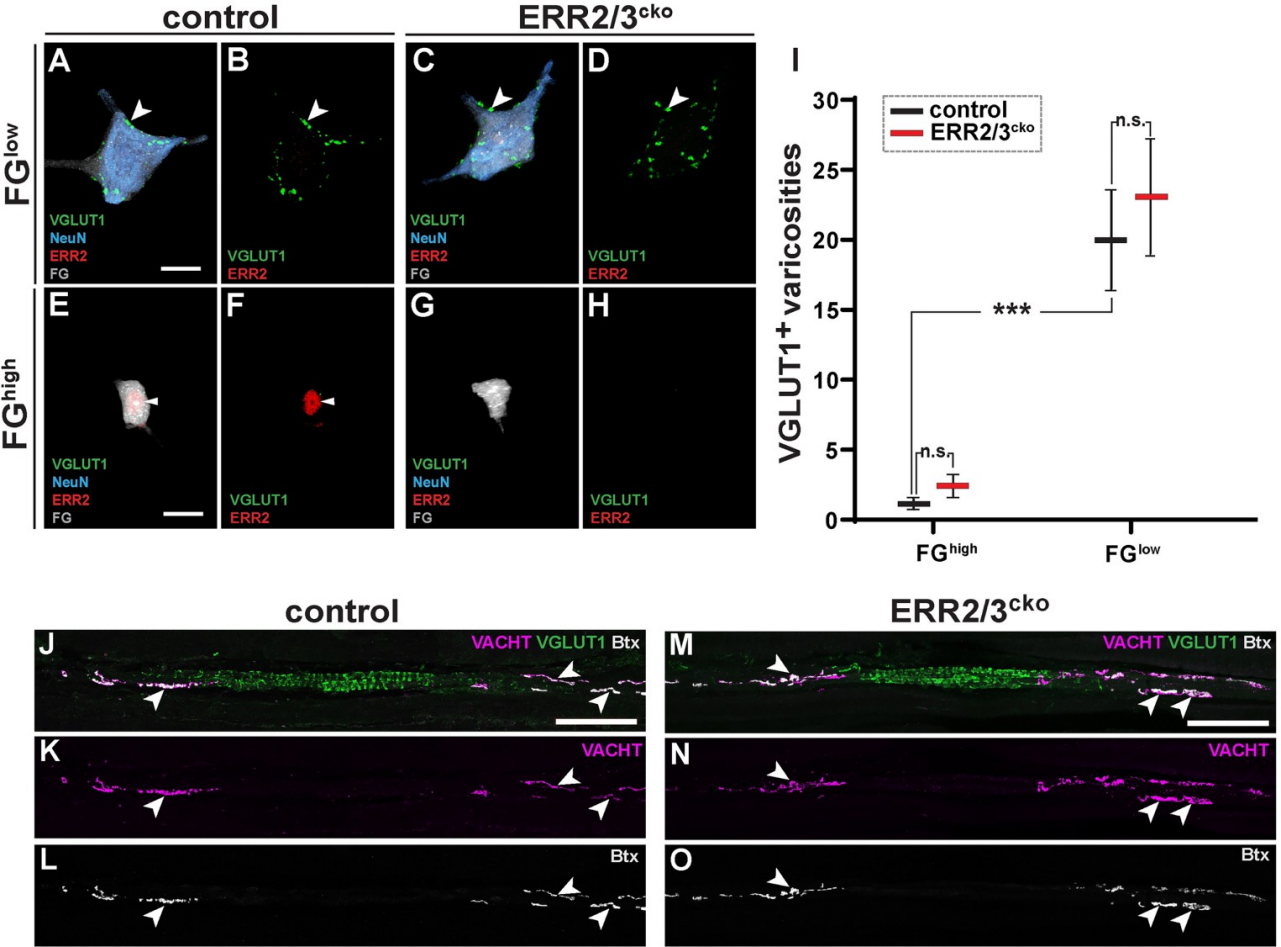
Generation of distinguishable gamma motor neurons in the absence of ERR2/3. **(A-I)** Imaris 3D reconstruction: VGLUT1^+^ synaptic varicosities (green) associated with large FG^low^, NeuN^high^ motor neurons in control **(A, B)** and ERR2/3^cko^ mice **(C, D)** (scale bar: 20 μm). **(E-H)** Absence of VGLUT1^+^ puncta on FG^high^, NeuN^low^ motor neurons in control **(E, F)** and ERR2/3^cko^ mice **(G, H)** (scale bar: 20 μm). **(I)** Significant difference in VGLUT1^+^ varicosities associated with FG^high^, NeuN^low^ and FG^low^, NeuN^high^ motor neurons from control mice. Lack of significant differences in VGLUT1^+^ synaptic varicosities associated FG^low^, NeuN^high^ motor neurons in control (20 ± 3.48, *n* = 9) versus ERR2/3^cko^ mice (23.1 ± 4.06, *n* = 10). Lack of significant differences in VGLUT1^+^ varicosities between control FG^high^, NeuN^low^ (1.1 ± 0.36, *n* = 10) and ERR2/3^cko^ FG^high^, NeuN^low^ motor neurons (2.4 ± 0.78, *n* = 10). **(J-O)** P300 mouse extensor digitorum longus (EDL) muscle spindles of control **(J-L)** and ERR2/3^cko^ **(M-O)** mice. **(J-L)** Distribution of Ia sensory annulospiral endings in the central spindle segment (visualized by VGLUT1, green), motor innervation (VACHT, magenta) and their postsynaptic sites (arrowheads) on intrafusal fibers (BTX, alpha bungarotoxin, grey) (scale bar: 100 μm). **(K-O)** Normal appearance of ERR2/3^cko^ muscle spindles, including Ia sensory annulospiral endings and motor innervation of BTX^+^ postsynaptic sites (arrowheads) on intrafusal fibers (scale bar: 100 μm). Data are presented as mean ± SEM. *n* = # of neurons. Statistically significant differences control and ERR2/3^cko^ motor neurons are indicated as: *p < 0.05, **p < 0.01, ***p < 0.001, n.s. = not significant, Student *t* test). Data for Fig 4I can be found in S2 Data.

Since we observed that loss of ERR2/3 in gamma motor neurons in ERR2/3^cko^ mice led to the loss of a gamma motor neuron biophysical signature, we next asked whether ERR2/3 would also be required for the specification of other aspects of gamma motor neurons, including morphology, marker expression, and synaptic connectivity. In addition to their unique electrophysiological features, gamma motor neurons characteristically possess small soma sizes, lack 1a sensory presynaptic boutons, and innervate muscle spindle intrafusal fibers [13,14,26,27]. Notably, the lack of ERR2/3 in ERR2/3^cko^ mice did not affect the generation of small-soma FG^high^ and NeuN^low^ motor neurons (Fig 4A–4I), consistent with the unaltered input resistance measured in these neurons (S7E Fig). Similar to control mice (Fig 4E, 4F and 4I), the small-soma FG^high^, NeuN^low^ motor neurons in ERR2/3^cko^ mice lacked VGLUT1^+^ varicosities on somatic or dendritic membranes (Fig 4G, 4H and 4I). Moreover, FG^low^ and NeuN^high^ alpha motor neurons retained VGLUT1^+^ terminals in both ERR2/3^cko^ (Fig 4C, 4D and 4I) and control mice (Fig 4A, 4B and 4I). We next investigated whether lack of ERR2/3 in motor neurons would affect the innervation and/or development of muscle spindles. All muscle spindles analyzed in ERR2/3^cko^ mice exhibited overall organization indistinguishable from control mice, including normally formed annulospiral sensory endings in the central segments (Figs 4J–4O and S8A–S8F; *n =* 10 muscle spindles in control, *n* = 8 in ERR2/3^cko^ mice), while 100% of the postsynaptic boutons in the peripheral segments of intrafusal muscle fibers were supplied by motor axon termini in both control and ERR2/3^cko^ mice (arrowheads in Figs 4J–4O and S8A–S8F; *n* = 71 and *n* = 115 intrafusal neuromuscular synapses, respectively). We further found that the expression of the gamma motor neuron molecular marker GFR1A was maintained in the small-soma FG^high^, NeuN^low^ motor neurons in ERR2/3^cko^ mice (S9A–S9H Fig). Moreover, consistent with the absence of detectable impacts of ERR2/3 removal on alpha motor neuron biophysical properties, 100% of the neuromuscular junctions with extrafusal muscle fibers analyzed were supplied by motor axon termini and showed characteristic pretzel-like morphologies in ERR2/3^cko^ mice, indistinguishable from control mice (S8G–S8N Fig; *n =* 51 and *n* = 43 extrafusal neuromuscular junctions, respectively), while expression of the alpha motor neuron markers MMP9 (S9I–S9N Fig) and OPN (S9O–S9T Fig) was retained in ERR2/3^cko^ mice. Taken together, ERR2/3 appear not to be necessary for the specification of morphologically and molecularly distinguishable gamma motor neurons, including important aspects of pre- and postsynaptic connectivity, but subtle impacts on these or other features of gamma motor neuron morphology or connectivity not detectable by the methods used here could not be strictly ruled out. The function of ERR2/3 in gamma motor neurons thus appears to be to a large degree restricted to implementing their characteristic biophysical signature.

### ERR2/3-dependent gamma motor neuron biophysical properties are required for movement accuracy

**Fig 5:**
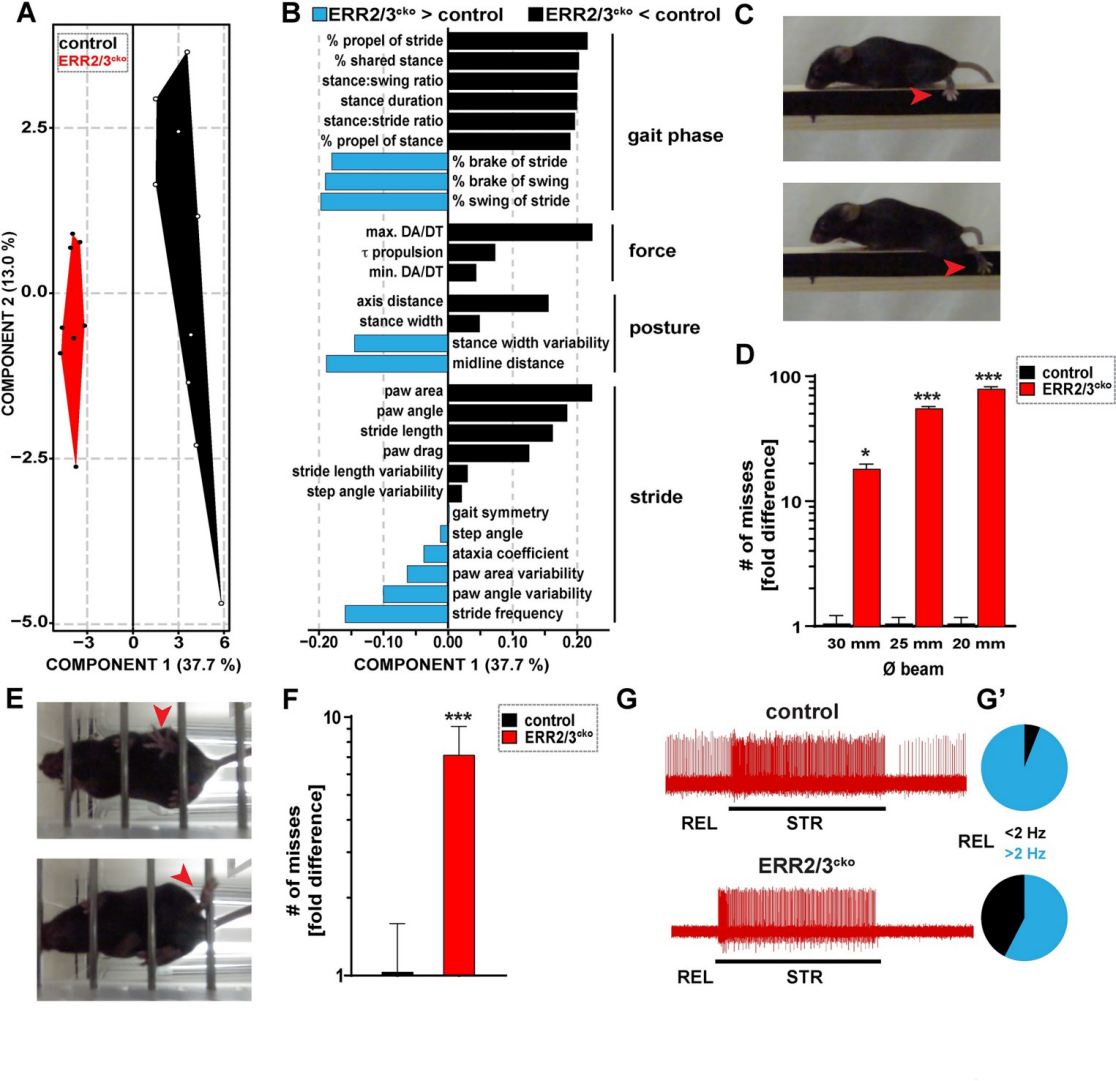
ERR2/3 expressed by gamma motor neurons is required for normal locomotion and precision movements. **(A, B) (A)** Polygon graphs based on partial least squares (PLS) analysis of 58 gait variables measured during treadmill locomotion at 25 m•s^−1^ reveals significant gait alterations in ERR2/3^cko^ mice (*n =* 8, 3 trials/animal) when compared to control mice (*n* = 9, 3 trials/animal). Each dot represents a single animal, polygons group animals of the same genotype, the segregation of which along the x-axis indicate that ERR2/3^cko^ mice exhibit significant gait alterations at all speeds tested (ERR2/3^cko^ mice, 3 trials each). **(B)** Negative (black) or positive (light blue) changes (arbitrary units) in gait variables in ERR2/3^cko^ compared to control mice ranked by predictive value independent of sign, including reduced stance-swing phase ratio (gait phase related), reduced propulsion velocities (force related), decreased stance width (posture related), and increased paw angle variability (stride related). **(C)** Still images of ERR2/3^cko^ mouse navigating a horizontal beam. Red arrow: (example of a “miss”): foot missing the beam during swing-stance transition, causing the hind limb and animal to slip during swing phase (red arrow in **C**). **(D)** ERR2/3^cko^ (18.0 ± 1.75, 55.0 ± 2.20, 79.0 ± 3.49) but not control (1.0 ± 0.175, 1.0 ± 0.125, 1.0 ± 0.125) mice exhibit dramatically increasing erratic locomotion (18-, 55-, and 79-fold increase in the # of misses) upon navigating horizontal beams with decreasing width 30 mm, 25 mm, and 20 mm, respectively (control *N =* 4, ERR2/3^cko^ *N* = 4, 4–5 trials/animal). **(E)** Still images of ERR2/3^cko^ mouse navigating a horizontal ladder. Examples of a “miss” (red arrow in upper panel): foot missing a rung during swing-stance transition, causing the hind limb to slip during swing phase. Note: Animals frequently attempted to compensate such misses by using the “slipped” hind limb to push against the rung and propel itself forward (red arrow in lower panel). **(F)** ERR2/3^cko^ (7.12 ± 2.06) exhibit significantly more Erratic locomotion (7-fold increase in the # of misses) when compared to control (1.0 ± 0.55) upon navigating horizontal ladder (control *N* = 5, ERR2/3^cko^ *N* = 5, 4–5 trials/animal). **(G)** Example traces showing Ia spindle afferent responses during resting length (REL) or stretch (STR) applied by force transducer: normal STR responses, but reduced REL firing of Ia afferents in ERR2/3^cko^ mice (*N* = 10) when compared to control mice (*N* = 10). (**G’**) Pie charts: ratio of Ia afferents firing above or below 2 Hz at REL (total trials: control *n* = 84, ERR2/3^cko^ *n* = 118; control >2 Hz *n* = 79, ERR2/3^cko^ >2 Hz *n* = 68). Data are presented as mean ± SEM. *N* = # of mice, *n* = # of 1a afferents. Statistically significant differences between control and ERR2/3^cko^ mice are indicated as: *p < 0.05, **p < 0.01, ***p < 0.001, n.s. = not significant, Student *t* test). Data for Fig 5A, 5B, 5D, and 5F can be found in S3 Data.

The biophysical properties of gamma motor neurons are thought to be exquisitely suited to effect instant intrafusal fiber peak tension for maintaining and modulating spindle dynamic range and thus muscle proprioception [19]. We therefore asked how a shift towards an alpha motor neuron-like biophysical signature in gamma motor neurons would impact movements relying on proprioceptive feedback from muscles. Because of the exclusive association of high ERR2/3 levels with gamma motor neurons, and the lack of a significant impact of ERR2/3 loss on other motor neuron subtypes, we predicted that beta motor neuron function would be preserved in ERR2/3^cko^ mice, thus allowing us to study the contribution of gamma motor neuron function to movement control in the otherwise intact animal. Similar to Egr3-deficient mice lacking muscle spindles [40,41], ERR2/3^cko^ mice exhibited marked postural and gait alterations (S1–S6 Movies), including changes in metrics related to foot placement, weight bearing, stride, stance, braking and propulsion (Figs 5A, 5B, and S10A–S10E), consistent with the predicted contributions of gamma motor neuron-assisted spindle function to posture, gait phase transitions, and force generation during locomotion [10,15,17,42]. ERR2/3^cko^ mice were nevertheless able to sustain the same range of speeds as control mice in a treadmill locomotion task, suggesting that apart from the perturbance of gamma motor neuron-dependent muscle proprioception, the overall integrity of the skeletomuscular system was preserved (S10A–S10C Fig). However, ERR2/3 loss from motor neurons triggered a failure to handle precision tasks, such as navigating a narrow horizontal beam (Fig 5C and 5D and S7 and S8 Moviess) or a horizontal ladder (Fig 5E and 5F and S9 and S10 Movies), consistent with the particular sensitivity of precision tasks towards perturbations of muscle proprioception [40,41]. These data suggested that biophysical signature implemented by ERR2/3 in gamma motor neurons is prerequisite for effective modulation of muscle spindle-dependent proprioception and, thereby, the execution of precision movements. To further test this idea, we recorded spindle afferent responses via suction electrodes in nerve-muscle preparations [43,44] derived from ERR2/3^cko^ or control mice. In these preparations, muscle stretch applied by a force transducer elicited similar Ia afferent responses in ERR2/3^cko^ and control mice (Figs 5G and S10F–S10I), consistent with the morphologically normal spindle assembly in these animals. In contrast to control spindles (Figs 5G, 5G’, S10F, and S10G), however, ERR2/3^cko^ spindle afferents frequently exhibited reduced firing rates at muscle resting length (Figs 5G, 5G’, S10H, and S10I), possibly due to a decrease in basal intrafusal fiber contractility caused by chronic disruption of gamma motor neuron input. The ERR2/3-dependent implementation of gamma motor neuron biophysical properties therefore appears to be prerequisite for regulating spindle-mediated muscle proprioception and movement control.

### ERR2/3 promote a gamma motor neuron-like biophysical signature in chick

**Fig 6:**
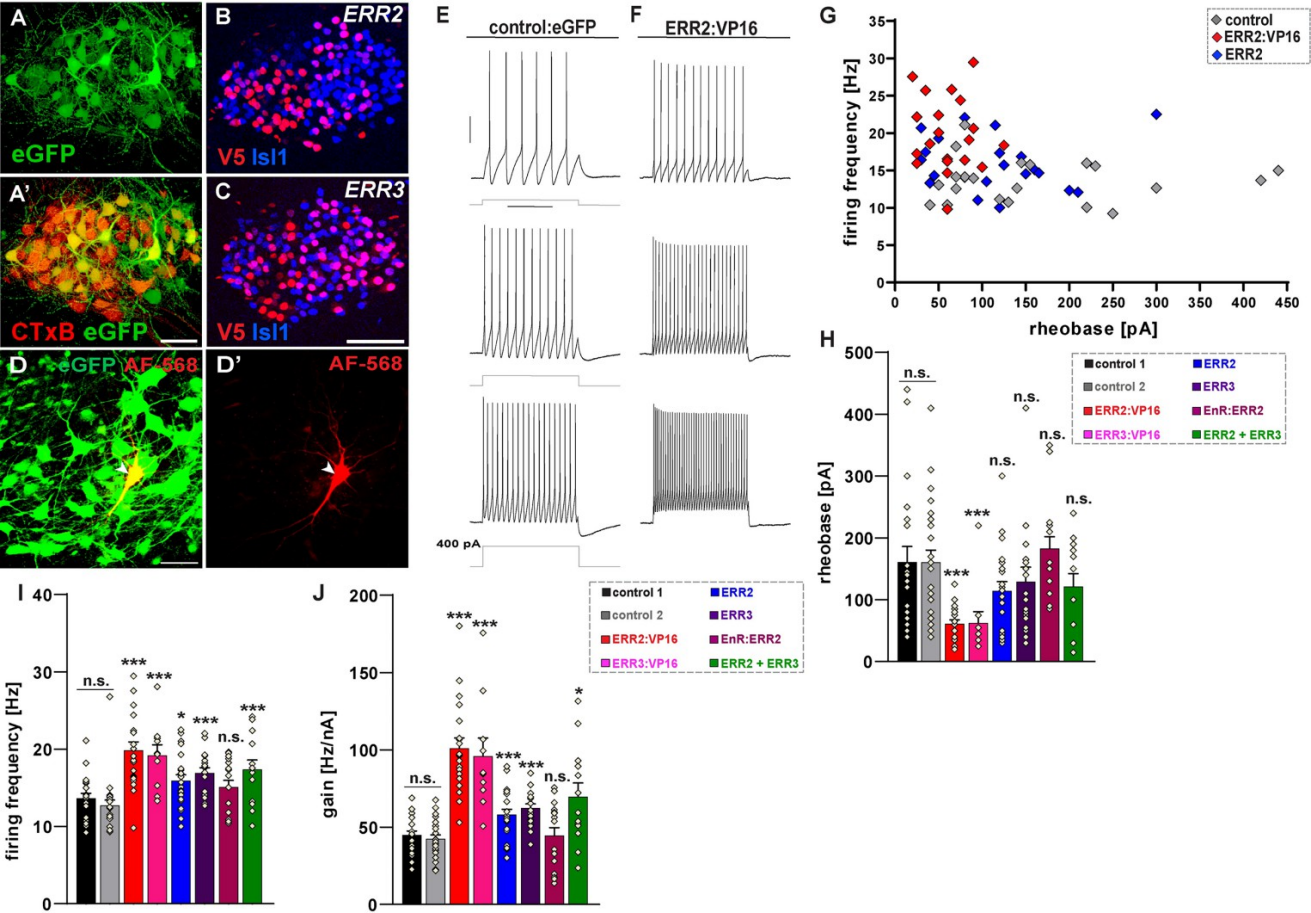
ERR2/3 promote a gamma motor neuron-like biophysical signature in chick through transcriptional activation. **(A-D’)** Overview of E12 chick spinal cord: stable transfection with expression vector driving eGFP expression **(A)** in ventral horn motor neurons. Higher magnification of eGFP expressing neurons further identified as motor neurons by retrograde cholera toxin B (CTxB) tracing upon in ovo injection into the hind limb **(A’)** (scale bar: 100 μm). Examples of expression and nuclear localization of mouse ERR2 **(B)** and ERR3 **(C)** in chick motor neurons (detected via N-terminal V5 epitope tags) (scale bar: 30 μm). eGFP^+^ motor neuron recorded with patch pipette containing Alexa Fluor 568 dye (red) **(D and D’)** (scale bar: 50 μm). **(E, F)** Example traces of current clamp recordings of chick motor neurons (in acute spinal cord slice preparations) forcedly expressing eGFP only (control) **(E)** or ERR2:VP16 and eGFP **(F)**, upon 100, 200, and 400 pA, 1 s square current pulses: motor neurons expressing elevated ERR2 levels exhibit higher firing rates and gear up their firing rates more rapidly in response to current pulses **(F)**. **(G)** Forced ERR2 and ERR2:VP16 expression (*n =* 21, *n* = 20, respectively) shifts chick motor neuron properties towards a gamma motor neuron-like biophysical signature (high firing rates, low rheobases) compared to control (white, *n* = 23). **(H)** Decrease in rheobase upon ERR2, ERR3 (*n* = 21) transfection, yet not significant, but significant decrease in rheobase upon ERR2:VP16 (*n* = 20) or ERR3:VP16 (*n* = 10). No decrease in rheobase upon ERR2:EnR (*n* = 17) transfection. **(I)** Significant increases in gain upon transfection of ERR2, ERR3, ERR2:VP16 or ERR3:VP16, but not upon ERR2:EnR transfection. **(H, I)** Similar decreases in rheobase **(H)**, increase in gain **(I)** upon ERR2+ERR3 (*n* = 13) cotransfection, compared to ERR2 or ERR3 single transfection. No differences detected between control 1 and control 2 with parameters recorded during two different experiments at different time points upon expressing “eGFP only” control vectors, thus demonstrating robustness of the assay. Data are presented as mean ±SEM. *n* = # of neurons. Statistically significant differences are indicated as: *p < 0.05, **p < 0.01, ***p < 0.001, n.s. = not significant, Student *t* test). Data for Fig 6G-J can be found in S4 Data.

We next tested whether ERR2/3 would also be sufficient to promote gamma motor neuron-like biophysical properties by performing whole-cell patch-clamp recordings on chick motor neurons [25] engineered to stably express elevated ERR2 or ERR3 levels (Fig 6A–6D’). We reasoned that if that was the case, ERR2/3 could be able to prematurely push pre-hatching (E12) chick motor neurons, which do not show a distinguishable gamma motor neuron biophysical signature proper (Fig 6G: grey diamonds), towards a signature possibly resembling that of mouse gamma motor neurons. Indeed, forced expression of ERR2 or ERR3 partially shifted motor neuron properties towards a biophysical signature resembling that of mouse or cat gamma motor neurons [19], including a combination of high firing rates and lower rheobases (Fig 6E–6J and S2 Table), although the absolute values of the parameters differed between chick and mouse, likely due to differences in maturation-stage and soma sizes between the pre-hatching chick and postnatal mouse motor neurons analyzed. The effects on motor neuron properties were enhanced by fusing ERR2 or ERR3 to the heterologous transcriptional activation domain VP16 (Fig 6H–6J and S2 Table), but not by fusion to the *engrailed* transcriptional repressor domain (EnR) (Fig 6H–6J and S2 Table), suggesting that in this context, ERR2 and ERR3 primarily function as transcriptional activators. Finally, forced coexpression of ERR2 and ERR3 in chick motor neurons recapitulated the effects on motor neuron biophysical properties by forced expression of either factor alone (Fig 6H–6J and S2 Table), consistent with their apparent mutual redundancy in mouse. Although it remained unclear whether the properties of avian gamma motor neurons would be mediated by the same mechanisms as those in mammals, these results nevertheless suggested the possibility that ERR2/3 could not only be necessary, but also sufficient to promote gamma motor neuron-like functional properties by operating as transcriptional activators.

### ERR2/3 may operate through neural activity modulators, including the shaker K^+^ channel subunit *Kcna10*/Kv1.8

**Fig 7:**
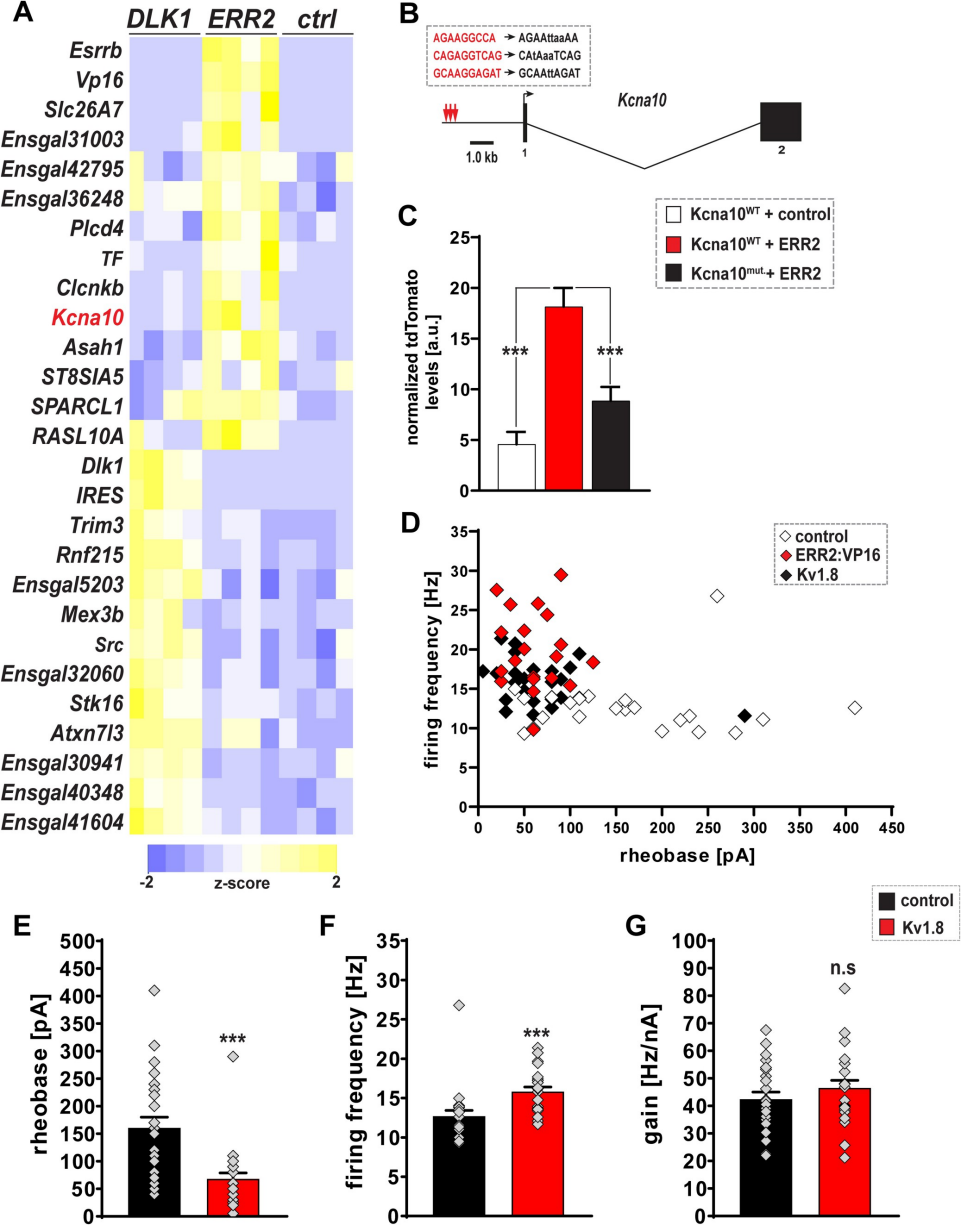
ERR2/3 may in part operate through *Kcna10* to promote a gamma motor neuron-like biophysical signature. **(A)** Heatmap based on transcript reads (transcript per million (TPM)) detected by RNA sequencing: ERR2 (*n =* 4) and Dlk1 (*n* = 4) promote different gene expression signatures in chick motor neurons when compared to control (*n* = 4), including *Kcna10* (red) by ERR2 (only up-regulated genes are shown). **(B)** *Kcna10* genomic locus: 3 clustered ERR2/3 binding sites within promoter region. **(C)** Reporter tdTomato fluorescence driven by wild-type (WT) *Kcna10* promoter is boosted by ERR2 cotransfection (red, *n* = 102) in chick motor neurons when compared to control (gray, *n* = 100) and decrease in tdTomato reporter fluorescence upon mutating the ERR2/3 binding sites (black, *n* = 101). **(D)** Whole-cell patch-clamp recordings: forced ERR2:VP16 expression (red, *n* = 20) shifts chick motor neuron properties towards a gamma motor neuron-like biophysical signature (high firing rates, low rheobases) compared to control (white, *n* = 23). Forced *Kcna10* (black, *n* = 24) expression partially recapitulates the promotion of a gamma motor neuron biophysical signature by ERR2 in chick motor neurons, when compared to control (white, *n* = 23) motor neurons. **(E)** Significant decreases in rheobase upon *Kcna10* (*n* = 24) transfection. **(F)** Significant increase in firing frequency upon *Kcna10* (*n* = 24) transfection. **(G)** No significant increase in gain upon *Kcna10* (*n* = 24) transfection. Data are presented as mean ± SEM, *n* = number of experiments or neurons. Statistically significant differences are indicated as: *p < 0.05, **p < 0.01, ***p < 0.001, n.s. = not significant, Student *t* test). Data for Fig 7A and 7C can be found in S5 Data and data for Fig 7D–7G can be found in S2 Data. The complete set of values for the RNAseq experiments can be found in S6 Data.

**Fig 8:**
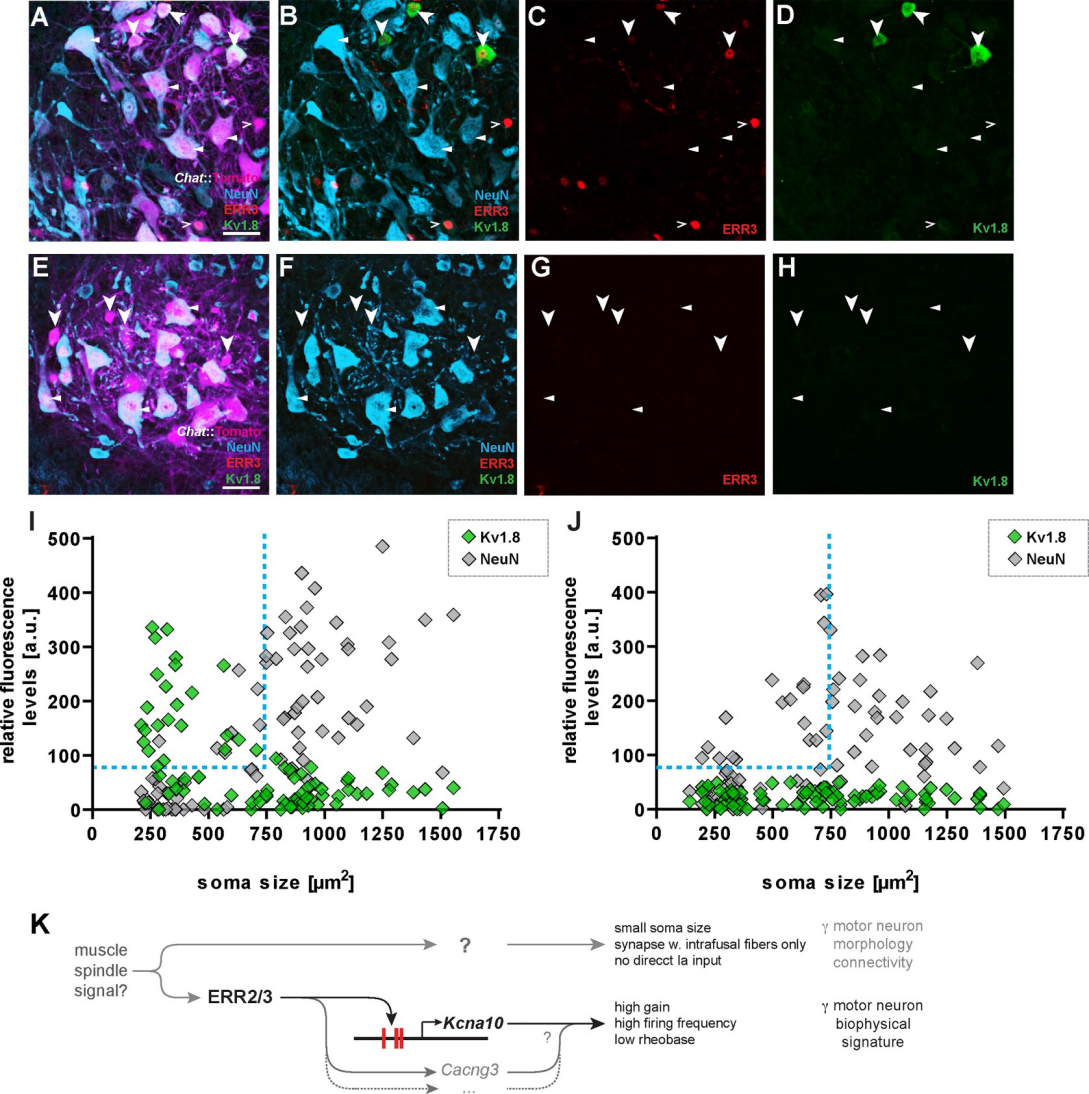
ERR2/3-dependent expression of Kv1.8 (protein product of *Kcna10*) in gamma motor neurons in mouse. **(A-D)**. Transversal section of adult (mouse lumbar spinal cord ventral horn (scale bar: 50 μm)). Bold arrowheads: high Kv1.8 levels in small ERR3^high^ NeuN^low^ tdTomato^+^ motor neurons. Triangles: low or not-detectable Kv1.8 levels in ERR3^low^ NeuN^high^ tdTomato^+^ motor neurons. Open arrowheads: some ERR3^high^ NeuN^low^ tdTomato^+^ motor neurons possess low or not-detectable Kv1.8 levels. **(E-H)** Loss of detectable Kv1.8 levels in NeuN^low^ tdTomato^+^ motor neurons (arrowheads) in ERR2/3^cko^ mice. **(I)** Two-dimensional scatter plot of relative fluorescence intensities over soma sizes: high Kv1.8 levels in NeuN^low^ motor neurons with small somas and low Kv1.8 levels in NeuN^high^ motor neurons with larger somas in control mice (*n* = 84, *N =* 3). **(J)** Loss of high Kv1.8 levels in NeuN^low^ motor neurons with smaller somas in ERR2/3^cko^ mice (*n* = 84, *N* = 4). **(K)** Summary: ERR2/3 promote a gamma motor neuron biophysical signature required for spindle-dependent proprioceptive movement control, possibly by operating through the activation of neural activity modulator genes including *Kcna10* (and likely others), while the generation of morphologically distinguishable gamma motor neurons proper may involve other factors operating earlier in development. *n*: number of neurons, *N*: number of mice. Data for Fig 8I and 8J can be found in S2 Data.

To identify through which intermediate factors ERR2/3 would operate to promote gamma motor neuron-like electrophysiological properties in chick, we performed comparative transcriptome profiling by RNA sequencing of chick motor neurons forcedly expressing ERR2 (Figs 7A and S11A). In these experiments, elevated ERR2 levels significantly activated a set of genes largely distinct from the gene signature activated by the previously identified (fast) alpha motor neuron determinant Dlk1 [25] (Figs 7A, S11A, and S11B and S3 Table). The set of genes whose expression was altered by ERR2 expression were enriched in potential neural activity modulators (S11C and S11D Fig), including *Kcna10*, which encodes Kv1.8, a member of the shaker family of voltage-gated K^+^ channels implicated in neuronal excitability [45] (Figs 7A and S11B). We found that the promoter region of the *Kcna10* genomic locus contained an evolutionary conserved region (ECR) with 3 clustered ERR2/3 DNA binding motifs (Fig 7B) that were conserved between mouse and chick (S11E Fig). In chick motor neurons, moreover, ERR2 boosted reporter gene activity driven by the *Kcna10* ECR (Figs 7C and S11F–S11K), but not upon introducing mutations into the ECR’s ERR2/3 binding motifs (Figs 7B, 7C, S11N, and S11K). Ultimately, forced expression of *Kcna10* shifted motor neuron electrophysiological properties in a manner partially recapitulating those elicited by ERR2/3, including lower rheobases and higher firing rates (Fig 7D–7H and S2 Table). However, not all parameters associated with murine gamma motor neurons changed significantly (Fig 7G and S2 Table), suggesting that in chick, ERR2 would operate through additional neural activity modulators (S3 Table). While small motor neurons in birds express NKAα3 [46], a marker for gamma motor neurons in mouse [31], and high levels of *Esrrb* and *Esrrg* are expressed by a subset of motor neurons in late-gestation chick in a pattern resembling that previously observed for *Esrrb* in mouse motor neurons [39] (S11L–S11O Fig), the extent to which the mechanisms underlying gamma motor neuron specification or function would be conserved between avian and mammals, if at all, remained unclear. Intriguingly, we found Kv1.8 to be selectively expressed by small soma-size NeuN^low^ gamma motor neurons in mouse (Fig 8A–8D and 8I). Moreover, the expression of Kv1.8 in small soma-size NeuN^low^ motor neurons was abolished in ERR2/3^cko^ mice (Fig 8E–8H and 8J), thus neatly complementing the observed up-regulation of *Kcna10* upon ERR2 overexpression in chick. Taken together, the similarity of the biophysical signatures promoted by ERR2/3 in chick motor neurons with the ERR2/3-dependent gamma motor neuron biophysical signature in mouse, the presence of conserved clustered binding sites for ERR2/3 in the promotor region of both mouse and chick *Kcna10* genomic loci, the promotion of *Kcna10* expression by ERR2/3 in chick depending on these binding sites, and the ERR2/3-dependent expression of Kv1.8 in gamma motor neurons in mouse suggest that ERR2/3 operate as conserved transcriptional activators, by implementing a gene expression program encoding neural activity modulators, to promote a gamma motor neuron biophysical signature. However, despite compelling similarities, the extent to which chick and mouse gamma motor neurons and the mechanisms underlying their specification or properties would be comparable remains to be determined.

## Discussion

The contribution of gamma motor neurons to proprioceptive movement control in mammals has long been established [8–10], but despite recent progress in assigning markers and gene expression signatures to these neurons [27–34], how they are specified and/or acquire their unique properties remained unaddressed. In the present study, we identified ERR2/3 as determinants of the divergence of gamma and alpha motor neuron biophysical properties, which may involve the transcriptional activation of genes encoding neural activity modulators (Fig 7K). The ERR2/3-dependent establishment of a gamma motor neuron biophysical signature, in turn, appears to be necessary for regulating muscle spindle-dependent muscle proprioception underlying movement accuracy.

### Gamma motor neuron generation and maturation

Previous results suggest that between P0 and P6, the biophysical properties gamma motor neurons are yet immature and lie in between those of slow and fast alpha motor neurons [30], thus somewhat resembling the properties of ERR2/3-deficient gamma motor neurons. Therefore, while gamma motor neurons proper are generated before birth [29], and slow and fast alpha motor neuron properties are already distinguishable by P7 [25], a distinctive gamma motor neuron biophysical signature emerges sometime between P7 [30] and P20. This period may therefore approximate the time window of gamma motor neuron maturation instigated by ERR2/3, raising the question whether their actions could be superimposed on an initial alpha motor neuron-like groundstate [25,26]. Two observations suggest that the latter is not the case. First, the gamma motor neuron biophysical signature is quite distinct from those of the alpha motor neuron subtypes, for instance, combining high gains and firing rates with low rheobases, in contrast to the combination of either low gains/firing rates with intermediate rheobases (slow alpha motor neurons) or intermediate gains/firing rates with high rheobases (fast alpha motor neurons) [24,25], which would be inconsistent with a purely additive effect of ERR2/3 on immature (alpha motor neuron-like) gamma motor neuron properties. Second, we observe that not only activation, but also (likely indirect) repression of potential neural activity modulator genes upon forced ERR2/3 expression in chick (S3 Table), suggesting that ERR2/3 may actually reconfigure the combination of such genes, including voltage-gated K^+^ and Ca^2+^ channel subunits, expressed by gamma motor neurons. About the significance of the expression of ERR2/3 by most motor neurons before birth, we can at present only speculate. It is conceivable, however, that ERR2/3 actions are delayed by the postnatal activation of yet to be identified cofactors (possibly by muscle spindle-derived signals), critical for the ability of ERR2/3 to activate genes that together promote mature gamma motor neuron biophysical properties.

### Gamma motor neuron subtypes and beta motor neurons

Two types of gamma motor neuron output, static or dynamic, are thought to normally tune muscle spindle sensitivity during different aspects of movements [10,16,47]. Three observations led us to conclude that the actions of ERR2/3 do not distinguish between both types of gamma motor neurons. First, we found ERR2/3 to be expressed by all gamma motor neurons. Second, consistently, gamma motor neuron biophysical properties appeared to be disrupted as a whole upon loss of ERR2/3. Third, the range of movements affected by ERR2/3 removal from motor neurons (from basic locomotion to precision movements) further suggest that ERR2/3 function would be required for both static and dynamic modulation of spindle function. The mechanistic bases underlying the two different outputs of gamma motor neurons to spindles thus remain to be addressed. We noted that Kv1.8 was not expressed by all gamma motor neurons, which may provide a starting point for investigating the basis for the distinction between the two predicted gamma motor neuron subtypes. How about the beta motor neurons? Beta motor neurons connect to both extrafusal and intrafusal muscle fibers but are thought to be otherwise morphologically indistinguishable from alpha motor neurons [48–51]. Since beta motor neurons, like the alpha motor neurons, possess relatively large soma sizes and receive monosynaptic Ia afferent input [50,51], we were able to rule out that ERR2/3 were also operating in beta motor neurons. Since beta motor neurons so far have mostly been studied in the cat and have been identified solely based on their simultaneous innervation of intrafusal and extrafusal fibers [51], both the abundance of beta motor neurons and their significance for movement control in mouse awaits further study. However, the identification by single-cell transcriptome profiling of a motor neuron gene expression signature distinct from those of gamma and alpha motor neurons could aid this quest [33].

### Gamma motor neuron gene expression signatures

Apart from *Esrrb/g* proper, we observed little, but notable overlap between the genes activated by ERR2/3 in chick motor neurons and previously published single-cell RNAseq data linked to murine gamma motor neurons [32–34] (S3 Table). One notable example was *Cacng3* [34], the protein product of which can be incorporated with that of *Cacna1c*, also linked to mouse gamma motor neurons [34], into the same L-type Ca^2+^ holochannels [34]. Similarly, while *Kcna10*/Kv1.8 did not show up in any of the single-cell data sets [32–34], one of them contained *Kcna5* [32], a related shaker channel subunit gene. The limited overlap between the chick and mouse data sets likely had three reasons. First, despite their obvious power for revealing the cell type composition of organs and tissues, these single-cell approaches inherently capture only part of a cell’s transcriptome [52]. This is exemplified by the absence of *Kcna10*/Kv1.8 and other gamma motor neuron markers identified in mouse (for instance, *Atp1a3*/NKAα3 [46] and *Wnt7a* [29]) from the independent single-cell transcriptome data sets, as well as the relatively limited overlap between these data sets proper [32–34]. Second, ERR2/3 likely targets only a fraction of gamma motor neuron genes, while the list of potential target genes we identified in chick is unlikely to be complete. This may have also been because the chick motor neurons were analyzed at stages at which they were not yet mature, which, in turn, could have resulted in insufficient chromatin accessibility of ERR2/3 target sites and may have also contributed to the paradoxical down-regulation by ERR2 of some genes associated with mouse gamma motor neurons, including *Grin2a* [34] and *Ret* [32] (S3 Table). Third, there may be species-specific differences in the genes targeted by ERR2/3 and/or low levels of conservation of the corresponding genes/proteins leading to differences in annotation, which together can obscure actual relationships. An example of this is *ENSGALG00000015953* (S3 Table), encoding a protein with similarity to Serotonin receptor genes linked to mouse gamma motor neurons [34]. Because of these limitations, it will be interesting to apply single-cell transcriptomics to mouse motor neurons lacking ERR2/3 to more directly appraise the set of genes through which these factors likely promote gamma motor neuron properties in mouse.

### Gamma motor neurons of birds and mammals

Whether the presence of gamma motor neurons in both birds and mammals reflect a common phylogenetic origin of these neurons or rather convergent adaptations remains unclear [53]. On the one hand, the observation that subsets of chick motor neurons share molecular markers with mouse gamma motor neurons [46], including *Esrrb/g*, together with the regulation of *Kcna10* and other genes linked to mouse gamma motor neurons by ERR2/3 in chick [2–34], could point to a common phylogenetic origin of avian and mammalian gamma motor neurons and the mechanisms underlying their specification. On the other hand, it is also conceivable that a preexisting ERR2/3-dependent gene regulatory program was adopted independently in the avian and mammalian lineages to promote similar neuron subtype-specific properties, which, in turn, contributed in parallel to improved motor control by facilitating alpha motor neuron-independent regulation of muscle prorioception [53]. A more fine-grained comparison of motor neuron gene expression between avian and mammalian motor neurons, as well as extant vertebrates with more ancestral spinal motor systems, could shed more light on the possible phylogenetic relationships and origins of these neuron types.

### Outlook

In addition to gamma motor neurons, ERR2/3 are expressed by oculomotor neurons (S5G–S5L Fig), as well as by subsets of noncholinergic neurons in several areas of the brain (S6 Fig). It will therefore be interesting to test whether either or both factors would serve similar roles in promoting biophysical properties in other neuron types and what impacts these actions would have on the function of the neural circuits in which these neurons are embedded.

## Experimental procedures

### Mouse models

Mouse husbandry and experiments involving mice were approved by and conformed University, state, federal, and European Union animal welfare regulations. For experiments using wild-type mice, C57BL/6J and CD1 strains (both purchased from Charles River Laboratories, Wilmington, USA) were used. “*Chat*::tdTomato” mice were generated by interbreeding mice carrying “*Rosa26^floxtdTomato^*” [54] and “*Chat^Cre^*” [55] targeted alleles (both purchased from Jackson Laboratories, Bar Harbor, USA). Egr3^ko/ko^ mice [56] were a gift from Warren G. Tourtellotte (Northwestern University, currently Cedars Sinai Medical Center). ERR2/3^cko^ (*Esrrb^flox/flox^*; *Esrrg^flox/flox^*; *Chat^Cre^*) mice were obtained by interbreeding mice carrying *Chat^Cre^* and floxed *Esrrb^loxp/loxp^* (exon 2 of *Esrrb* gene (chromosome 12) has been flanked by two loxP sites) [57] alleles with mice carrying floxed *Esrrg^loxp/loxp^* (in which exon 2 of *Essrg* gene (chromosome 1) has been flanked by two *loxP* sites alleles and were derived from the ES cell repository of the Institut Clinique de la Souris (ICS), Alsace, France). Unless otherwise indicated, “controls” for comparison with ERR2/3^cko^ mice were of the genotype *Esrrb^flox/flox^*; *Esrrg^flox/flox^* (unrecombined alleles) and Egr3^ko/+^ or C57bl6/J (wild type) mice for comparison with Egr3^ko/ko^ mice.

### Immunodetection

Approximately 30 to 60 μm frozen OCT (Sakura Finetek GmbH, Umkirch, Germany) sections were incubated overnight in PBS containing 1% BSA and 0.5% Triton-X 100 as described [58–60], using the following antibodies: mouse anti-NeuN (1:1,500, Cat. # MAB377, Merck KGaA, Darmstadt, Germany), chick anti-GFP (1:2,000, Cat. # ab13970, Abcam plc., Cambridge, UK), rabbit anti-dsRed (1:1,000, Cat. # 632496, Takara Bio Europe SAS, Saint-Germain-en-Laye, France), mouse anti-ERR2 IgG2b (1:4,000, Cat. # PP-H6705-00, R&D Systems, Minneapolis, USA), mouse anti-ERR3 IgG2a (1:1,000, Cat. # PP-H6812-00, R&D Systems, Minneapolis, USA), guinea pig anti-VACHT (1:1,000, Cat. # 139105, Synaptic Systems GmbH, Göttingen, Germany), rabbit anti-VACHT (1:750, Cat. # 139103, Synaptic Systems GmbH, Göttingen, Germany), guinea pig anti-VGLUT1 (1:1,000, Cat. # AB5905, Merck KGaA, Darmstadt, Germany), rabbit anti-Isl1/2 (1:2,500, Gift from S. L. Pfaff, Salk Institute La Jolla, CA, USA), mouse anti-V5 (1:1,000, Cat. # 37–7500, Thermo Fisher Scientific, Waltham, USA), rabbit anti-KCNA10 (1:500, Cat.# Rb1656-211110-WS, Osenses, Keswick Australia), goat anti-GFRA1 (1:500, Cat.# AF560, R&D Systems, Minneapolis, USA), mouse anti-OPN (1:200, Cat.# sc-21742, CA, USA), goat anti-MMP9 (1:2,000, Cat.# M9570, Merck KGaA, Darmstadt, Germany), goat anti-CHAT (1:100, Cat.#AB144P, Merck KGaA, Darmstadt, Germany). Secondary detection of anti-ERR2 and anti-ERR3 IgG isoforms was accomplished by Alexa Fluor-conjugated anti-mouse IgG2a (1:2,000, Cat. # A-21131, Thermo Fisher Scientific, Waltham, USA) or IgG2b (1:2,000, Cat. # A-21147, Thermo Fisher Scientific, Waltham, USA) antibodies.

### Microscopy, image analysis, and quantification

Fluorescence microscopy was performed using Zeiss LSM 710 and LSM 800 laser scanning microscopes. Approximately 6 to 28 optical sections were obtained at a step-size of 0.8 to 1.5 μm. Care was applied to avoid oversaturation and distortion of relative expression levels during image acquisition. Raw images were imported into ImageJ and z projected at maximum intensity. For quantification of expression levels, raw pixel intensities were quantified in individually outlined motor neuron nuclei “region of interests” (ROIs) using Adobe Photoshop CS5.1. using background levels and the neuron with the highest fluorescent intensity for normalization. For quantification of VGLUT1^+^ varicosities, a step-size of 0.80 μm was used to obtain an average of 20 optical sections. Raw Z-stack Carl Zeiss files (.czi) were imported into *Imaris 8*.*0* (Bitplane AG, Zurich, Switzerland). Neuronal surfaces were rendered to detect VGLUT1 varicosities (“spots”) on motor neuron somas and dendrites using the “find spots close to surface” function [61] and guided by specific parameters.

### Electrophysiology of gamma versus alpha/beta motor neurons in mice

Mice (P20-22) were intraperitoneally injected with 0.5% to 2% (w/v) FG (Flurochrome, Denver, CO) dissolved in PBS (pH = 7.2) at a volume of 0.10 ml/10 g body weight. The animals (1-day post-FG injection) were intraperitoneal injected with 100 mg/kg body weight of Ketamine, 20 mg/kg body weight Xylazine in PBS (pH = 7.2) at a volume of 0.10 ml/10 g of body weight. After losing their righting reflex, they were placed on a bed of ice until loss of toe pinch response. Immediately after, the animals were decapitated and quickly eviscerated. The torso was placed in chilled Dissecting aCSF (DaCSF) solution (in mM): 191 sucrose, 0.75 K-gluconate, 1.25 KH_2_PO_4_, 26 choline bicarbonate (80% solution), 4 MgSO_4_, 1 CaCl_2_, 20 dextrose, 2 kynurenic acid, 1 (+)-sodium L-ascorbate, 5 ethyl pyruvate, 3 myo-Inositol. The solution was maintained at pH 7.3 using carbogen (95% O_2_−5% CO_2_), and osmolarity was adjusted to approximately 305 to 315 mOsm with sucrose. Vertebrectomy was performed to extract the spinal cord. Ventral roots were cut and the meninges were removed from the spinal cord. The thoracolumbar region (T10-L5) of the spinal cord was isolated and embedded in agar block (4% agar in Recording aCSF (RaCSF)) using 20% gelatin in RaCSF). Slices (370 μm) were obtained using Leica VT1200 S (Leica Biosystems, GmbH, Nussloch Germany). The slices were incubated in 35°C RaCSF for 30 minutes and 30 minutes at room temperature before the recordings. Motor neurons (MNs) were recorded in the RaCSF solution (mM): 121 NaCl, 3 KCl, 1.25 NaH_2_PO_4_, 25 NaHCO_3_, 1.1 MgCl_2_, 2.2 CaCl_2,_ 15 dextrose, 1 (+)-sodium L-ascorbate, 5 ethyl pyruvate, 3 *myo*-Inositol. The solution was maintained at pH 7.4 using carbogen (95% O_2_−5% CO_2_), and osmolarity was adjusted to approximately 305 to 315 mOsm with sucrose. Whole-cell patch-clamp recordings were performed from FG-labeled motor neurons in the ventral horn from control and ERR2/3^cko^ animals. FG^high^ and FG^low^ MNs were visually identified using Olympus BX51W1 microscope (Olympus Europa SE & Co. KG, Hamburg, Germany) equipped with an FG longpass filter set (350 nm bandpass and 425 nm longpass filter) (AHF analysentechnik AG, Tübingen Germany). The patch pipette (resistances of 3 to 6 MΩ) was filled with intracellular solution (mM): 131 K-methanesulfonate (or MeSO_3_H), 6 NaCl, 0.1 CaCl_2_, 1.1 EGTA-KOH, 10 HEPES, 0.3 MgCl_2_, 3 ATP-Mg^2+^ salt, 0.5 GTP-Na^+^ salt, 2.5 L-glutathione reduced, 5 phosphocreatine di(tris) salt. The solution pH was adjusted to 7.25 with KOH, and the osmolarity was adjusted to 300 mOsm using sucrose. Data analysis was performed offline using Axograph X Version 1.6. Previously established protocols were applied to obtain membrane properties of rheobase, input resistance, capacitance, *F-I* curve, AHP amplitude, half-width and half-decay times [25,62–66]. For obtaining the *F-I* curve for discharge properties, spikes were elicited by applying 20 pA, 1,000 ms square current pulses to cells. Currents up to 1 nA were injected for all neurons. For the mouse recordings, currents of up to 1 nA were injected into FG^high^ and 3 nA into FG^low^ MNs from control and ERR2/3^cko^ spinal cord slices. The firing frequency (Hz) was defined as the inverse of the duration between first two spikes (instantaneous firing frequency), or 0.25 to 0.75 seconds of the current pulse (steady-state firing frequency), or 1 second current pulse (mean firing frequency). The gain (Hz/nA) was defined as the slope of the regression line of mean firing frequency upon current injection [65].

### Gait analysis

Control (*n* = 9) and ERR2/3^cko^ (*n =* 8) mouse locomotion on a treadmill was recorded through automated high-speed motion capture (DigiGait, Mouse Specifics, Framingham, USA) as described previously [25,67]. This method generates over 50 different gait variables, which exceed the number of observations (8 to 9 animals per genotype) and are partially redundant. We therefore used the Partial Least Squares (PLS) regression, which is optimized for predictive modeling of multivariate data and to deal with multicollinearity among variables. Orthogonal Signal Correction PLS (OSC-PLS) was used as an extension of PLS to separate continuous variable data into predictive and uncorrelated information for improved diagnostics as well as more easily interpreted visualization. The method seeks to maximize the explained variance between-groups in a single dimension and separates the within-group variance (orthogonal to classification goal) into orthogonal dimensions or components. We modified an existing R script [68,69], originally designed for chemometrics analysis [70], to adapt it to our behavior data. The OSC-PLS method was applied to the complete centered-scaled data set in order to define a model. A model with an optimal number of 2 components was used for subsequent analysis in both fore and hind limbs. Our model coefficient of determination Q2, i.e., the model’s fit to the training data, and its Root Mean Square Error of Prediction (RMSEP), i.e., the model’s predictive ability on the testing data were calculated using the Leave-One-Out method [68]. The model was finally validated to ensure that it was performing better than a random model, while not being overfitted. We conducted an internal cross-validation by performing permutations in the original data, from which 2/3 was used to fit a model. This model was then used to predict group memberships on the remaining 1/3 testing set. The process was repeated 100 times, and Q2 and RMSEP values were averaged over the repeats. We finally compared our model’s Q2 and RMSEP values to the mean Q2 and mean RMSEP values of the permuted models. The results of the two-sample Student *t* tests used for the comparisons indicated a probability much inferior to 0.1% of achieving a performance similar to our model by random chance.

### Precision movement tasks

Precision movements were successively tested using a custom-built setup with a 100-cm horizontal beam of 20, 25, and 30 mm width, respectively [71]. Age-matched control (*n* = 4) and ERR2/3^cko^ (*n* = 4) 10-month-old female mice were trained to move across the beam into a home cage at its end. The animals were trained for 3 days (4 trials/animal, bidirectional on the beam) and tested on the fourth day (4 to 5 trials/animal, bidirectional on the beam). Furthermore, a custom-built setup to record skilled locomotion on a 100-cm horizontal ladder, with 3 mm rungs spaced at 14 mm (similar to a setup previously described) [43,44] was used to test age-matched 8-weeks-old female control (*n* = 5) and ERR2/3^cko^ (*n* = 5) mice. Mice were trained to move across the ladder into a home cage placed at its end and were trained for 2 days (3 trials/animal, single direction on the ladder) for 2 weeks and tested on the third day of the second week (4 to 5 trials/animal, single direction on the ladder). The animals were rested in their home cage for 1 minute between trials for both tasks. Animal locomotion was recorded using GoPro HD Hero2 (GoPro, San Mateo, USA) fitted to a custom-built slider track. The videos were acquired at 120 fps at an image size of 848 × 480 and stored as MP4 files. The videos were processed using GoPro Studio Version (2.5.4) and proDAD Defishr Version 1.0 (proDAD GmbH, Immendingen, Germany). The figure videos were slowed to 25% to 40% of original speed and reduced to 60 fps using GoPro Studio Version (2.5.4). The fish-eye view was removed from videos using proDAD Defishr Version 1.0 (proDAD GmbH, Immendingen, Germany) with Mobius A Wide preset and Zoom adjusted to 110.0 or 180.0. A “miss” was scored when the mouse paw failed to locate the rung or the beam leading to the animal to slip or to halt until the paw regained its footing. An average of approximately 40 steps per trial were analyzed for each mouse.

### Muscle spindle afferent recordings

Ex vivo extensor digitorum longus (EDL) muscle-nerve preparations were used to study the response of muscle sensory neurons to stretch and was essentially performed as described [43]. Briefly, the dissection of muscle (with the nerve attached) was performed in low calcium and high magnesium solution [43,72]. Then, the muscle was placed in recording solution 22 to 24°C and equilibrated with carbogen and was then hooked to a dual force and length controller-transducer (300C-LR, Aurora Scientific, Aurora, Canada) with the help of 5–0 sutures tied to its tendons. Following the determination of resting length (Lo) as described in previous studies, a suction electrode (tip diameter 50 to 80 μm) connected to an extracellular amplifier (EXT-02F, npi Electronics GmbH, Tamm, Germany) was used to sample muscle spindle afferent activity. Data acquisition was performed with LabChart 8 connected to PowerLab 8/35 (ADInstruments, Oxford, UK). Afferent activity, when obtained was checked for the presence of a characteristic pause following a series of 30 twitch contractions at 1 Hz and if the pause was present, the unit was identified as a muscle spindle afferent. Then a series of 9 ramp and hold stretches were delivered to the muscle (2.5% Lo, 5% Lo, and 7.5% Lo at 40% Lo/second, protocols were kindly provided by K.A. Wilkinson lab). The data were recorded and analyzed offline with a custom written MATLAB code. Spikes were detected using KMEANS [2]. For each afferent, resting discharge (RD), dynamic peak discharge (DP), dynamic index (DI), and static response (SR) were calculated. A total of 10 control and 10 ERR2/3^cko^ animals were used for recordings, from which 8 control and 9 ERR2/3^cko^ spindle afferents were analyzed.

### Molecular cloning

Mouse *Esrrb* (NM_011934.4) (ERR2) and Mouse *Esrrg* (NM_011935.3) (ERR3) open reading frames were isolated using PrimeScript 1st cDNA synthesis Kit Takara Bio Europe SAS, Saint-Germain-en-Laye, France) following manufacturer’s directions from E18.5 mouse spinal cord total RNA and cloned after PCR amplification with the following oligonucleotide primers:

*Esrrb* Forward 5′-CATGCCATGGATGTCGTCCGAAGACAGGCACC-3′,

*Esrrb* Reverse 5′-CATGCCATGGCACCTTGGCCTCCAGCATCTCCAGG-3′,

*Esrrg* Forward 5′- CATGCCATGGATGGATTCGGTAGAACTTTGC-3′,

*Esrrg* Reverse 5′-CATGCCATGGGACCTTGGCCTCCAGCATTTCC-3′.

The chick *Kcna10* (NP_989793) open reading frame was isolated from E5.5 chick embryo total RNA as above using the following primers:

*Kcna10* Forward 5′-ATGATGGACGTGTCCAGTTGG-3′.

*Kcna10* Reverse 5′-TTTTTTGGCCTTGTCTCGAGG-3′.

Thus, synthesized cDNAs were subcloned into an expression vector between *CMV* promoter in frame with an Aphthovirus 2A peptide-GFP fusion sequence for cotranslational cleavage [73]. This entire cassette was flanked by *Tol2* sites facilitating genomic integration upon cotransfection with Tol2 transposase as described [25]. ERR2VP16 was generated by fusing the open reading frame for the herpes simplex virus-1 (HSV-1) VP16 (amplified from *pActPL-VP16AD* plasmid, Addgene plasmid #15305, Watertown, USA) activation domain to ERR2. ERR2EnR was generated by fusing the open reading frame of the transcriptional repressor domain of *Drosophila* Engrailed (amplified from *CAG-EnR* plasmid, Addgene plasmid #19715, Addgene, Watertown, USA) to ERR2.

### Chick motor neuron electrophysiology

Chick embryos electroporated at E2.7 to E3.0 (HH stages 14 to 18) with appropriate DNA constructs were harvested at E12 to E15 (HH stages 38 to 41) and processed as described previously [25]. Briefly, chick embryos were placed on ice for 5 minutes, extracted from the egg, decapitated, and dissected in a petri dish containing cold chick aCSF (CaCSF) solution (mM): 139 NaCl, 3 KCl, 1 MgCl_2_, 17 NaHCO_3_, 12.2 dextrose, 3 CaCl_2._ The solution pH was adjusted to 7.25 with KOH and the osmolarity was adjusted to approximately 315 mOsm using sucrose. The thoracolumbar region of the spinal cord (with the vertebral column intact) was isolated and embedded in an agarose block (4% agarose in CaCSF) using 20% gelatin in CaCSF. Slices (370 μm) were obtained using a Leica VT1200 S vibrating blade microtome (Leica Biosystems GmbH, Nussloch, Germany) and incubated in CaCSF for 30 minutes at room temperature (22°C). Motor neurons were visualized by GFP expression using 4× air objective (Olympus UPlan FL N) and 40× water-immersion objective (Olympus UPlan FI N) equipped on an Olympus BX51W1 microscope. The patch pipette (resistances of 3 to 6 MΩ) was filled with intracellular solution (mM): 130 MeSO_3_H, 10 KCl, 2 MgCl_2_, 0.4 EGTA, 10 HEPES, 2 ATP-Mg^2+^ salt, 0.4 GTP-Na^+^ salt, 0.1 CaCl_2._ The solution pH was adjusted to 7.3 with KOH, and the osmolarity was adjusted to approximately 315 mOsm using sucrose. The intracellular solution contained 25 μM Alexa Fluor 568 dye (Thermo Fisher Scientific, Waltham, USA) to label recorded motor neurons. Current clamp recording signals were amplified and filtered using MultiClamp 700B patch-clamp amplifier (Molecular Devices, San Jose, USA). The signal acquisition was performed at 20 kHz using Digidata 1322A digitizer (Molecular Devices, San Jose, USA) and pCLAMP 10.4 software (Molecular Devices, San Jose, USA).

### RNA sequencing and transcriptome analysis

E12.5 (HH St. 38 to 39) chick lumbar spinal motor columns transfected with either *CMV*::*eGFP*, or *CMV*:: *ERR2VP16*.*2A*.*eGFP* or *Dlk1*.*IRES*.*eGFP* plasmids were identified by GFP fluorescence, dissected and collected. RNA isolation and RNA sequencing were carried out as described previously [73]. Briefly, RNA was isolated using Tri-Reagent (Sigma-Aldrich Chemie GmbH, Taufkirchen, Germany) and Phenol-Chloroform extraction according to the manufacturer’s protocol. RNA quality was assessed using Nanodrop 2000 (Thermo Fisher Scientific, GmbH) and RNA integrity number (RIN) was evaluated by using the Agilent 2100 Bioanalyzer (Agilent Technologies, USA). RNA was reverse transcribed to cDNA using Transcriptor High Fidelity cDNA synthesis kit (Roche Diagnostics Deutschland GmbH, Mannheim, Germany) and RNA-Seq libraries were obtained using TruSeq RNA Sample Preparation v2 kit (Illumina, San Diego, USA). To analyze the library quality, Agilent 2100 Bioanalyzer (Agilent Technologies, Santa Clara, USA) was used and the concentration was measured by a Qubit^R^ dsDNA HS Assay kit (Thermo Fisher Scientific, Waltham, USA). The concentration was adjusted to 2 nM prior to sequencing (50 bp) on a HiSeq 2000 sequencer (Illumina, San Diego, USA) using TruSeq SR Cluster kit v3-cBot-HS (Illumina, San Diego, USA) and TruSeq SBS kit v3-HS (Illumina, San Diego, USA) based on manufacturer’s instructions. RNA-sequencing quality was evaluated by utilizing raw reads using the FastQC quality control tool version 0.10.1 [74,75]. Bowtie2 v2.0.2 using RSEM version 1.2.29 with default parameters was utilized to align sequence reads (single-end 50 bp) to chicken reference genome (Galgal5) [76,77]. Prior to indexing, GFP, ERR2, Dlk-1, VP16, and IRES sequences and annotations were added to the reference genome (FASTA file) and annotations (GTF file). Ensembl annotations (version 86.5) with rsem-prepare-reference from RSEM software was used to index chicken reference genome [78]. Furthermore, sequence alignment of sequence reads and gene quantity was obtained through the use of rsem-calculate-expression. Rsem-calculate-expression resulted in sequence read count and TPM value (transcripts per million) for individual genes. DESeq2 package was used to carry out differential expression analysis [79]. Finally, genes with less than 5 reads (baseMean) were filtered, while genes with an adjusted *p*-value < 0.05 were classified as differentially expressed. Gene ontologies and categorization was performed using the DAVID Gene Functional Classification Tool [80].

### Enhancer identification and promoter assays

Evolutionary conserved noncoding genomic regions (ECRs) around the *Kcna10* genomic locus were identified using the ECR Browser [81] and screened for potential ERR2/ERR3 transcription factor binding sites using the JASPAR CORE database [82]. A 240-bp ECR 3.5 kb upstream of the *Kcna10* transcription start site with 3 putative ERR2/ERR3 binding sites was amplified from mouse genomic DNA using the following primers: Forward 5′-TCTCACAGCCCTGCTCATC-3′ and Reverse 5′-CTTGCCTGAGAACCTGATCTCC-3′ and subcloned into a reporter vector containing a minimal promoter followed by tdTomato coding sequence, which together were flanked by *Tol2* sites to facilitate stable genomic integration. To test promoter activity and potential regulation by ERR2/3, Lohmann LSL fertilized chick eggs were incubated until E2.7 to E3.0 (HH stages 14 to 18) and chick embryo neural tubes were electroporated in ovo using the ECM 830 electroporation system (BTX/Harvard Apparatus, Holliston, USA) as described [25]. *Kcna10*::*tdTomato* reporter plasmids together with either *CMV*::*2A*.*eGFP* or *CMV*::*VP16*:*ERR2*.*2A*.*eGFP* at a molar ratio of 1:1 were transfected.

## Supporting information

S1 Fig Gamma and alpha motor neurons distinguished by differential Fluro-Gold (FG) incorporation. **(A-D)** P14 spinal cord ventral horn (note: same specimen as in S3G–S3I Fig): gamma and alpha motor neurons can be distinguished by different levels of Fluoro-Gold (FG) incorporation and soma sizes (scale bar: 50 μm). **(A)** Arrowheads: examples of small soma-size motor neurons with high levels of FG incorporation expressing low or negligible levels of NeuN. **(B)** High levels of the gamma motor neuron marker ERR3 [27] in the FG^high^, NeuN^low^ motor neurons (arrowheads). **(C)** FG^high^ motor neurons are NeuN^low^ (arrowheads) (**D**) and ERR3^high^ (arrowheads). **(E, F)** Scatter plots of motor neuron fluorescence levels over soma sizes at P21 (*n =* 159) (note: data from same experiment depicted in S4Q and S4R Fig): identification of gamma motor neurons based on a combination of soma size and FG levels. **(E, F)** Gamma motor neurons with small somas and relatively high FG levels that express high ERR3 levels **(E)**, but low or negligible levels of NeuN **(F)**. Larger motor neurons with lower but substantial FG levels express low or negligible levels of ERR3 **(E)**, but mostly higher levels of NeuN **(F)**. **(G-I)** Control FG^high^ (gamma) motor neurons (black bars) exhibit significantly higher instantaneous firing frequency **(G)**, instantaneous gain **(H),** and lower AHP-half decay time **(I)** when compared to FG^low^ (alpha) motor neurons (gray bars). Statistically significant differences are indicated as: *p < 0.05, **p < 0.01, ***p < 0.001, n.s. = not significant, Student *t* test). Data for S1G-S1I Fig can be found in S1 Data and for S1E and S1F Fig in S2 Data.
(TIF)

S2 Fig Selective immunodetection of either ERR2 or ERR3. **(A-D)** Transversal sections of E6 chick spinal cords unilaterally transfected by murine ERR2 (mERR2) and eGFP. **(A, C)** Immunofluorescence with anti-ERR2 IgG2b detects transfected mERR2 but not endogenous chick ERR2 (cERR2) (scale bar: 100 μm). **(B, D)** Anti-ERR3 IgG2a detects at low levels of endogenous cERR3 (asterisks) but does not cross-react with transfected mERR2. **(E-H)** Transversal sections of E6 chick spinal cords unilaterally transfected by murine ERR3 (mERR3) and eGFP. **(E, G)** Anti-ERR2 IgG2b does not cross-react with mERR3. **(F, H)** Anti-ERR3 IgG2a detects transfected mERR3 and also detects at low levels of endogenous cERR3 (asterisk in contralateral spinal cord) but does not cross-react with transfected mERR2. **(I-N)** Transversal sections of P21 mouse spinal cord ventral horn. **(I)** No cross-reactivity of Alexa488 anti-IgG2a with anti-ERR2 IgG2b (scale bar: 50 μm). **(J)** Detection of anti-ERR2 IgG2b with Alex555 anti-IgG2b in a subset of motor neuron nuclei (arrowheads; note: lower levels detected in other motor neuron and interneuron nuclei). **(K)** Fluoro-Gold (FG)-traced motor neurons. Note: characteristic high levels of FG incorporation in small soma size motor neurons (arrowheads). **(L)** Detection of anti-ERR3 IgG2a with Alexa Fluor 488 anti-IgG2a in a subset of motor neuron nuclei (arrowheads; note: lower levels detected in other motor neuron and moderate-to-high levels in interneuron nuclei) (scale bar: 50 μm). **(M)** No cross-reactivity of Alexa Fluor 555 anti-IgG2b with anti-ERR3 IgG2a. **(N)** FG-traced motor neurons. **(O-Q)** Transversal sections of P21 ERR2/3^cko^ (*Esrrb*^*flox/flox*^; *Esrrg*^*flox/flox*^; *Chat*^*Cre*^) mouse spinal cord ventral horn. (**O**) FG-traced motor neurons overlaid with ERR2 and ERR3 immunoreactivity. Closed arrowheads: small soma size FG^high^ motor neurons (scale bar: 50 μm). **(P, Q)** Absence of ERR2 **(P)** or ERR3 **(Q)** immunoreactivity in ERR2/3^cko^ motor neurons (closed arrowheads). Open arrowheads: ERR2 and ERR3 expression is retained in interneurons in ERR2/3^cko^.
(TIF)

S3 Fig High levels of ERR2 and ERR3 are coexpressed by gamma motor neurons. **(A-F)** Overview of transversal section of P21 *Chat*::tdTomato (*Chat*^*Cre*^; *Rosa26*^*fxtdTomato*^) lumbar mouse spinal cord. **(A)** Expression of tdTomato, NeuN, ERR2, and ERR3. tdTomato (magenta) mostly labels motor neurons in lamina IX but also subsets of cholinergic interneurons in laminae III and X (see S4A Fig) (scale bar: 200 μm). High levels of ERR2 and ERR3 coexpression in tdTomato^+^ NeuN^low^ motor neurons (yellow-orange nuclei in boxed areas). Boxed areas around left and right lateral motor columns correspond to the higher magnification given in G-K and L-O, respectively. **(B)** Highest levels of ERR2 in motor neurons (boxed area), relatively lower but detectable levels in other motor neuron subtypes and subsets of interneurons throughout the spinal cord. **(C)** High levels of ERR3 in motor neurons (boxed area), lower but significant levels in other motor neuron subtypes, and occasional high levels in interneurons in the intermediate spinal laminae. **(D)** High levels of ERR2 and ERR3 coexpression in NeuN^low^ motor neurons (yellow-orange nuclei in boxed areas) (scale bar: 200 μm). **(E, F)** Separate channels depicting expression of NeuN only **(E)** or tdTomato only **(F)**. **(G-K)** Higher magnification of left ventral horn (boxed area) in **(A-F)**. Closed arrowheads: Highest levels of ERR2 **(G)** and ERR3 **(H)** in consistently NeuN^low^ **(J)** and tdTomato^+^ **(K)** small motor neurons (scale bar: 50 μm). Note that small NeuN^low^ motor neurons consistently exhibit lower tdTomato levels compared to large motor neurons **(K, O)**. Open arrowheads: low but detectable ERR2 **(G)** and ERR3 **(H)** levels in tdTomato^−^ interneurons **(K)**. Triangles: low but detectable ERR2 **(G)** and ERR3 **(H)** levels in large tdTomato^+^ motor neurons **(K)** with intermediate to high NeuN levels **(J)**. Note that NeuN levels vary considerably in large motor neuron subtypes (see Fig 2B and 2F). **(L-O)** Higher magnification of right ventral horn (boxed area) in **(A-F)**. Closed arrowheads: Highest levels of ERR2 **(L)** and ERR3 **(M)** in consistently NeuN^low^ **(N)** and tdTomato^+^ **(O)** small motor neurons (scale bar: 50 μm). Open arrowheads: low but detectable ERR2 **(L)** and ERR3 **(M)** levels in tdTomato^−^ interneurons **(O)**. Triangles: low but detectable ERR2 **(L)** and ERR3 **(M)** levels in large tdTomato^+^ motor neurons **(O)** with high NeuN levels **(N)**. (**P–T**) Consistent patterns of high ERR2 and ERR3 levels in small soma-size NeuN^low^ motor neurons at cervical-brachial levels (scale bar: 200 μm).
(TIF)

S4 Fig Progressive confinement of high ERR2 levels to gamma motor neurons during postnatal development depends on muscle spindle-derived signals. **(A-C)** Expression of ERR2 in most Isl1^+^ motor neurons in E10.5 mouse embryo thoracic neural tube (scale bar: 50 μm). **(D-F)** At P4 higher levels of ERR2 **(D)** have begun to be mostly confined to small NeuN^low or negligible^ **(E)** motor neurons, while many NeuN^high^ motor neurons still retain relatively lower yet substantial ERR2 levels (compare **D** and **E)**. **(F)** Fluoro-Gold (FG) tracing to visualize motor neurons. **(G-H)** At P14, high ERR2 levels **(G)** are further confined to small NeuN^low or negligible^ motor neurons **(H)** with high levels of FG incorporation **(I)**. **(J-K)** At P21, high ERR2 levels **(J)** are further confined to small NeuN^low or negligible^ motor neurons **(K)** with high levels of FG retention **(L)**, while ERR2 levels in NeuN^high^ motor neurons have dropped further compared to earlier postnatal stages (compare **D**, **G,** and **J**). **(M-Q)** Scatter plots of ERR2 (red data points) and ERR3 (green) levels over soma sizes show increasing confinement of high-levels of ERR2 and ERR3 to a population of small motor neurons during postnatal development ages of P4 (*n =* 100), P14 (*n* = 189), and P21 (*n* = 159). **(M-R)** Scatter plots of ERR2 (red data points) and NeuN (blue) levels over soma sizes show increasing segregation of motor neurons into ERR2^high^, NeuN^low^ and ERR2^low^, NeuN^high^ populations during postnatal development, while also revealing considerably cell-to-cell variability in relative expression levels of ERR2, ERR3, and NeuN. **(SS’-XX’)** Motor neurons fail to retain high ERR2 and ERR3 levels in mice with defective spindle development. **(S-X)** Control (transversal section of lumbar spinal cord of adult mouse): expression of high ERR2 **(V)** and ERR3 **(W)** levels by VACHT^+^ **(X)** NeuN^low or negligible^ **(T)** motor neurons (closed arrowheads). Open arrowheads: expression of ERR2 and ERR3 by subsets of VACHT^−^ interneurons. Triangle: low ERR2 and ERR3 levels in some large motor neurons. Note: VACHT^+^ cytosol identifies motor neurons in lamina IX, while VACHT^+^ varicosities indicate cholinergic synapses or axons stemming from nonmotor neurons. **(S’-X’)** Egr3^ko^ (Egr3^*−/−*^) transversal section of lumbar spinal cord of adult mouse: absence of high ERR2 **(V’)** and ERR3 **(W’)** levels in VACHT^+^ **(X’)** NeuN^low or negligible^ **(T’)** motor neurons. Open arrowheads: retention of ERR2 and ERR3 expression by VACHT^−^ interneurons (compare **V**, **W,** and **X**). Triangles: retention of low ERR2 and ERR3 levels by large motor neurons (compare **V**, **W,** and **X**). *n* = # of neurons. Data for S4M-S4R Fig can be found under S2 Data.
(TIF)

S5 Fig Expression of ERR2 and ERR3 in motor neurons and noncholinergic interneurons in spinal cord and brain. **(A, B)** Overview of transversal section of P21 *Chat*::tdTomato (*Chat*^*Cre*^; *Rosa26*^*fxtdTomato*^) thoracic mouse spinal cord. **(A)** Expression of tdTomato indicating *Chat*^*Cre*^ activity in motor neurons (lamina IX), interneurons (presumably V0c) in lamina X and lamina III interneurons, as well as few scattered neurons in the intermediate laminae (scale bar: 200 μm). **(B)** ERR2 and ERR3 expression in tdTomato^+^ motor neurons but not in other tdTomato^+^ neurons outside lamina IX. **(C, D)** No detectable ERR2 and ERR3 expression in tdTomato^+^ lamina X interneurons (arrowheads) (scale bar: 50 μm). **(E, F)** No detectable ERR2 and ERR3 expression in tdTomato^+^ lamina III interneurons (arrowheads) (scale bar: 200 μm). **(G, H)** Expression of ERR2 and ERR3 by oculomotor neurons (III) (arrowheads) and largely nonoverlapping expression of either ERR2 or ERR3 by subsets of noncholinergic neurons throughout the midbrain (scale bar: 500 μm). **(I, J)** Expression of ERR2 and ERR3 by most but not all cholinergic neurons of the oculomotor nucleus (scale bar: 50 μm). **(K, L)** Expression of ERR2 and ERR3 in a subset of noncholinergic (tdTomato^−^) cortical interneurons (scale bar: 100 μm). (**M-O**) By E12.5, most if not all motor neurons in the ventral horn are tdTomato^+^ (scale bar: 250 μm). Brackets: enlarged areas shown in (**P–T**) (scale bar: 50 μm). Green signal: VACHT.
(TIF)

S6 Fig Expression of ERR2 and ERR3 in noncholinergic interneurons in the brain, including areas related to motor or vestibular function. **(A-E)** Overview of sagittal section of a *Chat*::tdTomato (*Chat*^*Cre*^; *Rosa26*^*fxtdTomato*^) mouse brain assembled from Z-stacked laser scanning microscopy images. ERR2 and ERR3 were co-immunodetected. Details of brain structures indicated in (**A-E**) are shown next to the overview sections in higher magnification, labelled by the abbreviations for the brain structures pointed out in (**A-E**) (scale bar: 1,000 μm). Higher-magnification images show consistent nonoverlapping expression of tdTomato and ERR2 and ERR3 (scale bar: 50 μm). (**ml, medial lemniscus**) tdTomato^+^ axons passing through area with scattered ERR2/3^+^cells. (**RT, reticular nucleus of the thalamus**) Absence of overlap between tdTomato^+^ and ERR2/3^+^ cells. (**MB, midbrain**) Absence of overlap between tdTomato^+^ and ERR2/3^+^ cells. (**CP, caudoputamen**) Absence of overlap between tdTomato^+^ and ERR2/3^+^ cells. (**CB, cerebellum**) Absence of overlap between tdTomato^+^ (granular cell layer) and ERR2/3^+^ (Purkinje cell layer) cells. (**CB, cerebellum**) Absence of overlap between tdTomato^+^ (granular cell layer) and ERR2/3^+^ (Purkinje cell layer) cells. (**CP, caudoputamen**, posterior aspect) Absence of overlap between tdTomato^+^ and ERR2/3^+^ cells. (**HY, hypothalamus**) Absence of overlap between tdTomato^+^ (fiber tracts) and very few ERR2/3^+^ cells. (**IP, interposed nucleus**) Absence of overlap between very few tdTomato^+^ and ERR2/3^+^ cells. (**SUV, superior vestibular nucleus**) Absence of overlap between very few tdTomato^+^ cells and axons and ERR2/3^+^ cells. (**TH, thalamus**) Absence of overlap between very few tdTomato^+^ cells and axons and scattered ERR2/3^+^ cells.
(TIF)

S7 Fig Electrophysiological properties of gamma and alpha motor neurons in control and ERR2/3^cko^ mice. **(A-E)** ERR2/3^cko^ FG^high^ (gamma) motor neurons (red bars) exhibit significantly lower instantaneous firing frequency **(A)**, lower instantaneous gain **(B)**, higher AHP-half decay time **(C)**, higher capacitance **(D)**, while no significant difference input resistance **(E),** when compared to control FG^low^ motor neurons (blue bars), respectively (see S1 Table for details). **(F-J)** No significant differences between ERR2/3^cko^ FG^low^ (alpha) motor neurons (red bars) and control FG^low^ (alpha) motor neurons (red bars) when comparing instantaneous firing frequency **(F)**, instantaneous gain **(G)**, AHP-half decay time **(H)**, capacitance **(I),** and input resistance **(J),** respectively (see S1 Table for details). Data for S7A-S7J Fig can be found in S1 Data.
(TIF)

S8 Fig Establishment of intrafusal and extrafusal motor innervation and expression of alpha motor neuron markers in the absence of ERR2/3. (**A-F**) P100 mouse soleus muscle spindles of control (heterozygous: *Esrrb*^*flox/+*^*;Esrrg*^*flox/+*^*;Chat*^*Cre*^*)*. (**A-C**) and ERR2/3^cko^ (**D-F**) mice. (**A-F**) Comparable distribution of Ia sensory annulospiral endings in the central spindle segment (visualized by VGLUT1, green), motor innervation (CHAT, magenta) and their postsynaptic sites (BTX, alpha bungarotoxin, grey) between control (**A-C**) and ERR2/3^cko^ (**D-F**) mice (scale bar: 100 μm). Arrowheads: Comparable motor innervation (CHAT, magenta) of postsynaptic sites on the peripheral segments of intrafusal fibers (BTX, alpha bungarotoxin, grey) in control **(A-C**) and ERR2/3^cko^ (**D-F)** mice. Scale bars: 100 μm. **(G-N**) Sections through EDL (**G,H, K, L**) and soleus (I, J, **M, N**) muscle of P100 mice: comparable characteristic pretzel-like morphologies of neuromuscular junctions (NMJs) with extrafusal muscle fibers and consistent motor innervation (VACHT^+^) of all postsynaptic NMJs (BTX^+^) and innervation in control **(G, H, I, J**) and ERR2/3^cko^ (**K, L, M, N)** mice. Scale bars: 100 μm.
(TIF)

S9 Fig Expression of gamma and alpha motor neuron markers in the absence of ERR2/3. (**A-H**) Transversal sections of adult *Chat*::*tdTomato* lumbar mouse spinal cords of control **(A-D**) and ERR2/3^cko^ (**E-H**) mice. (**A-D**) Expression of high levels of GFRA1 by ERR3^high^, NeuN^low^, and tdTomato^+^ small soma-size motor neurons (arrowheads), consistently lower levels in larger some-size ERR3^high^, NeuN^low^, and tdTomato^+^ motor neurons in control mice. (**E-F**) Persistent expression of high GFRA1 levels in NeuN^low^ small soma-size motor neurons (arrowheads) in ERR2/3^cko^ mice. (**I-T**) Transversal sections of adult mouse spinal cords of control **(I-K**) and ERR2/3^cko^ (**L-N**) mice. **(I-K**) High levels of expression of the fast-alpha motor neuron marker MMP9 by large some-size VACHT^+^ motor neurons in control mice. LMC, lateral motor column; MMC, medial motor column. (**L-N**) Persistent high MMP9 levels in large some-size VACHT^+^ motor neurons in ERR2/3^cko^ mice. (**O-T**) Transversal sections of P400 *mice* spinal cords of control **(O-Q**) and ERR2/3^cko^ (**R-T**) mice. **(O-Q**) High levels of expression of the slow/intermediate alpha motor neuron marker OPN by subsets of intermediate some-size VACHT^+^ motor neurons in control mice. (**R-T**) Persistent high OPN levels in subsets of large some-size VACHT^+^ motor neurons in ERR2/3^cko^ mice. (Scale bars: 100 μm).
(TIF)

S10 Fig Gait alterations in ERR2/3^cko^ mice. **(A-C)** Polygon graphs based on partial least squares (PLS) analysis of 58 gait variables measured during treadmill locomotion at 10 m•s^−1^ **(A)**, 20 m•s^−1^ **(B),** and 35 m•s^−1^ **(C)**. Optimized model prediction was used to assign data sets for fore and hind limbs to either genotype (control versus ERR2/3^cko^) and the two components of the models were plotted against each other. Each one of the tested animals is represented by a single dot, while polygons group the animals of the same genotype. The amount of between-groups variance explained by each component in the model is expressed in percent of the total between-groups variance. The two components of our optimized models captured more than 25% of the variance in the predictors in both fore and hind limb at all treadmill speeds. These scores indicate that the method was able to capture the maximum variance between genotypes in the first dimension, which is also shown by the absence of overlap between the two groups on the x-axis, together indicating that ERR2/3^cko^ exhibit significant gait alterations compared to control mice. Yet, all ERR2/3^cko^ mice analyzed were able to successfully complete the treadmill locomotion tasks at all speeds tested (provided as the number of animals “n” running until “completion” for each speed). **(D, E)** Ranking of the variables’ predictive capacities in the forelimb **(D)** and hind limb **(E)** models. The most predictive parameters display the highest loadings (arbitrary units) independent of their sign. The mean value of all mice from the same group was calculated for each parameter and depicted in the bar charts. The sign of the loadings only indicates the direction in which gait parameters are affected (increased, red, or reduced, blue, in ERR2/3^cko^ compared to control mice). **(F-I)** Examples of Ia afferent recordings from extensor digitorum longus (EDL) nerve-muscle preparations. Red traces: Ia afferent firing, blue traces: relative muscle length, green traces: relative muscle tension upon application of muscle stretch with a force transducer. **(F, G)** In the control preparation, Ia afferents exhibit relatively low tonic firing rates at resting length, which rapidly increases upon application of muscle stretch with two characteristic bursts during stretch onset and offset. **(H, I)** In Err2/3^cko^ mice, Ia afferents frequently fall silent during resting length, while exhibiting a responsiveness towards stretch similar to control Ia afferents. Data for S10A-S10E Fig can be found in S3 Data.
(TIF)

S11 Fig Gene expression signature mediated by ERR2 in chick motor neurons and direct activation of *Kcna10* promoter by ERR2 in chick. **(A)** Numbers of RNA reads for endogenous chick *Esrrb* (*cEsrrb*) and mouse *Esrrb* (*mEsrrb*) in control (eGFP only) expressing chick motor neurons or chick motor neurons expressing mouse *Esrrb* (*mEsrrb*): RNA sequencing reveals unaltered *cEsrrb* (24.60 ± 3.15 versus 24.79 ± 2.45) expression by forced *mEsrrb* expression, no reads of *mEsrrb* in the control sample (as expected) and approximately 6-fold overexpression of *mEsrrb* (148.89 ± 27.03) relative to endogenous *cEsrrb* levels (24.60 ± 3.15). **(B)** Examples of genes regulated by ERR2 in chick motor neurons (given in fold change over control motor neurons), including genes encoding the voltage-gated potassium and chloride channels *Kcna10* (13.65 ± 3.28) and *cClcnkb* (11.85 ± 2.51), respectively, as well as the not yet annotated gene *Ensgal00000031003* (19.20 ± 5.25). Expression of “structural” gene *Actb* (0.94 ± 0.05), as well as of *Hoxa11* (0.93 ± 0.00) and *Hoxc11* (1.03 ± 0.01) related to motor pool identities did not significantly change. (**C**) Clustered David functional annotations of genes differentially regulated by forced ERR2 in chick motor neurons (“clustered” refers to genes associated with more than one annotated GO term), significance of classification is given as logarithmic *p*-value. Gene ontology terms include “LTP” (“long-term potentiation”: glutamate receptor *Grin2a*, neurotrophin receptor *Ntrk2*, sodium/potassium/calcium exchanger *Slc24a2* and tenascin R-encoding *Tnr*), “ion transport” and “ion channel” (*cClcnkb*, *Kcna10*, *Grin2a*, *Slc24a2*, potassium channel tetramerization domain containing *Kctd4*, gamma-aminobutyric acid receptor subunit *Gabrd*, voltage-gated sodium channel subunit *Scn5a*, voltage-gated potassium channel *Kcnh1*, transferrin, *TF* and Tweety-homolog 3-encoding *Ttyh3*), “transport” (adding translocase of inner mitochondrial membrane 10 homolog *TIMM10*, mitochondrial adenine nucleotide translocator *SLC25A4* to the “ion transport” list) and extracellular matrix (*Tnr*, Netrin1 *Ntn1* and aggrecan-encoding *Acan*). **(D)** GO terms for the genes differentially regulated by ERR2 after subtracting the genes differentially regulated by Dlk1, leaving “ion transport” and “transport” as the only significantly clustered GO terms (including *Kcna10*, *cClcnkb*, *TF*, *Kctd4*). **(E)** Alignment of evolutionary conserved on-coding genomic region (ECR) upstream of the *Kcna10* transcription start site between human and mouse. Predicted ERR2/3 binding sites are highlighted in red (see Fig 7B). **(F-K)** Examples of tdTomato expression driven from a minimal promoter-tdTomato expression construct by the *Kcna10* ECR upon cotransfecting eGFP only (“mock”) (control) **(F, G)**, ERR2 and eGFP **(H, I)**, as well as the *Kcna10* ECR with scrambled ERR2/3 binding sites plus ERR2 and eGFP **(J, K)** (scale bar: 50 μm). (**L-O**) Expression of *Esrrb* and *Esrrg* mRNAs detected by in situ hybridization in transversal sections of E18 chick spinal cords. (**L**) Largely uniform expression of *Chat* in motor neurons in ventral horn. (**M**) High levels of *Esrrb* expression by subsets of neurons in ventral horn, but lower levels in other neurons. (**N**) High levels of *Esrrg* expression by subsets of neurons in ventral horn, but lower levels in other neurons. (**O**) Higher magnification of (**M**): *Esrrb*^*high*^ and *Esrrb*^*low*^ neurons can be distinguished in ventral horn. Data for S11A-S11D Fig can be found in S5 Data. The complete set of values for the RNAseq experiments can be found in S6 Data.
(TIF)

S1 Table Summary of parameters recorded via whole-cell patch clamping of mouse motor neurons in acute spinal cord slices. Genotypes of the recorded animals are given in the table. Values show mean ± standard error of the mean (SEM). ^**a**^ indicates significant difference between control FG^high^ (*n =* 24) and control FG^low^ (*n* = 22) (Student *t* test); ^**b**^ indicates significant difference between control FG^high^ (*n* = 24) and ERR2/3^cko^ FG^high^ (*n* = 18) (Student *t* test); ^**c**^ indicates significant difference between control FG^low^ (*n* = 22) and ERR2/3^cko^ FG^low^ (*n* = 9) (Student *t* test); ***p-value < 0.001; **p-value < 0.01; *p-value < 0.05; n.s., not significant. *n* = # of neurons.
(TIF)

S2 Table Summary of parameters recorded via whole-cell patch clamping of chick motor neurons in acute spinal cord slices. Constructs used to stably transfect the motor neurons prior to the recordings are in the table. CMV-eGFP-control 1 versus CMV-VP16-ERR2-eGFP, CMV-ERR2-eGFP, CMV-ERR3-eGFP, CMV-EnR-ERR2-eGFP, and CMV-eGFP-control 2 versus CMV-KCNA10-eGFP. Values show mean ± standard error of the mean (SEM). ^**a**^ indicates significant difference compared to CMV-eGFP-control 1 (Student *t* test); ^**b**^ indicates significant difference between CMV-eGFP-control 1 and CMV-eGFP-control 2 (Student *t* test); ^**c**^ indicates significant difference between CMV-eGFP-control 2 and CMV-KCNA10-eGFP; ^**d**^ indicates significant difference compared to CMV-eGFP-control 2 (Student *t* test); (Student *t* test); ***p-value < 0.001; **p-value < 0.01; *p-value < 0.05; n.s., not significant.
(TIF)

S3 Table Gene expression signatures promoted by forced ERR2:Vp16 expression. (TIF)

S1 Movie Example of an adult *Esrrb*^*+/+*^*;Esrrg*^*flox/+*^ (without *Chat*^*Cre*^) mouse freely moving in an empty cage, showing normal movements. (MP4)

S2 Movie Example of an adult *Esrrb*^*+/+*^*;Esrrg*^*flox/+*^*;Chat*^*Cre*^ mouse freely moving in an empty cage, showing movements indistinguishable from control (see S1 Movie). (MP4)

S3 Movie Example of an adult *Esrrb*^*flox/+*^*;Esrrg*^*flox/+*^*;Chat*^*Cre*^ mouse freely moving in an empty cage, showing movements almost indistinguishable from control (see S1 Movie). (MP4)

S4 Movie Example of an adult *Esrrb*^*flox/flox*^*;Esrrg*^*+/+*^*;Chat*^*Cre*^ mouse freely moving in an empty cage, showing very mild movement aberrations (slightly unsteady, quaking gait and posture, slightly abnormal tail positioning), compared to control (see S1 Movie). (MP4)

S5 Movie Example of an adult *Esrrb*^*+/+*^*;Esrrg*^*floxflox*^*;Chat*^*Cre*^ mouse freely moving in an empty cage, showing apparent movement aberrations (unsteady, quaking gait and posture, abnormal tail positioning), compared to control (see S1 Movie), yet relatively milder compared to *Esrrb*^*flox/+*^*;Esrrg*^*floxflox*^*;Chat*^*Cre*^ (see S1 Movie). (MP4)

S6 Movie Example of an adult *Esrrb*^*flox/+*^*;Esrrg*^*floxflox*^*;Chat*^*Cre*^ mouse freely moving in an empty cage, showing pronounced movement aberrations (unsteady, quaking gait and posture, abnormal tail positioning), compared to control (see S1 Movie). (MP4)

S7 Movie Example of a control mouse navigating a horizontal beam. The animal movement was tracked using GoPro HD Hero2 (GoPro, San Mateo, USA) fitted to a custom-built slider track. The video’s frame rate (acquired at 120 fps) was reduced to 60 fps and speed was slowed to 25%–40% of the original speed.
(MP4)

S8 Movie Example of an *ERR2/3*^*cko*^ mouse navigating a horizontal beam. The video was acquired and modified as above (see S7 Movie).
(MP4)

S9 Movie Example of a control mouse navigating a horizontal ladder. The video was acquired and modified as above.
(MP4)

S10 Movie Example of an *ERR2/3*^*cko*^ mouse navigating a horizontal ladder. The video was acquired and modified as above (see S9 Movie).
(MP4)

S1 Data Data that underlie Figs 1D, 1E, 3B, 3C, 3E, 3F, S1G–S1I, and S7A–S7J. (XLSX)

S2 Data Data that underlie Figs 2I, 2J, 2K, 2N, 4I, 7D–7G, 8I, 8J, S1E, S1F, and S4M–S4R. (XLSX)

S3 Data Data that underlie Figs 5A, 5B, 5D, 5F and S10A–S10E. (XLSX)

S4 Data Data that underlie Fig 6G–6J. (XLSX)

S5 Data Data that underlie Figs 7A, 7C and S11A–S11D. (XLSX)

S6 Data Data that underlie Fig 7A. (XLSX)

## Abbreviations

DI: dynamic index
DP: dynamic peak discharge
ECR: evolutionary conserved region
EDL: extensor digitorum longus
ERR: estrogen-related receptor
FG: Fluoro-Gold
HSV-1: herpes simplex virus-1
MN: motor neuron
OSC-PLS: Orthogonal Signal Correction PLS
PLS: Partial Least Squares
RD: resting discharge
RIN: RNA integrity number
RMSEP: Root Mean Square Error of Prediction
ROI: region of interest
SR: static response

## References

[1] Granit R. The functional role of the muscle spindles—facts and hypotheses. Brain. 1975 Dec;98(4):531–56. doi: 10.1093/brain/98.4.531 . 130185

[2] Proske U, Gandevia SC. The proprioceptive senses: their roles in signaling body shape, body position and movement, and muscle force. Physiol Rev. 2012 Oct;92(4):1651–97. doi: 10.1152/physrev.00048.2011 . 23073629

[3] Barker D. The morphology of muscle receptors (In: Handbook of Sensory Physiology Vol. III/2, Muscle Receptors. In: Hunt CC, editor. Berlin-Heidelberg-New York: Springer-Verlag; 1974.

[4] Bessou P, Emonet-Denand F, Laporte Y. Occurrence of Intrafusal Muscle Fibres Innervation by Branches of Slow α Motor Fibres in the Cat. Nature. 1963;198(4880):594–95.

[5] Katz B. The efferent regulation of the muscle spindle in the frog. J Exp Biol.1949 Aug;26(2): 201–17. doi: 10.1242/jeb.26.2.201 . 15395191

[6] Proske U. Responses of Muscle Spindles in the Lizard. Nature. 1967 Mar 18;213(5081):1144–46. doi: 10.1038/2131144a0 . 6029803

[7] Kernell D. The Motoneuron and Its Muscle Fibers. 1st ed. New York: Oxford Univ. Press; 2006.

[8] Leksell L. The action potentials and excitatory effects of the small ventral root fibres to skeletal muscle. Acta Physiol Scand. 1945;10:1(Suppl. 31):1–84.

[9] Kuffler SW, Hunt CC, Quilliam JP. Function of medullated small-nerve fibers in mammalian ventral roots; efferent muscle spindle innervation. J Neurophysiol. 1951 Jan;14(1):29–54. doi: 10.1152/jn.1951.14.1.29 . 14784872

[10] Murthy KS. Vertebrate fusimotor neurones and their influence on motor behavior. Prog Neurobiol. 1978;11(3–4):249–307. doi: 10.1016/0301-0082(78)90015-1 . 154690

[11] Maier A. The avian muscle spindle. Anat Embryol. 1992;186(1):1–25. doi: 10.1007/BF00710398 . 1387513

[12] Dorward PK. Response characteristics of muscle afferents in the domestic duck. J Physiol. 1970 Nov;211(1):1–17. doi: 10.1113/jphysiol.1970.sp009262 . 5500993 PMC1395594

[13] Eccles JC, Eccles RM, Lundberg A. The convergence of monosynaptic excitatory afferents onto many different species of alpha motoneurones. J Physiol. 1957 Jun 18;137(1):22–50. doi: 10.1113/jphysiol.1957.sp005794 . 13439582 PMC1362996

[14] Eccles JC, Eccles RM, lggo A, Lundberg A. Electrophysiological studies on gamma motoneurones. Acta Physiol Scand. 1960 Sept 30;50:32–40. doi: 10.1111/j.1748-1716.1960.tb02070.x . 13725574

[15] Westbury DR. A comparison of the structures of alpha- and gamma-spinal motoneurones of the cat. J Physiol. 1982 Apr;325:79–91. doi: 10.1113/jphysiol.1982.sp014137 . 7108785 PMC1251381

[16] Ellaway PH, Taylor A, Durbaba R. Muscle spindle and fusimotor activity in locomotion. J Anat. 2015 Aug;227(2):157–66. doi: 10.1111/joa.12299 . 26047022 PMC4523318

[17] Dimitriou M. Human muscle spindles are wired to function as controllable signal-processing devices. Elife. 2022 Jul 13;11: e78091. doi: 10.7554/eLife.78091 . 35829705 PMC9278952

[18] Brownstone RM, Bui TV, Stifani, N. Spinal circuits for motor learning. Curr Opin Neurobiol. 2015 Aug;33:166–73. doi: 10.1016/j.conb.2015.04.007 . 25978563

[19] Kemm RE, Westbury DR. Some properties of spinal gamma-motoneurones in the cat, determined by micro-electrode recording. J Physiol. 1978 Sept;282:59–71. doi: 10.1113/jphysiol.1978.sp012448 . 722563 PMC1282724

[20] Stifani N. Motor neurons and the generation of spinal motor neuron diversity. Front Cell Neurosci. 2014 Oct 9;8:293. doi: 10.3389/fncel.2014.00293 . 25346659 PMC4191298

[21] Hobert O, Kratsios P. Neuronal identity control by terminal selectors in worms, flies, and chordates. Curr Opin Neurobiol. 2019 Jun;56:97–105. doi: 10.1016/j.conb.2018.12.006 . 30665084

[22] Zeng H, Sanes JR. Neuronal cell-type classification: challenges, opportunities and the path forward. Nat Rev Neurosci 2017 Sept;18(9):530–46. doi: 10.1038/nrn.2017.85 . 28775344

[23] Kurmangaliyev YZ, Yoo J, LoCascio SA, Zipursky SL. Modular transcriptional programs separately define axon and dendrite connectivity. Elife. 2019;5(8):e50822. doi: 10.7554/eLife.50822 . 31687928 PMC6855804

[24] Zengel JE, Reid SA, Sypert GW, Munson JB. Membrane electrical properties and prediction of motor-unit type of medial gastrocnemius motoneurons in the cat. J Neurophysiol. 1985 May;53(5):1323–44. doi: 10.1152/jn.1985.53.5.1323 . 3839011

[25] Müller D, Cherukuri P, Henningfeld K, Poh CH, Wittler L, Grote P, et al. Dlk1 promotes a fast motor neuron biophysical signature required for peak force execution. Science. 2014 Mar 14;343(6176):1264–66. doi: 10.1126/science.1246448 . 24626931

[26] Kelley KW, Ben Haim L, Schirmer L, Tyzack GE, Tolman M, Miller JG, et al. Kir4.1-Dependent Astrocyte-Fast Motor Neuron Interactions Are Required for Peak Strength. Neuron. 2018 Apr 18;98:306–19. doi: 10.1016/j.neuron.2018.03.010 . 29606582 PMC5919779

[27] Friese A, Kaltschmidt JA, Ladle DR, Sigrist M, Jessell TM, Arber S. Gamma and alpha motor neurons distinguished by expression of transcription factor Err3. Proc Natl Acad Sci U S A. 2009 Aug 11;106(32):13588–93. doi: 10.1073/pnas.0906809106 . 19651609 PMC2716387

[28] Shneider NA, Brown MN, Smith CA, Pickel J, Alvarez FJ. Gamma motor neurons express distinct genetic markers at birth and require muscle spindle-derived GDNF for postnatal survival. Neural Dev. 2009 Dec 2;4:42. doi: 10.1186/1749-8104-4-42 . 19954518 PMC2800842

[29] Ashrafi S, Lalancette-Hébert M, Friese A, Sigrist M, Arber S, Shneider NA, et al. Wnt7A identifies embryonic γ-motor neurons and reveals early postnatal dependence of γ-motor neurons on a muscle spindle-derived signal. J Neurosci. 2012 Jun 20;32(25):8725–31. doi: 10.1523/JNEUROSCI.1160-12.2012 . 22723712 PMC3496251

[30] Enjin A, Leão KE, Mikulovic S, Le Merre P, Tourtellotte WG, Kullander K. Sensorimotor function is modulated by the serotonin receptor 1d, a novel marker for gamma motor neurons. Mol Cell Neurosci. 2012 Mar;49(3):322–32. doi: 10.1016/j.mcn.2012.01.003 . 22273508 PMC3306528

[31] Edwards IJ, Bruce G, Lawrenson C, Howe L, Clapcote SJ, Deuchars SA, et al. Na+/K+ ATPase α1 and α3 isoforms are differentially expressed in α- and γ-motoneurons. J Neurosci. 2013 Jun 12;33(24):9913–9. doi: 10.1523/JNEUROSCI.5584-12.2013 . 23761886 PMC3722489

[32] Rosenberg AB, Roco CM, Muscat RA, Kuchina A, Sample P, Yao Z, et al. Single-cell profiling of the developing mouse brain and spinal cord with split-pool barcoding. Science. 2018 Apr 13;360 (6385):176–82. doi: 10.1126/science.aam8999 . 29545511 PMC7643870

[33] Alkaslasi MR, Piccus ZE, Hareendran S, Silberberg H, Chen L, Zhang Y, et al. Single nucleus RNA-sequencing defines unexpected diversity of cholinergic neuron types in the adult mouse spinal cord. Nat Commun. 2021 Apr 30;12(1):2471. doi: 10.1038/s41467-021-22691-2 . 33931636 PMC8087807

[34] Blum JA, Klemm S, Shadrach JL, Guttenplan KA, Nakayama L, Kathiria A, et al. Single-cell transcriptomic analysis of the adult mouse spinal cord reveals molecular diversity of autonomic and skeletal motor neurons. Nat Neurosci. 2021 Apr;24(4):572–83. doi: 10.1038/s41593-020-00795-0 . 33589834 PMC8016743

[35] Akhavan M, Hoang TX, Havton LA. Improved detection of fluorogold-labeled neurons in long-term studies. J Neurosci Methods. 2006 Apr 15;152(1–2):156–62. doi: 10.1016/j.jneumeth.2005.09.010 . 16246425

[36] Hong H, Yang L, Stallcup MR. Hormone-independent transcriptional activation and coactivator binding by novel orphan nuclear receptor ERR3. J Biol Chem. 1999 Aug 6;274(32):22618–26. doi: 10.1074/jbc.274.32.22618 . 10428842

[37] Huss JM, Garbacz WG, Xie W. Constitutive activities of estrogen-related receptors: Transcriptional regulation of metabolism by the ERR pathways in health and disease. Biochim Biophys Acta. 2015 Sep;1852(9):1912–27. doi: 10.1016/j.bbadis.2015.06.016 . 26115970

[38] Dufour CR, Wilson BJ, Huss JM, Kelly DP, Alaynick WA, Downes M, et al. Genome-wide orchestration of cardiac functions by the orphan nuclear receptors ERRalpha and gamma. Cell Metab. 2007 May;5 (5):345–56. doi: 10.1016/j.cmet.2007.03.007 . 17488637

[39] Enjin A, Rabe N, Nakanishi ST, Vallstedt A, Gezelius H, Memic F, et al. Identification of novel spinal cholinergic genetic subtypes disclose Chodl and Pitx2 as markers for fast motor neurons and partition cells. J Comp Neurol. 2010 Jun 15;518(12):2284–304. doi: 10.1002/cne.22332 . 20437528

[40] Akay T, Tourtellotte WG, Arber S, Jessell TM. Degradation of mouse locomotor pattern in the absence of proprioceptive sensory feedback. Proc Natl Acad Sci U S A. 2014 Nov 25;111(47):16877–82. doi: 10.1073/pnas.1419045111 25389309 PMC4250167

[41] Takeoka A, Vollenweider I, Courtine G, Arber S. Muscle spindle feedback directs locomotor recovery and circuit reorganization after spinal cord injury. Cell. 2014 Dec 18;159(7):1626–39. doi: 10.1016/j.cell.2014.11.019 25525880

[42] Kiehn O. Decoding the organization of spinal circuits that control locomotion. Nat Rev Neurosci. 2016 Apr;17(4):224–38. doi: 10.1038/nrn.2016.9 . 26935168 PMC4844028

[43] Wilkinson KA, Kloefkorn HE, Hochman S. Characterization of muscle spindle afferents in the adult mouse using an in vitro muscle-nerve preparation. PLoS ONE. 2012;7(6):e39140. doi: 10.1371/journal.pone.0039140 . 22745708 PMC3380032

[44] Gerwin L, Haupt C, Wilkinson KA, Kröger S. Acetylcholine receptors in the equatorial region of intrafusal muscle fibres modulate mouse muscle spindle sensitivity. J Physiol. 2019 Apr; 597(7):1993–2006. doi: 10.1113/JP277139 . 30673133 PMC6441882

[45] Salkoff L, Baker K, Butler A, Covarrubias M, Pak MD, Wei A. An essential ’set’ of K+ channels conserved in flies, mice and humans. Trends Neurosci. 1992 May;15(5):161–66. doi: 10.1016/0166-2236(92)90165-5 . 1377421

[46] Romanovsky D, Moseley AE, Mrak RE, Taylor MD, Dobretsov M. Phylogenetic preservation of alpha3 Na+,K+-ATPase distribution in vertebrate peripheral nervous systems. J Comp Neurol. 2007 Feb 20;500(6):1106–16. doi: 10.1002/cne.21218 . 17183534

[47] Matthews PB. The differentiation of two types of fusimotor fibre by their effects on the dynamic response of muscle spindle primary endings. Q J Exp Physiol Cogn Med Sci. 1962 Oct;47:324–33. doi: 10.1113/expphysiol.1962.sp001616 . 13933877

[48] Kidd GL. Excitation of primary muscle spindle endings by beta-axon stimulation. Nature. 1964 Sept 19;203:1248–51. doi: 10.1038/2031248a0 . 14231794

[49] Bessou P, Emonet-Dénand F, Laporte Y. Motor fibers innervating extrafusal and intrafusal muscle fibers in the cat. J Physiol. 1965;180(3):649–72. doi: 10.1113/jphysiol.1965.sp007722 . 4221243 PMC1357408

[50] Rymer WZ, Grill SE. Reflex excitation of beta and gamma motoneurones in The muscle spindle In: Boyd IA, Gladden MH, editors. London: The McMillian Press Ltd; 1985. chap. 44, p. 303–8.

[51] Manuel M, Zytnicki D. Alpha, beta and gamma motoneurons: functional diversity in the motor system’s final pathway. J Integr Neurosci. 2011 Sep;10(3):243–76. doi: 10.1142/S0219635211002786 . 21960303

[52] Stark R, Grzelak M, Hadfield J. RNA sequencing: the teenage years. Nat Rev Genet. 2019;20(11):631–56. doi: 10.1038/s41576-019-0150-2 . 31341269

[53] Ross CF, Blob RW, Carrier DR, Daley MA, Deban SM, Demes B, et al. The evolution of locomotor rhythmicity in tetrapods. Evolution. 2013 Apr;67(4):1209–17. doi: 10.1111/evo.12015 . 23550769

[54] Madisen L, Zwingman TA, Sunkin SM, Oh SW, Zariwala HA, Gu H, et al. A robust and high-throughput Cre reporting and characterization system for the whole mouse brain. Nat Neurosci. 2010 Jan;13(1):133–40. doi: 10.1038/nn.2467 . 20023653 PMC2840225

[55] Rossi J, Balthasar N, Olson D, Scott N, Berglund E, Lee CE, et al. Melanocortin-4 receptors expressed by cholinergic neurons regulate energy balance and glucose homeostasis. Cell Metab. 2011 Feb 2;13(2):195–204. doi: 10.1016/j.cmet.2011.01.010 . 21284986 PMC3033043

[56] Li L, Yun SH, Keblesh J, Trommer BL, Xiong H, Radulovic J, et al. Egr3, a synaptic activity regulated transcription factor that is essential for learning and memory. Mol Cell Neurosci. 2007 May;35(1):76–88. doi: 10.1016/j.mcn.2007.02.004 . 17350282 PMC2683345

[57] Chen J, Nathans H. Estrogen-related receptor beta/NR3B2 controls epithelial cell fate and endolymph production by the stria vascularis. Dev Cell. 2007 Sept; 13(3):325–37. doi: 10.1016/j.devcel.2007.07.011 . 17765677

[58] Wang L, Klein R, Zheng B, Marquardt T. Anatomical coupling of sensory and motor nerve trajectory via axon tracking. Neuron. 2011 Jul 28;71(2):263–77. doi: 10.1016/j.neuron.2011.06.021 . 21791286

[59] Gallarda B, Bonanomi D, Müller D, Brown A, Alaynick WA, Lemke G, et al. Segregation of axial sensory and motor pathways through heterotypic trans-axonal signaling. Science. 2008 Apr 11;320(5873):233–36. doi: 10.1126/science.1153758 . 18403711 PMC3158657

[60] Wang L, Mongera A, Bonanomi D, Cyganek L, Pfaff SL, Nüsslein-Volhard C, et al. A conserved axon type hierarchy governing peripheral nerve assembly. Development. 2014 May;141(9):1875–83. doi: 10.1242/dev.106211 . 24700820 PMC13148187

[61] Fogarty MJ, Hammond LA, Kanjhan R, Bellingham MC, Noakes PG. A method for the three-dimensional reconstruction of NeurobiotinTM-filled neurons and the location of their synaptic inputs. Front Neural Circuits. 2013 Oct;7:153. doi: 10.3389/fncir.2013.00153 . 24101895 PMC3787200

[62] Muramoto T, Mendelson B, Phelan KD, Garcia-Rill E, Skinner RD, Puskarich-May C. Developmental changes in the effects of serotonin and N-methyl-D-aspartate on intrinsic membrane properties of embryonic chick motoneurons. Neuroscience. 1996;75(2):607–18. doi: 10.1016/0306-4522(96)00185-6 . 8931023

[63] Miles GB, Dai Y, Brownstone RM. Mechanisms underlying the early phase of spike frequency adaptation in mouse spinal motoneurones. J Physiol. 2005 Jul 15;566(Pt 2):519–32. doi: 10.1113/jphysiol.2005.086033 . 15878947 PMC1464745

[64] Mitra P, Brownstone RM. An in vitro spinal cord slice preparation for recording from lumbar motoneurons of the adult mouse. J Neurophysiol. 2012 Jan;107(2):728–741. doi: 10.1152/jn.00558.2011 . 22031766

[65] Nakanishi ST, Whelan PJ. Diversification of intrinsic motoneuron electrical properties during normal development and botulinum toxin-induced muscle paralysis in early postnatal mice. J Neurophysiol. 2010 May;103(5):2833–45. doi: 10.1152/jn.00022.2010 . 20457856

[66] Manuel M, Iglesias C, Donnet M, Leroy F, Heckman CJ, Zytnicki D. Fast Kinetics, High-Frequency Oscillations, and Subprimary Firing Range in Adult Mouse Spinal Motoneurons. J Neurosci. 2009 Sept 9;29(36):11246–256. doi: 10.1523/JNEUROSCI.3260-09.2009 . 19741131 PMC2785440

[67] Amende I, Kale A, McCue S, Glazier S, Morgan JP, Hampton TG. Gait dynamics in mouse models of Parkinson’s disease and Huntington’s disease. J Neuroeng Rehabil. 2005 Jul 25;2:20. doi: 10.1186/1743-0003-2-20 . 16042805 PMC1201165

[68] Grapov D. DeviumWeb: Dynamic Multivariate Data Analysis and Visualization Platform. v0.3.2; 2014 Nov 25. Available from: https://zenodo.org/record/12879#.Y48fmbLMJyY doi: 10.5281/zenodo.12879

[69] R Core Team. R: A language and environment for statistical computing. R Foundation for Statistical Computing; 2015. Available from: https://www.r-project.org/.

[70] Wehrens R. Chemometrics with R: Multivariate Data Analysis in the Natural Sciences and Life Sciences. Heidelberg: Springer; 2011. doi: 10.1007/978-3-642-17841-2

[71] Bourane S, Grossmann KS, Britz O, Dalet A, Del Barrio MG, Stam FJ, et al. Identification of a spinal circuit for light touch and fine motor control. Cell. 2015 Jan 29;160(3):503–15. doi: 10.1016/j.cell.2015.01.011 . 25635458 PMC4431637

[72] Woo S-H, Lukacs V, De Nooij CJ, Zaytseva D, Criddle CR, Francisco A, et al. Patapoutian. Piezo2 is the principal mechanotransduction channel for proprioception. Nat Neurosci. 2015 Dec;18(12):1756–62. doi: 10.1038/nn.4162 . 26551544 PMC4661126

[73] Trichas J, Begbie J, Srinivas S. Use of the viral 2A peptide for bicistronic expression in transgenic mice. BMC Biol. 2008 Sept 15;6:40. doi: 10.1186/1741-7007-6-40 . 18793381 PMC2553761

[74] Halder R, Hennion M, Vidal RO, Shomroni O, Rahman R-U, Rajput A, et al. DNA methylation changes in plasticity genes accompany the formation and maintenance of memory. Nat Neurosci. 2016 Jan; 19(1):102–10. doi: 10.1038/nn.4194 . 26656643

[75] Andrews S. FastQC: a quality control tool for high throughput sequence data. 2010. Available from: https://www.bioinformatics.babraham.ac.uk/projects/fastqc.

[76] Langmead B, Salzberg SL. Fast gapped-read alignment with Bowtie 2. Nat Methods. 2012 Mar 4;9 (4):357–59. doi: 10.1038/nmeth.1923 . 22388286 PMC3322381

[77] Li B, Dewey CN. RSEM: accurate transcript quantification from RNA-Seq data with or without a reference genome. BMC Bioinformatics. 2011 Aug 4;12:323. doi: 10.1186/1471-2105-12-323 . 21816040 PMC3163565

[78] Yates A, Akanni W, Amode MR, Barrell D, Billis K, Carvalho-Silva D, et al. Ensembl 2016. Nucleic Acids Res. 2016 Jan 4;44(D1):D710–16. doi: 10.1093/nar/gkv1157 . 26687719 PMC4702834

[79] Love MI, Huber W, Anders S. Moderated estimation of fold change and dispersion for RNA-seq data with DESeq2. Genome Biol. 2014;15(12):550. doi: 10.1186/s13059-014-0550-8 . 25516281 PMC4302049

[80] Huang D, Sherman BT, Lempicki RA. Systematic and integrative analysis of large gene lists using DAVID bioinformatics resources. Nat Protoc. 2009;4(1):44–57. doi: 10.1038/nprot.2008.211 . 19131956

[81] Ovcharenko I, Nobrega MA, Loots GG, Stubbs L. ECR Browser: a tool for visualizing and accessing data from comparisons of multiple vertebrate genomes. Nucleic Acids Res. 2004 Jul 1;32(Web Server issue):W280–86. doi: 10.1093/nar/gkh355 . 15215395 PMC441493

[82] Mathelier A, Fornes O, Arenillas DJ, Chen CY, Denay G, Lee J, et al. JASPAR 2016: a major expansion and update of the open- access database of transcription factor binding profiles. Nucleic Acids Res. 2016 Jan 4;44(D1):D110–15. doi: 10.1093/nar/gkv1176 . 26531826 PMC4702842

