# Supplementary figures and images for: ERR2 and ERR3 promote the development of gamma motor neuron functional properties required for proprioceptive movement control
Source: PLoS Biol. 2022 Dec 21;20(12):e3001923. doi: 10.1371/journal.pbio.3001923 (PMC9815657; doi:10.1371/journal.pbio.3001923)

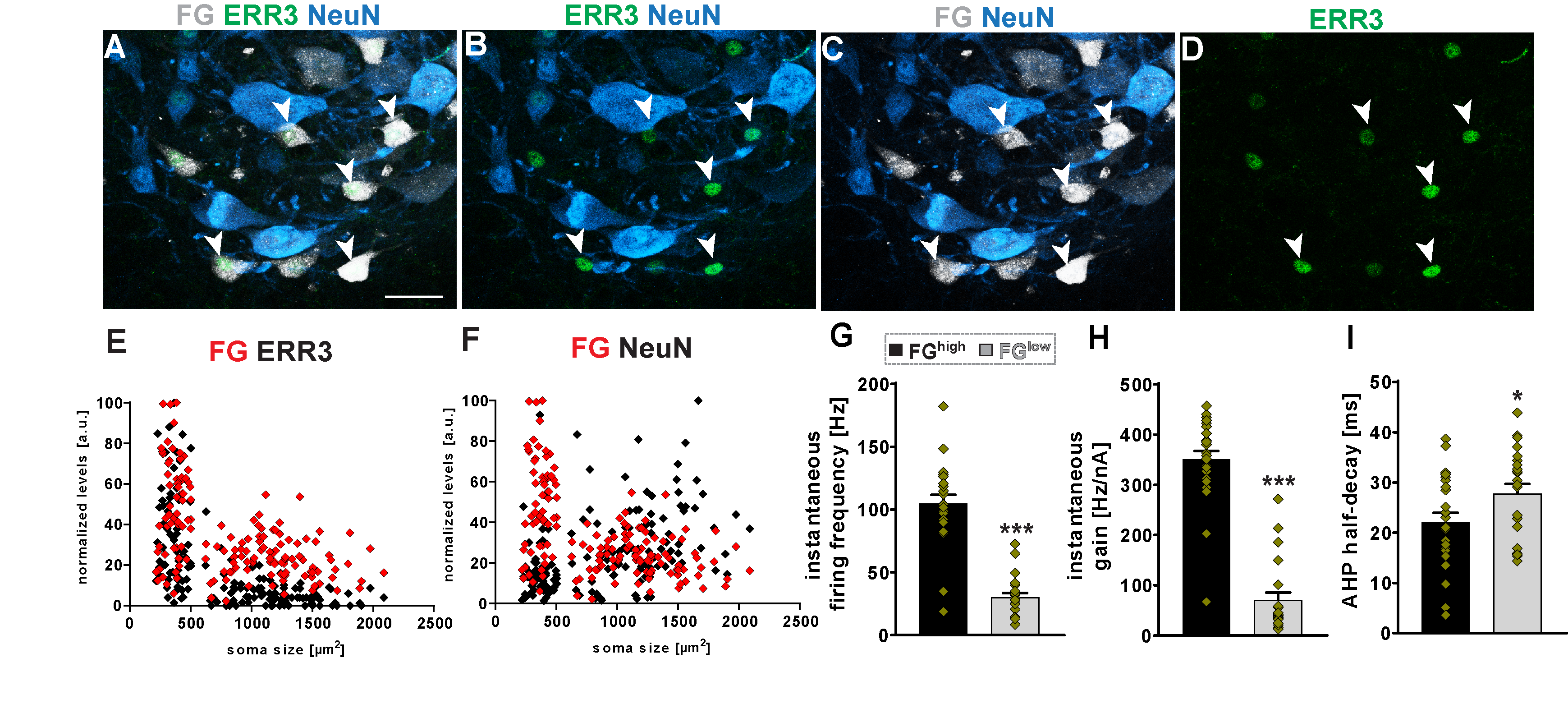

Supplement: S1 Fig — (A-D) P14 spinal cord ventral horn (note: same specimen as in S3G–S3I Fig): gamma and alpha motor neurons can be distinguished by different levels of Fluoro-Gold (FG) incorporation and soma sizes (scale bar: 50 μm). (A) Arrowheads: examples of small soma-size motor neurons with high levels of FG incorporation expressing low or negligible levels of NeuN. (B) High levels of the gamma motor neuron marker ERR3 [27] in the FGhigh, NeuNlow motor neurons (arrowheads). (C) FGhigh motor neurons are NeuNlow (arrowheads) (D) and ERR3high (arrowheads). (E, F) Scatter plots of motor neuron fluorescence levels over soma sizes at P21 (n = 159) (note: data from same experiment depicted in S4Q and S4R Fig): identification of gamma motor neurons based on a combination of soma size and FG levels. (E, F) Gamma motor neurons with small somas and relatively high FG levels that express high ERR3 levels (E), but low or negligible levels of NeuN (F). Larger motor neurons with lower but substantial FG levels express low or negligible levels of ERR3 (E), but mostly higher levels of NeuN (F). (G-I) Control FGhigh (gamma) motor neurons (black bars) exhibit significantly higher instantaneous firing frequency (G), instantaneous gain (H), and lower AHP-half decay time (I) when compared to FGlow (alpha) motor neurons (gray bars). Statistically significant differences are indicated as: *p < 0.05, **p < 0.01, ***p < 0.001, n.s. = not significant, Student t test). Data for S1G-S1I Fig can be found in S1 Data and for S1E and S1F Fig in S2 Data. (TIF) [file pbio.3001923.s001.tif]

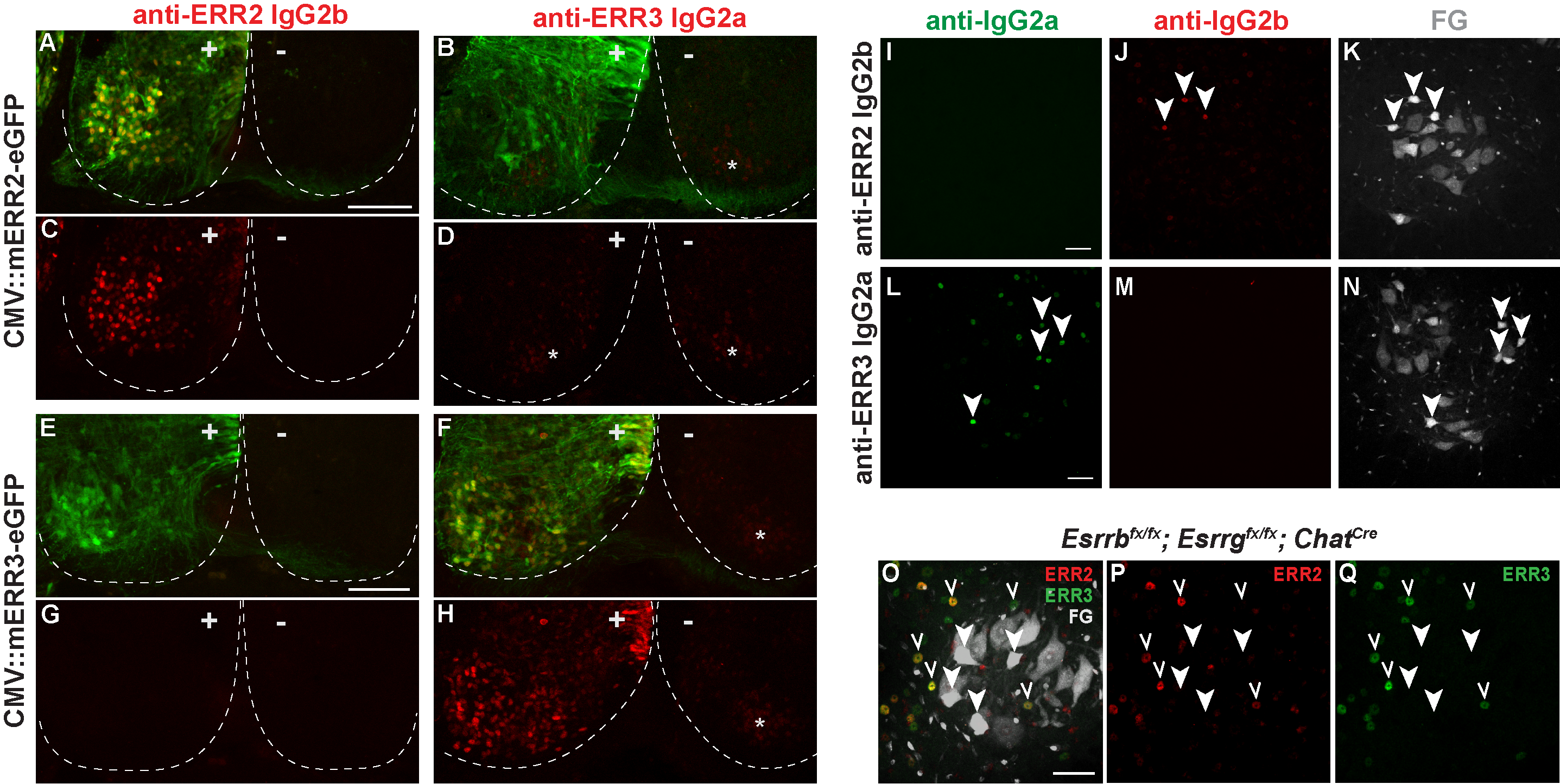

Supplement: S2 Fig — (A-D) Transversal sections of E6 chick spinal cords unilaterally transfected by murine ERR2 (mERR2) and eGFP. (A, C) Immunofluorescence with anti-ERR2 IgG2b detects transfected mERR2 but not endogenous chick ERR2 (cERR2) (scale bar: 100 μm). (B, D) Anti-ERR3 IgG2a detects at low levels of endogenous cERR3 (asterisks) but does not cross-react with transfected mERR2. (E-H) Transversal sections of E6 chick spinal cords unilaterally transfected by murine ERR3 (mERR3) and eGFP. (E, G) Anti-ERR2 IgG2b does not cross-react with mERR3. (F, H) Anti-ERR3 IgG2a detects transfected mERR3 and also detects at low levels of endogenous cERR3 (asterisk in contralateral spinal cord) but does not cross-react with transfected mERR2. (I-N) Transversal sections of P21 mouse spinal cord ventral horn. (I) No cross-reactivity of Alexa488 anti-IgG2a with anti-ERR2 IgG2b (scale bar: 50 μm). (J) Detection of anti-ERR2 IgG2b with Alex555 anti-IgG2b in a subset of motor neuron nuclei (arrowheads; note: lower levels detected in other motor neuron and interneuron nuclei). (K) Fluoro-Gold (FG)-traced motor neurons. Note: characteristic high levels of FG incorporation in small soma size motor neurons (arrowheads). (L) Detection of anti-ERR3 IgG2a with Alexa Fluor 488 anti-IgG2a in a subset of motor neuron nuclei (arrowheads; note: lower levels detected in other motor neuron and moderate-to-high levels in interneuron nuclei) (scale bar: 50 μm). (M) No cross-reactivity of Alexa Fluor 555 anti-IgG2b with anti-ERR3 IgG2a. (N) FG-traced motor neurons. (O-Q) Transversal sections of P21 ERR2/3cko (Esrrbflox/flox; Esrrgflox/flox; ChatCre) mouse spinal cord ventral horn. (O) FG-traced motor neurons overlaid with ERR2 and ERR3 immunoreactivity. Closed arrowheads: small soma size FGhigh motor neurons (scale bar: 50 μm). (P, Q) Absence of ERR2 (P) or ERR3 (Q) immunoreactivity in ERR2/3cko motor neurons (closed arrowheads). Open arrowheads: ERR2 and ERR3 expression is retained in interneurons in ERR2/3cko. (TIF) [file pbio.3001923.s002.tif]

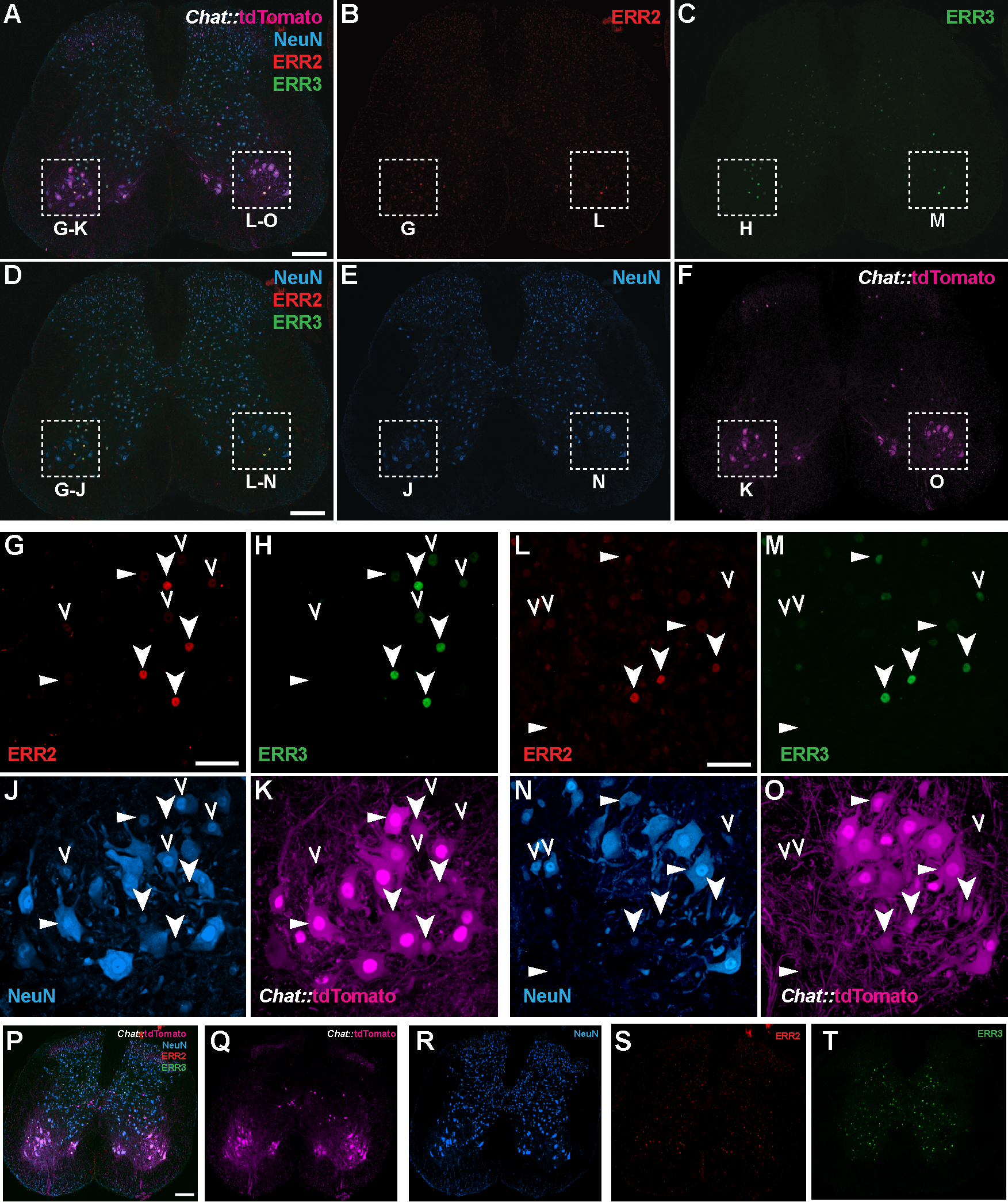

Supplement: S3 Fig — (A-F) Overview of transversal section of P21 Chat::tdTomato (ChatCre; Rosa26fxtdTomato) lumbar mouse spinal cord. (A) Expression of tdTomato, NeuN, ERR2, and ERR3. tdTomato (magenta) mostly labels motor neurons in lamina IX but also subsets of cholinergic interneurons in laminae III and X (see S4A Fig) (scale bar: 200 μm). High levels of ERR2 and ERR3 coexpression in tdTomato+ NeuNlow motor neurons (yellow-orange nuclei in boxed areas). Boxed areas around left and right lateral motor columns correspond to the higher magnification given in G-K and L-O, respectively. (B) Highest levels of ERR2 in motor neurons (boxed area), relatively lower but detectable levels in other motor neuron subtypes and subsets of interneurons throughout the spinal cord. (C) High levels of ERR3 in motor neurons (boxed area), lower but significant levels in other motor neuron subtypes, and occasional high levels in interneurons in the intermediate spinal laminae. (D) High levels of ERR2 and ERR3 coexpression in NeuNlow motor neurons (yellow-orange nuclei in boxed areas) (scale bar: 200 μm). (E, F) Separate channels depicting expression of NeuN only (E) or tdTomato only (F). (G-K) Higher magnification of left ventral horn (boxed area) in (A-F). Closed arrowheads: Highest levels of ERR2 (G) and ERR3 (H) in consistently NeuNlow (J) and tdTomato+ (K) small motor neurons (scale bar: 50 μm). Note that small NeuNlow motor neurons consistently exhibit lower tdTomato levels compared to large motor neurons (K, O). Open arrowheads: low but detectable ERR2 (G) and ERR3 (H) levels in tdTomato− interneurons (K). Triangles: low but detectable ERR2 (G) and ERR3 (H) levels in large tdTomato+ motor neurons (K) with intermediate to high NeuN levels (J). Note that NeuN levels vary considerably in large motor neuron subtypes (see Fig 2B and 2F). (L-O) Higher magnification of right ventral horn (boxed area) in (A-F). Closed arrowheads: Highest levels of ERR2 (L) and ERR3 (M) in consistently NeuNlow (N) and tdTomat [file pbio.3001923.s003.tif]

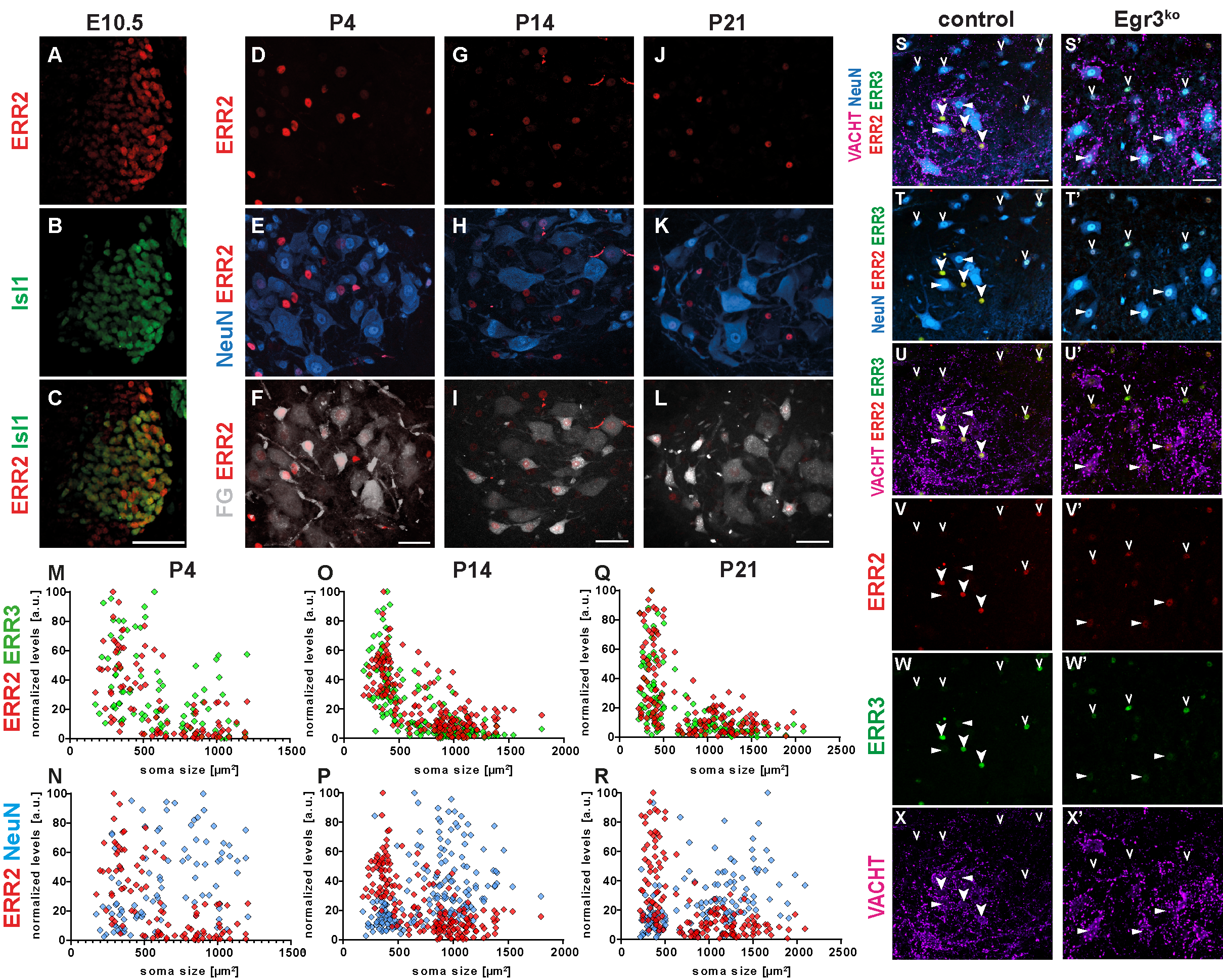

Supplement: S4 Fig — (A-C) Expression of ERR2 in most Isl1+ motor neurons in E10.5 mouse embryo thoracic neural tube (scale bar: 50 μm). (D-F) At P4 higher levels of ERR2 (D) have begun to be mostly confined to small NeuNlow or negligible (E) motor neurons, while many NeuNhigh motor neurons still retain relatively lower yet substantial ERR2 levels (compare D and E). (F) Fluoro-Gold (FG) tracing to visualize motor neurons. (G-H) At P14, high ERR2 levels (G) are further confined to small NeuNlow or negligible motor neurons (H) with high levels of FG incorporation (I). (J-K) At P21, high ERR2 levels (J) are further confined to small NeuNlow or negligible motor neurons (K) with high levels of FG retention (L), while ERR2 levels in NeuNhigh motor neurons have dropped further compared to earlier postnatal stages (compare D, G, and J). (M-Q) Scatter plots of ERR2 (red data points) and ERR3 (green) levels over soma sizes show increasing confinement of high-levels of ERR2 and ERR3 to a population of small motor neurons during postnatal development ages of P4 (n = 100), P14 (n = 189), and P21 (n = 159). (M-R) Scatter plots of ERR2 (red data points) and NeuN (blue) levels over soma sizes show increasing segregation of motor neurons into ERR2high, NeuNlow and ERR2low, NeuNhigh populations during postnatal development, while also revealing considerably cell-to-cell variability in relative expression levels of ERR2, ERR3, and NeuN. (SS’-XX’) Motor neurons fail to retain high ERR2 and ERR3 levels in mice with defective spindle development. (S-X) Control (transversal section of lumbar spinal cord of adult mouse): expression of high ERR2 (V) and ERR3 (W) levels by VACHT+ (X) NeuNlow or negligible (T) motor neurons (closed arrowheads). Open arrowheads: expression of ERR2 and ERR3 by subsets of VACHT− interneurons. Triangle: low ERR2 and ERR3 levels in some large motor neurons. Note: VACHT+ cytosol identifies motor neurons in lamina IX, while VACHT+ varicosities indicate cholinergic synapses or axons stem [file pbio.3001923.s004.tif]

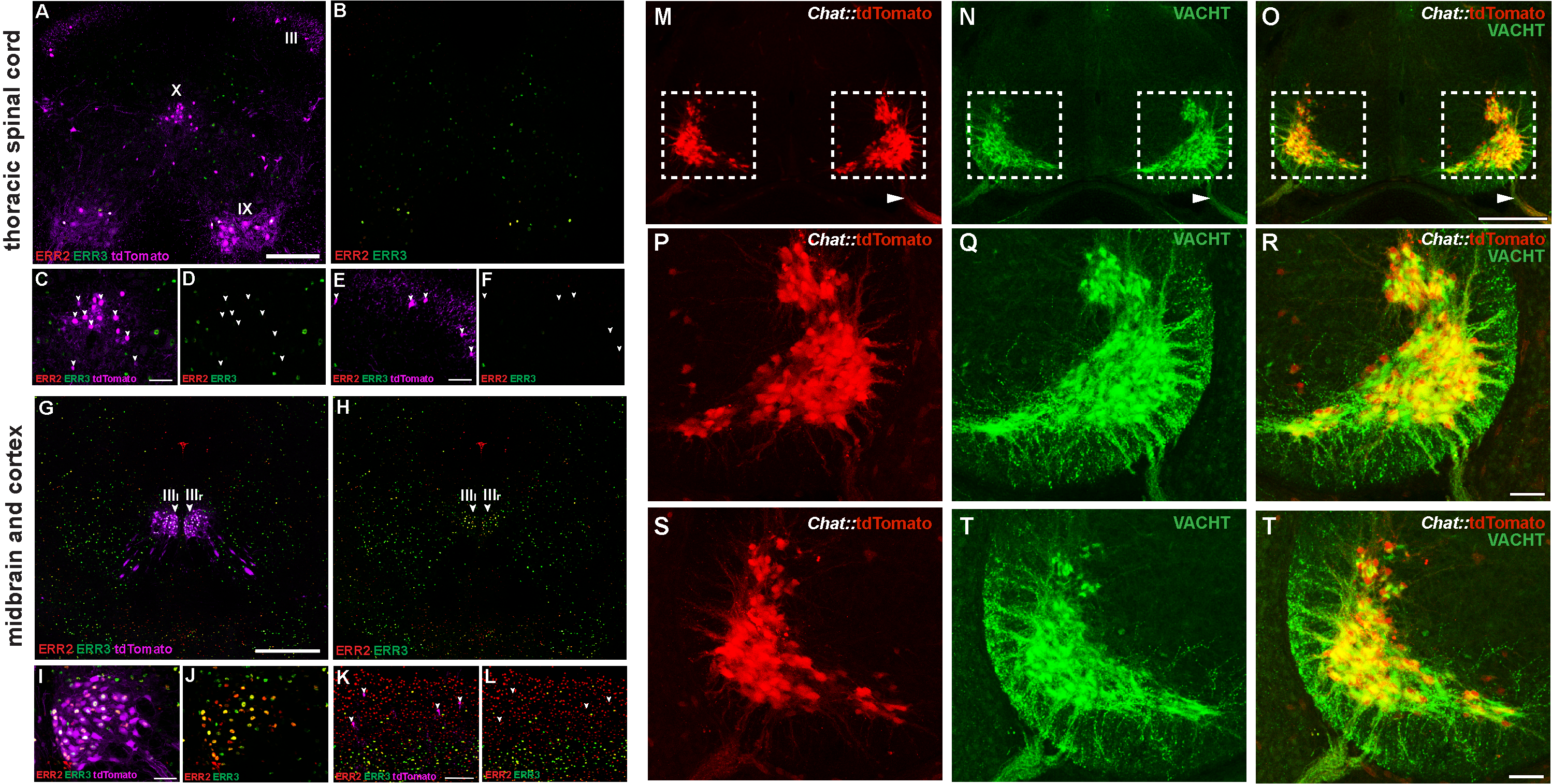

Supplement: S5 Fig — (A, B) Overview of transversal section of P21 Chat::tdTomato (ChatCre; Rosa26fxtdTomato) thoracic mouse spinal cord. (A) Expression of tdTomato indicating ChatCre activity in motor neurons (lamina IX), interneurons (presumably V0c) in lamina X and lamina III interneurons, as well as few scattered neurons in the intermediate laminae (scale bar: 200 μm). (B) ERR2 and ERR3 expression in tdTomato+ motor neurons but not in other tdTomato+ neurons outside lamina IX. (C, D) No detectable ERR2 and ERR3 expression in tdTomato+ lamina X interneurons (arrowheads) (scale bar: 50 μm). (E, F) No detectable ERR2 and ERR3 expression in tdTomato+ lamina III interneurons (arrowheads) (scale bar: 200 μm). (G, H) Expression of ERR2 and ERR3 by oculomotor neurons (III) (arrowheads) and largely nonoverlapping expression of either ERR2 or ERR3 by subsets of noncholinergic neurons throughout the midbrain (scale bar: 500 μm). (I, J) Expression of ERR2 and ERR3 by most but not all cholinergic neurons of the oculomotor nucleus (scale bar: 50 μm). (K, L) Expression of ERR2 and ERR3 in a subset of noncholinergic (tdTomato−) cortical interneurons (scale bar: 100 μm). (M-O) By E12.5, most if not all motor neurons in the ventral horn are tdTomato+ (scale bar: 250 μm). Brackets: enlarged areas shown in (P–T) (scale bar: 50 μm). Green signal: VACHT. (TIF) [file pbio.3001923.s005.tif]

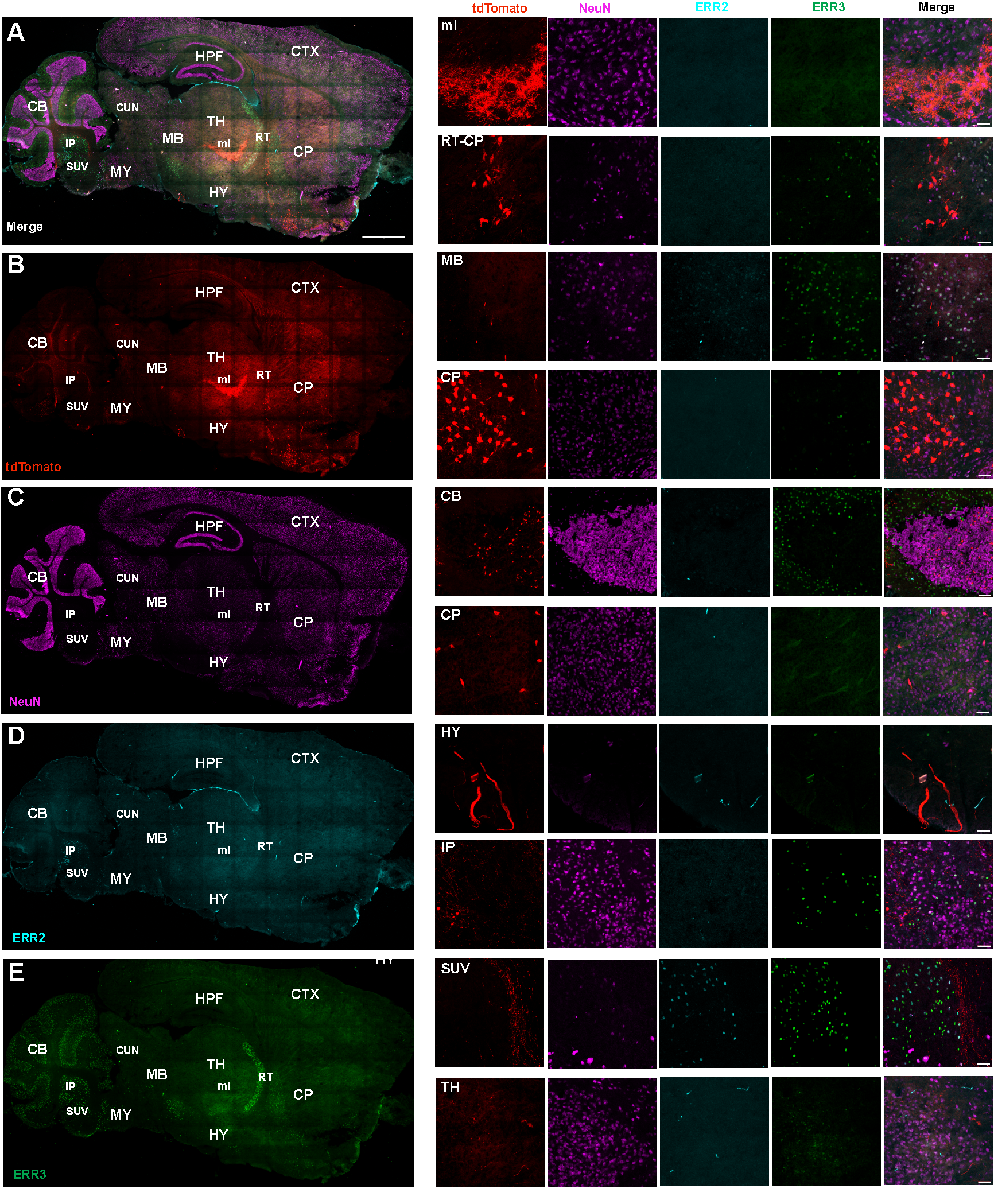

Supplement: S6 Fig — (A-E) Overview of sagittal section of a Chat::tdTomato (ChatCre; Rosa26fxtdTomato) mouse brain assembled from Z-stacked laser scanning microscopy images. ERR2 and ERR3 were co-immunodetected. Details of brain structures indicated in (A-E) are shown next to the overview sections in higher magnification, labelled by the abbreviations for the brain structures pointed out in (A-E) (scale bar: 1,000 μm). Higher-magnification images show consistent nonoverlapping expression of tdTomato and ERR2 and ERR3 (scale bar: 50 μm). (ml, medial lemniscus) tdTomato+ axons passing through area with scattered ERR2/3+cells. (RT, reticular nucleus of the thalamus) Absence of overlap between tdTomato+ and ERR2/3+ cells. (MB, midbrain) Absence of overlap between tdTomato+ and ERR2/3+ cells. (CP, caudoputamen) Absence of overlap between tdTomato+ and ERR2/3+ cells. (CB, cerebellum) Absence of overlap between tdTomato+ (granular cell layer) and ERR2/3+ (Purkinje cell layer) cells. (CB, cerebellum) Absence of overlap between tdTomato+ (granular cell layer) and ERR2/3+ (Purkinje cell layer) cells. (CP, caudoputamen, posterior aspect) Absence of overlap between tdTomato+ and ERR2/3+ cells. (HY, hypothalamus) Absence of overlap between tdTomato+ (fiber tracts) and very few ERR2/3+ cells. (IP, interposed nucleus) Absence of overlap between very few tdTomato+ and ERR2/3+ cells. (SUV, superior vestibular nucleus) Absence of overlap between very few tdTomato+ cells and axons and ERR2/3+ cells. (TH, thalamus) Absence of overlap between very few tdTomato+ cells and axons and scattered ERR2/3+ cells. (TIF) [file pbio.3001923.s006.tif]

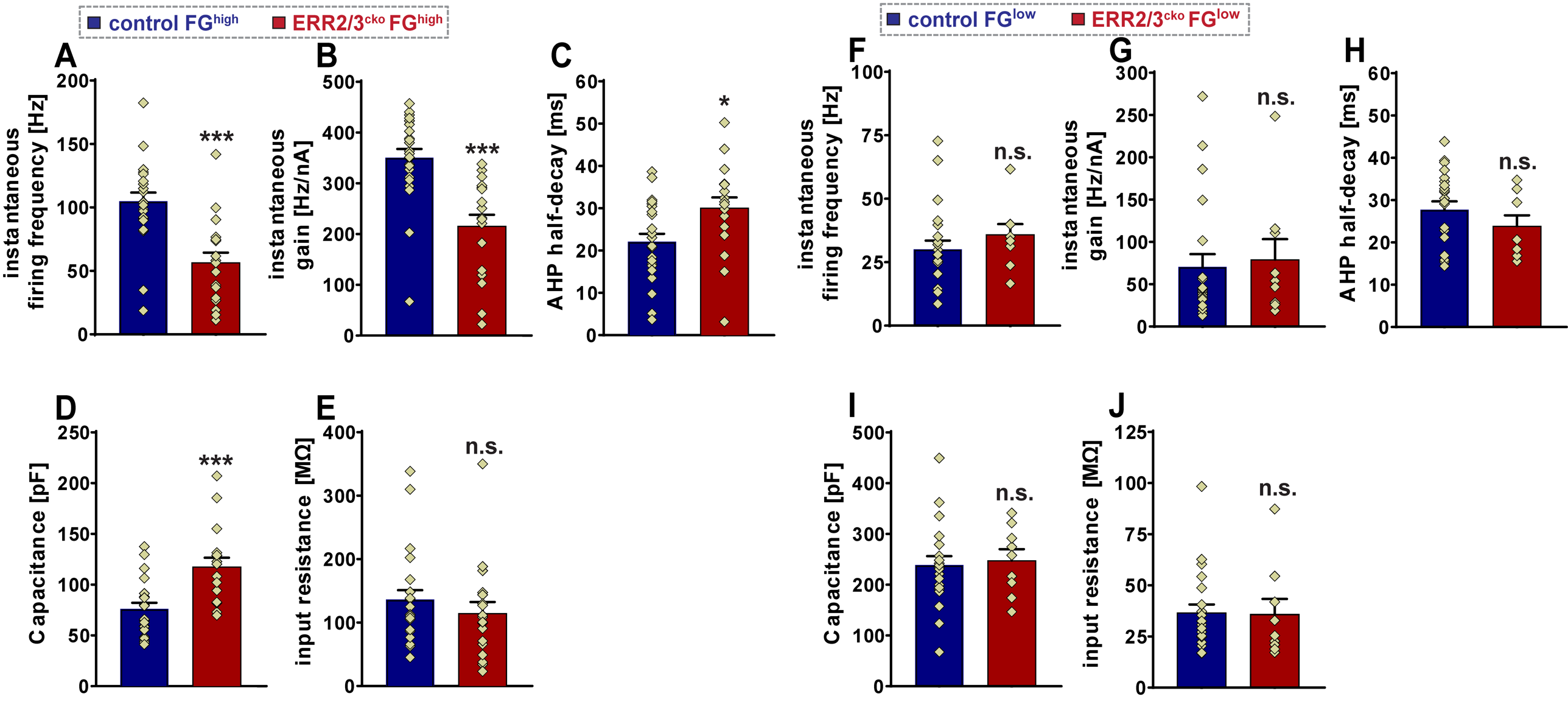

Supplement: S7 Fig — (A-E) ERR2/3cko FGhigh (gamma) motor neurons (red bars) exhibit significantly lower instantaneous firing frequency (A), lower instantaneous gain (B), higher AHP-half decay time (C), higher capacitance (D), while no significant difference input resistance (E), when compared to control FGlow motor neurons (blue bars), respectively (see S1 Table for details). (F-J) No significant differences between ERR2/3cko FGlow (alpha) motor neurons (red bars) and control FGlow (alpha) motor neurons (red bars) when comparing instantaneous firing frequency (F), instantaneous gain (G), AHP-half decay time (H), capacitance (I), and input resistance (J), respectively (see S1 Table for details). Data for S7A-S7J Fig can be found in S1 Data. (TIF) [file pbio.3001923.s007.tif]

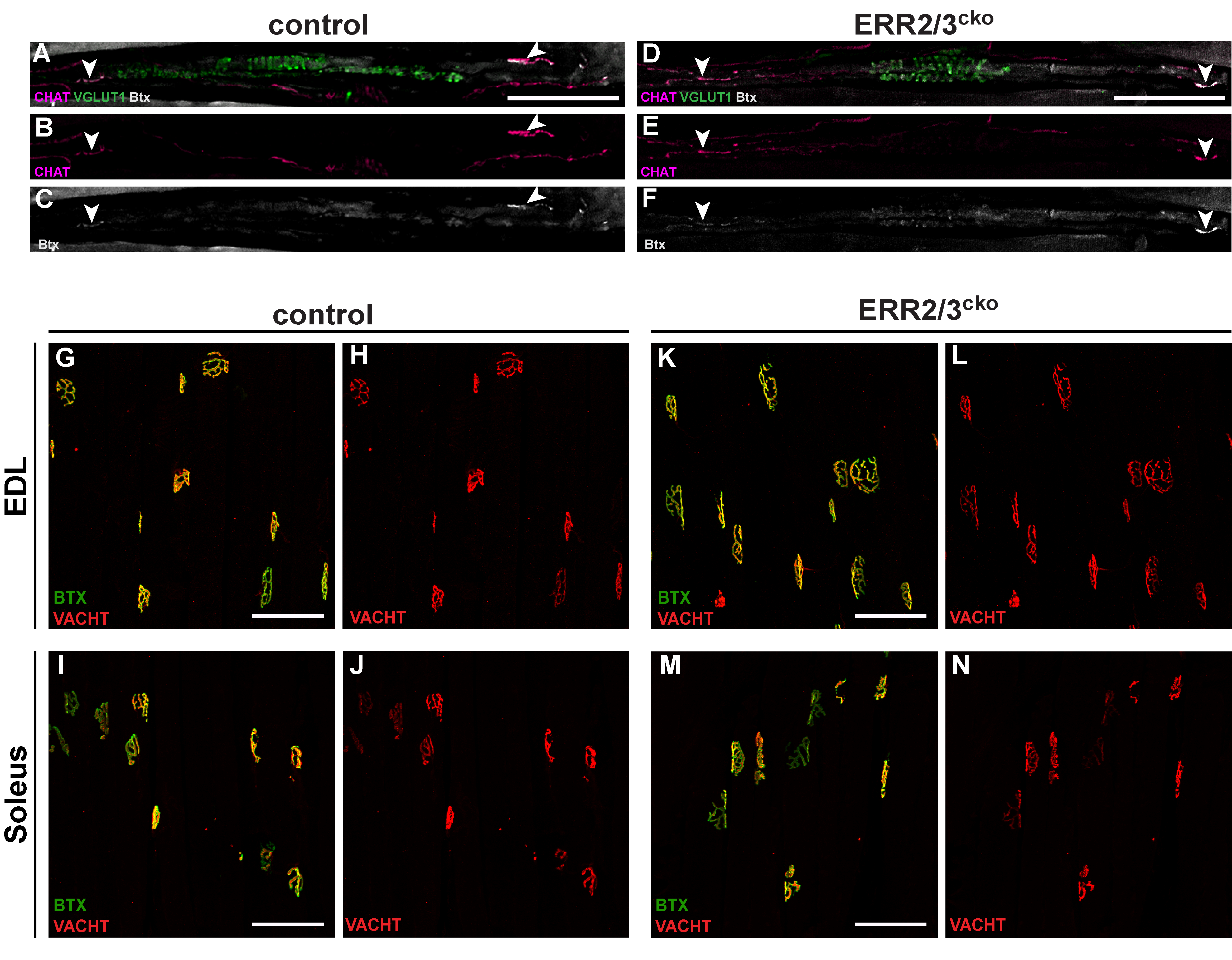

Supplement: S8 Fig — (A-F) P100 mouse soleus muscle spindles of control (heterozygous: Esrrbflox/+;Esrrgflox/+;ChatCre). (A-C) and ERR2/3cko (D-F) mice. (A-F) Comparable distribution of Ia sensory annulospiral endings in the central spindle segment (visualized by VGLUT1, green), motor innervation (CHAT, magenta) and their postsynaptic sites (BTX, alpha bungarotoxin, grey) between control (A-C) and ERR2/3cko (D-F) mice (scale bar: 100 μm). Arrowheads: Comparable motor innervation (CHAT, magenta) of postsynaptic sites on the peripheral segments of intrafusal fibers (BTX, alpha bungarotoxin, grey) in control (A-C) and ERR2/3cko (D-F) mice. Scale bars: 100 μm. (G-N) Sections through EDL (G,H, K, L) and soleus (I, J, M, N) muscle of P100 mice: comparable characteristic pretzel-like morphologies of neuromuscular junctions (NMJs) with extrafusal muscle fibers and consistent motor innervation (VACHT+) of all postsynaptic NMJs (BTX+) and innervation in control (G, H, I, J) and ERR2/3cko (K, L, M, N) mice. Scale bars: 100 μm. (TIF) [file pbio.3001923.s008.tif]

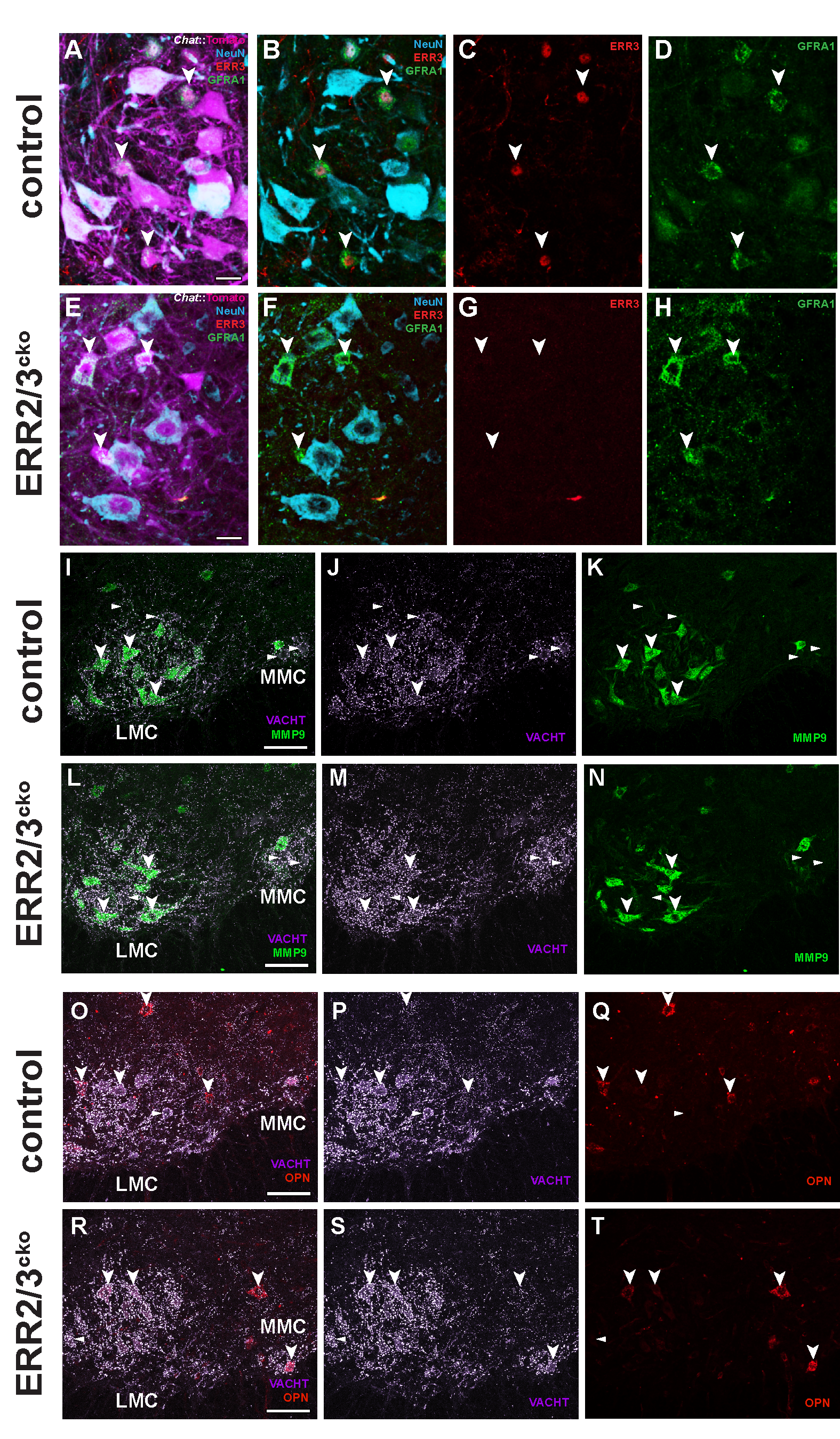

Supplement: S9 Fig — (A-H) Transversal sections of adult Chat::tdTomato lumbar mouse spinal cords of control (A-D) and ERR2/3cko (E-H) mice. (A-D) Expression of high levels of GFRA1 by ERR3high, NeuNlow, and tdTomato+ small soma-size motor neurons (arrowheads), consistently lower levels in larger some-size ERR3high, NeuNlow, and tdTomato+ motor neurons in control mice. (E-F) Persistent expression of high GFRA1 levels in NeuNlow small soma-size motor neurons (arrowheads) in ERR2/3cko mice. (I-T) Transversal sections of adult mouse spinal cords of control (I-K) and ERR2/3cko (L-N) mice. (I-K) High levels of expression of the fast-alpha motor neuron marker MMP9 by large some-size VACHT+ motor neurons in control mice. LMC, lateral motor column; MMC, medial motor column. (L-N) Persistent high MMP9 levels in large some-size VACHT+ motor neurons in ERR2/3cko mice. (O-T) Transversal sections of P400 mice spinal cords of control (O-Q) and ERR2/3cko (R-T) mice. (O-Q) High levels of expression of the slow/intermediate alpha motor neuron marker OPN by subsets of intermediate some-size VACHT+ motor neurons in control mice. (R-T) Persistent high OPN levels in subsets of large some-size VACHT+ motor neurons in ERR2/3cko mice. (Scale bars: 100 μm). (TIF) [file pbio.3001923.s009.tif]

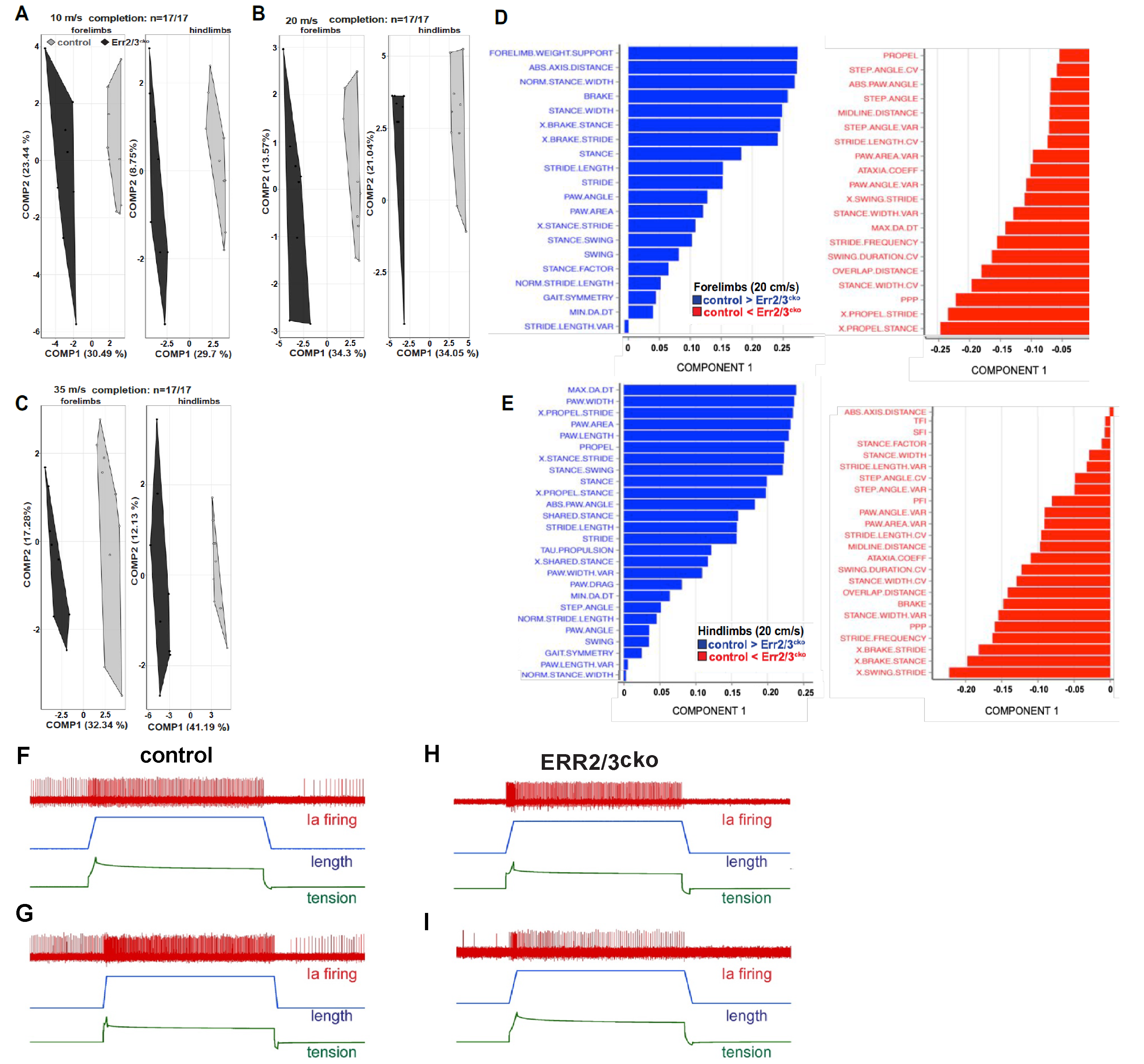

Supplement: S10 Fig — (A-C) Polygon graphs based on partial least squares (PLS) analysis of 58 gait variables measured during treadmill locomotion at 10 m•s−1 (A), 20 m•s−1 (B), and 35 m•s−1 (C). Optimized model prediction was used to assign data sets for fore and hind limbs to either genotype (control versus ERR2/3cko) and the two components of the models were plotted against each other. Each one of the tested animals is represented by a single dot, while polygons group the animals of the same genotype. The amount of between-groups variance explained by each component in the model is expressed in percent of the total between-groups variance. The two components of our optimized models captured more than 25% of the variance in the predictors in both fore and hind limb at all treadmill speeds. These scores indicate that the method was able to capture the maximum variance between genotypes in the first dimension, which is also shown by the absence of overlap between the two groups on the x-axis, together indicating that ERR2/3cko exhibit significant gait alterations compared to control mice. Yet, all ERR2/3cko mice analyzed were able to successfully complete the treadmill locomotion tasks at all speeds tested (provided as the number of animals “n” running until “completion” for each speed). (D, E) Ranking of the variables’ predictive capacities in the forelimb (D) and hind limb (E) models. The most predictive parameters display the highest loadings (arbitrary units) independent of their sign. The mean value of all mice from the same group was calculated for each parameter and depicted in the bar charts. The sign of the loadings only indicates the direction in which gait parameters are affected (increased, red, or reduced, blue, in ERR2/3cko compared to control mice). (F-I) Examples of Ia afferent recordings from extensor digitorum longus (EDL) nerve-muscle preparations. Red traces: Ia afferent firing, blue traces: relative muscle length, green traces: relative muscle tension upon applicatio [file pbio.3001923.s010.tif]

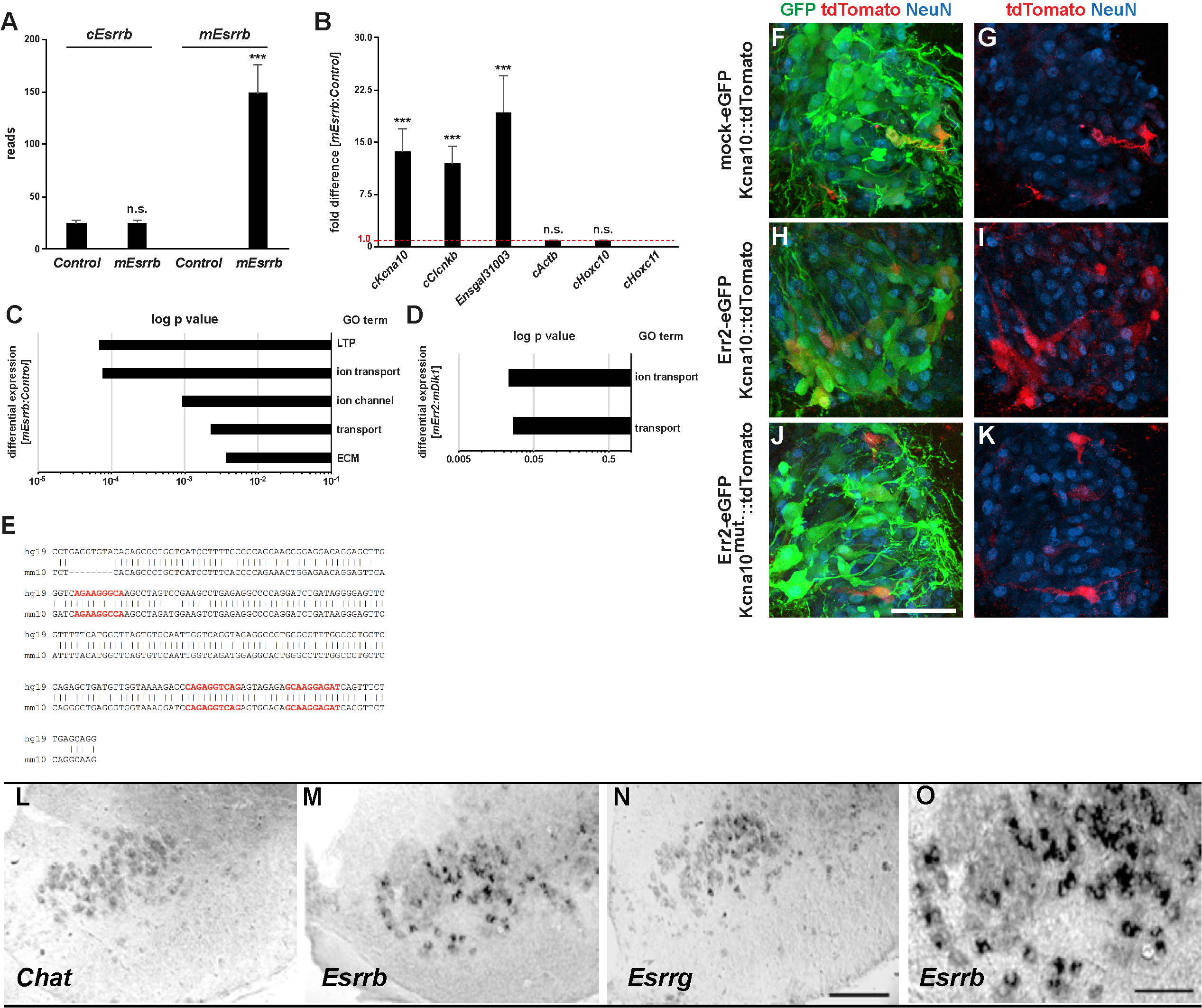

Supplement: S11 Fig — (A) Numbers of RNA reads for endogenous chick Esrrb (cEsrrb) and mouse Esrrb (mEsrrb) in control (eGFP only) expressing chick motor neurons or chick motor neurons expressing mouse Esrrb (mEsrrb): RNA sequencing reveals unaltered cEsrrb (24.60 ± 3.15 versus 24.79 ± 2.45) expression by forced mEsrrb expression, no reads of mEsrrb in the control sample (as expected) and approximately 6-fold overexpression of mEsrrb (148.89 ± 27.03) relative to endogenous cEsrrb levels (24.60 ± 3.15). (B) Examples of genes regulated by ERR2 in chick motor neurons (given in fold change over control motor neurons), including genes encoding the voltage-gated potassium and chloride channels Kcna10 (13.65 ± 3.28) and cClcnkb (11.85 ± 2.51), respectively, as well as the not yet annotated gene Ensgal00000031003 (19.20 ± 5.25). Expression of “structural” gene Actb (0.94 ± 0.05), as well as of Hoxa11 (0.93 ± 0.00) and Hoxc11 (1.03 ± 0.01) related to motor pool identities did not significantly change. (C) Clustered David functional annotations of genes differentially regulated by forced ERR2 in chick motor neurons (“clustered” refers to genes associated with more than one annotated GO term), significance of classification is given as logarithmic p-value. Gene ontology terms include “LTP” (“long-term potentiation”: glutamate receptor Grin2a, neurotrophin receptor Ntrk2, sodium/potassium/calcium exchanger Slc24a2 and tenascin R-encoding Tnr), “ion transport” and “ion channel” (cClcnkb, Kcna10, Grin2a, Slc24a2, potassium channel tetramerization domain containing Kctd4, gamma-aminobutyric acid receptor subunit Gabrd, voltage-gated sodium channel subunit Scn5a, voltage-gated potassium channel Kcnh1, transferrin, TF and Tweety-homolog 3-encoding Ttyh3), “transport” (adding translocase of inner mitochondrial membrane 10 homolog TIMM10, mitochondrial adenine nucleotide translocator SLC25A4 to the “ion transport” list) and extracellular matrix (Tnr, Netrin1 Ntn1 and aggrecan-encoding Acan). (D) GO terms f [file pbio.3001923.s011.tif]

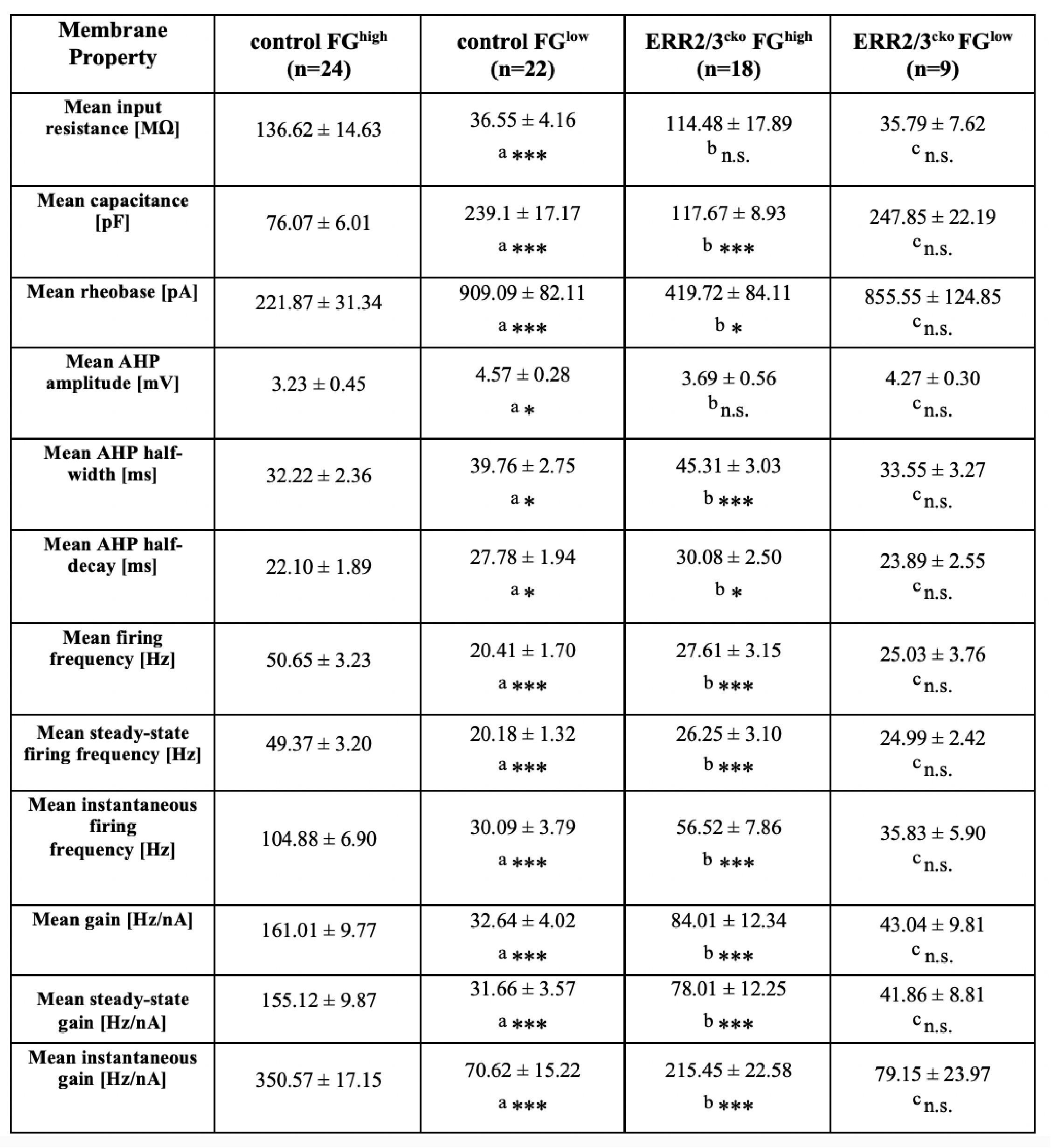

Supplement: S1 Table — Genotypes of the recorded animals are given in the table. Values show mean ± standard error of the mean (SEM). a indicates significant difference between control FGhigh (n = 24) and control FGlow (n = 22) (Student t test); b indicates significant difference between control FGhigh (n = 24) and ERR2/3cko FGhigh (n = 18) (Student t test); c indicates significant difference between control FGlow (n = 22) and ERR2/3cko FGlow (n = 9) (Student t test); ***p-value < 0.001; **p-value < 0.01; *p-value < 0.05; n.s., not significant. n = # of neurons. (TIF) [file pbio.3001923.s012.tif]

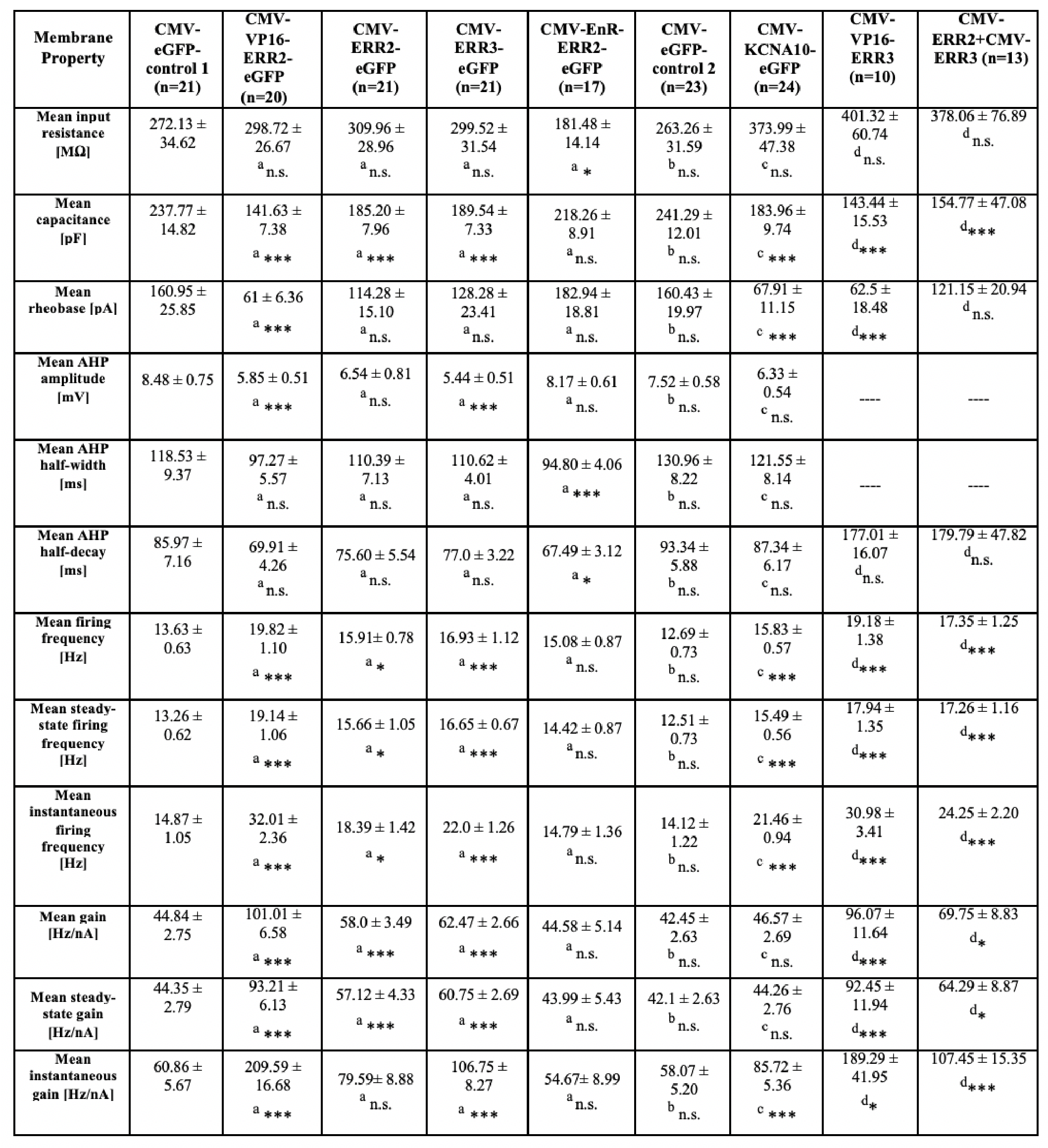

Supplement: S2 Table — Constructs used to stably transfect the motor neurons prior to the recordings are in the table. CMV-eGFP-control 1 versus CMV-VP16-ERR2-eGFP, CMV-ERR2-eGFP, CMV-ERR3-eGFP, CMV-EnR-ERR2-eGFP, and CMV-eGFP-control 2 versus CMV-KCNA10-eGFP. Values show mean ± standard error of the mean (SEM). a indicates significant difference compared to CMV-eGFP-control 1 (Student t test); b indicates significant difference between CMV-eGFP-control 1 and CMV-eGFP-control 2 (Student t test); c indicates significant difference between CMV-eGFP-control 2 and CMV-KCNA10-eGFP; d indicates significant difference compared to CMV-eGFP-control 2 (Student t test); (Student t test); ***p-value < 0.001; **p-value < 0.01; *p-value < 0.05; n.s., not significant. (TIF) [file pbio.3001923.s013.tif]

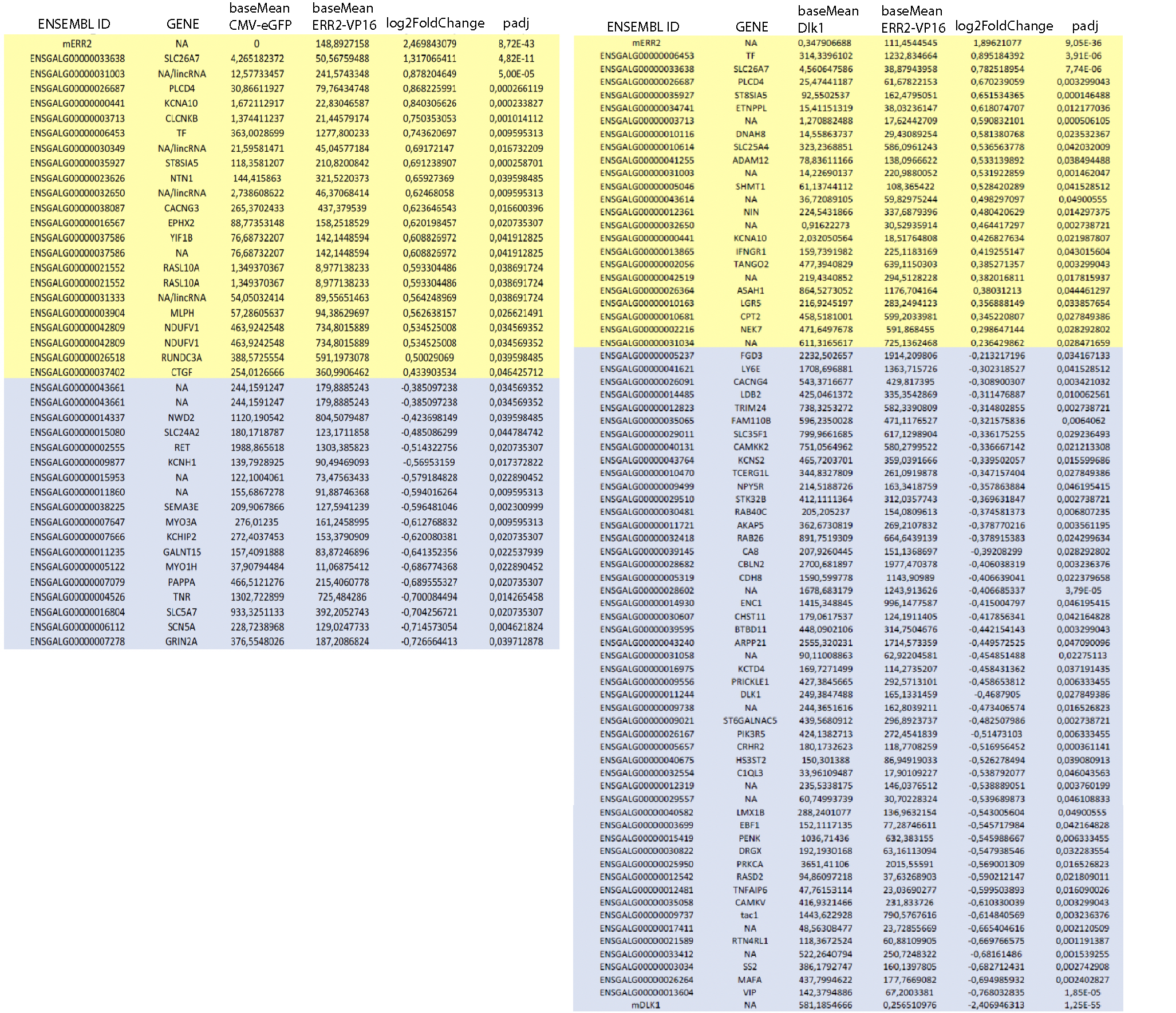

Supplement: S3 Table — (TIF) [file pbio.3001923.s014.tif]
